# Effects of mobile phone electromagnetic fields on brain waves in healthy volunteers

**DOI:** 10.1038/s41598-023-48561-z

**Published:** 2023-12-08

**Authors:** Johan N. van der Meer, Yke B. Eisma, Ronald Meester, Marc Jacobs, Aart J. Nederveen

**Affiliations:** 1 Department of Radiology and Nuclear Medicine, Amsterdam UMC Location AMC, Amsterdam, The Netherlands; 2 Cognitive Robotics, Faculty of Mechanical, Maritime and Materials Engineering (3mE), TU Delft, Delft, The Netherlands; 3 Department of Mathematics, Vrije Universiteit, Amsterdam, The Netherlands

**Keywords:** Risk factors, Neurophysiology

## Abstract

The interaction between biological tissue and electromagnetic fields (EMF) is a topic of increasing interest due to the rising prevalence of background EMF in the past decades. Previous studies have attempted to measure the effects of EMF on brainwaves using EEG recordings, but are typically hampered by experimental and environmental factors. In this study, we present a framework for measuring the impact of EMF on EEG while controlling for these factors. A Bayesian statistical approach is employed to provide robust statistical evidence of the observed EMF effects. This study included 32 healthy participants in a double-blinded crossover counterbalanced design. EEG recordings were taken from 63 electrodes across 6 brain regions. Participants underwent a measurement protocol comprising two 18-min sessions with alternating blocks of eyes open (EO) and eyes closed (EC) conditions. Group 1 (n = 16) had EMF during the first session and sham during the second session; group 2 (n = 16) had the opposite. Power spectral density plots were generated for all sessions and brain regions. The Bayesian analysis provided statistical evidence for the presence of an EMF effect in the alpha band power density in the EO condition. This measurement protocol holds potential for future research on the impact of novel transmission protocols.

## Introduction

Over the past decades numerous studies have been performed to investigate the interaction between biological tissue and electromagnetic fields (EMF) originating from communication devices^1^. All of these devices transmit EMF fields with carrier frequencies ranging from 100 MHz up to 100 GHz in combination with different carrier wave modulations and pulsation frequencies^2^. The presence of these effects is hypothesized to be related to the onset of ill health conditions and is currently subject of fierce societal and political debate^3,4^.

In the Dutch context there are two primary reasons that necessitate further research into the biological effects of EMF. First, a specific question on the health effects of EMF was included in the Dutch science agenda in 2018^5^. Second, with the advent of 5G in 2021, the Dutch health council underlined the need for further research on the effects of fifth generation (5G) telecommunications protocols^6^. Where earlier communication protocols (2G, 3G and 4G) were deemed as safe according to the council, for 5G the council was not convinced that health effects were negligible due to its increased power and higher carrier wave frequencies. In line with this observation, researchers around the globe advocated the need of a moratorium on the further roll-out of 5G systems globally, pending more conclusive research on their safety^4,7^.

Whereas in the past the safety of EMF exposure was determined based on the size of its thermal effects, several researchers have argued that exposure limits should instead be based on the biological effects of EMF on tissue and that the distinction between thermal and non-thermal has limited relevance^7,8^. While the debate on the EMF effects in general focusses on the carrier frequency, the biological effect may as well originate from the modulation of the signal. Adding these modulations to the signal leads to the inclusion of frequencies that are in or near the biological range of frequencies at which the brain operates^9^. It has been suggested that low frequency modulations can have impact on a cellular level in biological systems^10^.

With the advent of 5G and the ongoing debate on the biological significance of EMF exposure, the need for a standardized protocol for assessing EMF effects in vivo is paramount. An essential strand within the literature on detecting the biological effects of EMF in vivo consists of studies that utilize the electroencephalogram (EEG) to record potential EMF induced alterations in the brain's electrical activity. Commonly, the brain is either assessed during rest, with resting-state EEG, or during specific tasks^11,12^, with event-related EEG^13,14^, while the participants are exposed to EMF. A session with EMF dosage and another session without EMF form the basis protocol for the experimental comparison to show the potential presence of EMF effects on the brain.

Several review studies on EEG and EMF have highlighted that there is a lack of standardization in the experimental designs that have been used over the past decade^15,16^. In addition, when the methods of these studies are more closely examined, they reveal several confounding factors in design. These factors can be grossly subcategorized as having to do with the (1) EMF exposure itself, and (2) issues with the experimental paradigm. Issues with the EMF exposure entail an inadequate description of the exposure protocol. In addition, in many studies only mobile phones are used instead of signal generators that allow for a more reproducible application of the signal. Furthermore, if the applied EMF at the location of the head is not accurately measured, the local power density and its spatial variation in the brain remains unknown. Also, in several studies control for background EMF already present in the experimentation room is lacking. Finally, measurement of the possible interaction between the applied EMF on the EEG recording system itself is essential to exclude the presence of EMF induced artefacts in the measured effects. Issues with the experimental paradigm entail the absence of double blinding (of both the participant and the experimenter) and proper counterbalancing of the EMF dosage vs. the no-dosage sessions. In studies that do have counterbalancing the sham and Radiofrequency (RF) condition are commonly split in multiple sessions on separate days. However, this approach may lead to increased variation as resting EEG is heavily biased by events prior to the session^17^.

A common criticism directed towards previous EMF studies is the complexity of the employed statistical methods. Marino et al.^18^ even suggest that ‘many claims result from the use of data-mining to make meaning from the data’. In this work we perform a statistical analysis using a Bayesian approach to counteract this criticism. In short, a Bayesian approach allows for the comparison of the likelihood of the data under various models. In this way we are able to assess the (relative) statistical evidence for the presence of an EMF effect in our data among the hypotheses considered.

In this work we aim to measure the effects of 2G EMF exposure on resting state EEG, while adequately controlling for the aforementioned confounding factors that are related to EMF dosage and experimental paradigm. We fully characterize the EMF protocol, measure the EMF dosage, check the background EMF levels and test whether the EEG hardware itself is affected by the EMF. In terms of experimental paradigm, we use a randomized double-blind counterbalanced crossover design in which each participant is their own control, and all measurements are performed in one session. By using 2G EMF, we can compare our findings with previous literature which primarily uses 2G protocols. We hypothesize that these effects of EMF are subtle, but measurable. We also thereby aim to establish a protocol which can be utilized to assess the effects of other types of EMF, such as 5G, on brain function in future studies.

## Methods

### EMF exposure and background EMF levels

**Figure 1:**
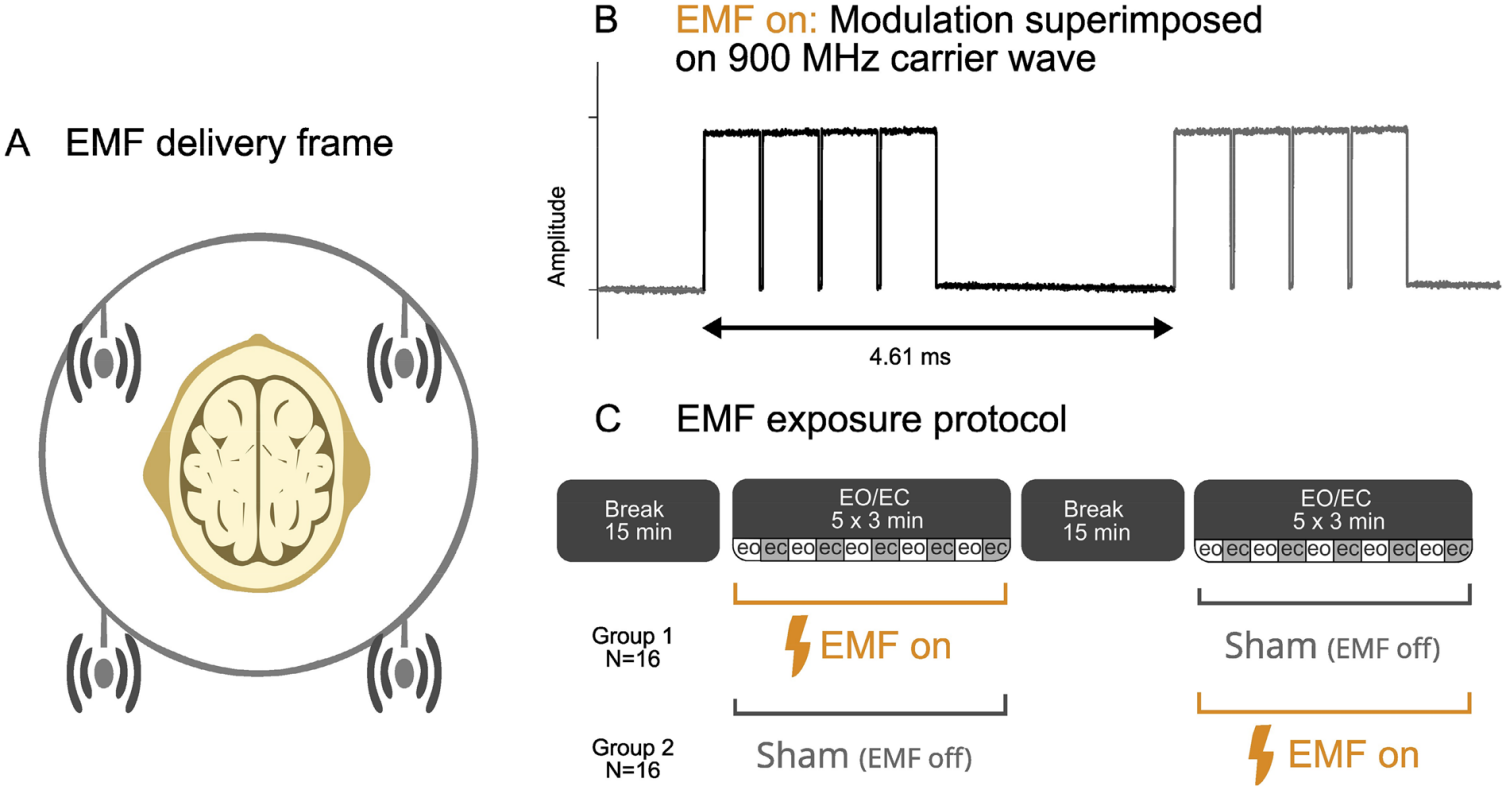
Overview of EMF exposure: (**A**) antenna array consisting of four antennas placed in a rectangular configuration facilitated a homogeneous delivery of EMF in the area in which brain activity was recorded; (**B**) amplitude modulation waveform taken from previous Dutch governmental research^19^; (**C**) experimental protocol consisting of a 2 sessions of a ‘washout’ period of 15 min, followed by Exposure/Sham where eyes open and eyes closed (both 1.5 min) were alternated (5 times).

The EMF experiments were conducted with specific steps to adequately administer EMF dosage. Firstly, we made use of a laboratory located in the hospital's basement, equipped with thick concrete walls to ensure significantly reduced background radiation levels. The EMF was delivered using conventional GSM antennas (Delock, frequency range: 824–2170 MHz). We utilized an antenna array consisting of four antennas to generate a homogeneous area between the antennas at the position of the volunteer’s head (see Fig. 1A). To understand the EMF dosage in our experiment we measured the power density in four different situations:

1. At the location of the hospital hallway outside the laboratory area;
2. At the location of the volunteer;
3. At the location of the volunteer with EMF on;
4. At the location of the volunteer with a mobile phone switched on while answering a call.

Measurements were performed by using a spectrum analyzer (FSH6, Rohde and Schwarz, Germany) equipped with an isotropic antenna (UBB27_G3, Gigahertz Solutions, Langenzenn, Germany) sensitive in the frequency range from 27 MHz till 3.3 GHz. Measurement values were given in dBm per frequency bin and subsequently converted to power densities in μW/m^2^ for the full frequency range applying the antenna factor specified by the vendor and integrating all bins over the entire frequency range. In situation 3 an additional measurement was performed to map the homogeneity of the EMF in the area between the antennas using a handheld broadband RF meter (HF 59B Gigahertz Solutions, Langenzenn, Germany) by moving it around in the area between the antennas at the position of the volunteer’s head.

As EMF signal, we chose the conventional 2G (GSM) protocol that utilizes a carrier frequency of 900 MHz and divides time up into slots that effectively generates a pulsation frequency of 217 Hz. This EMF waveform was also part of the RF protocols delivered in a Dutch government investigation into cognitive effects as reported by the TNO Physics and Electronics Laboratory^19^; see Fig. 1B.

For EMF exposure an Agilent RF signal generator (E44387C) was used that is able to transmit specifically modulated signals with a carrier frequency between 250 kHz and 6 GHz. The output of the signal generator was amplified and fed into the antenna array. The signal generator allowed the 2G EMF we constructed to be switched on or off remotely via Ethernet cable using the Standard Commands for Programmable Instruments (SCPI) interface.

### EEG recordings: equipment validation measurement

EEG was recorded using a 63 (1 electrode was the reference at location FCz) channel active electrode Acticap (Brain Products GmbH, Gilching, Germany) with a sampling rate of 2500 Hz. EEG data were processed and analyzed using Matlab (Release 2021a) and the EEGLAB toolbox^20^. To assess the effects of the EMF exposure on the system itself, regardless of brain activity, we performed EEG measurements using a watermelon as a phantom, in the RF on and RF off conditions. A high sampling rate (2500 Hz) was chosen so as to adequately monitor the 217 Hz 2G component and its harmonics. During analysis the EEG is segmented into 4-s segments, and the average power spectral density (PSD) estimate is calculated over the entire frequency range of 0–1000 Hz along with the standard deviation (calculated over segments) to assess the effects of EMF on the EEG hardware.

### EEG recordings: EMF effects on the brain

To assess effects on the brain, 32 healthy consenting volunteers (age: 23.6, SD 7.3, 11 males) participated in this study, and experiments were approved by the Medical Ethics Review Committee of the Amsterdam UMC according to the Declaration of Helsinki. Participants were recruited via advertisements and received a 25 euros participation fee. The same EEG setup and sampling rate as the equipment validation setup were used. Upon arrival, each participant received instructions for the two upcoming EEG sessions and was assessed being left or right handed using the Edinburgh inventory^21^, and informed consent was signed. The experimental protocol consisted of two sessions of a ‘washout’ period of 15 min, followed by an Exposure/Sham recording where the eyes open and eyes closed conditions (both lasting 1.5 min per condition) were alternated 5 times. After each block of eyes open and eye closed, there was a short break (these self-paced short breaks lasted on average 6 to 16 s and average duration varied across participants; see Supplementary Fig. S65 and Appendix D for the average duration per participant). Subjects were informed that during one of the two sessions, the antennas would be ‘on’. Prior, and in between sessions there was a 15-min break to accommodate a withdrawal of any transient EMF effects (see Fig. 1C). This was done in an automated double-blind procedure; antennae were switched on or off based on a look-up table that was inaccessible to experimenter and participant, which served as input to a python script that sent SCPI commands to the EMF generator. Upon completion of the experiment, this look-up table indicated which participant received the EMF dose in which of the two sessions. Participants were divided into two groups after completion of the experiment; group 1 (subjects 1–16) had an EMF dose during the first session (session A), and group 2 (subjects 17–32) received EMF during the second session (session B); see Fig. 1C.

**Figure 2:**
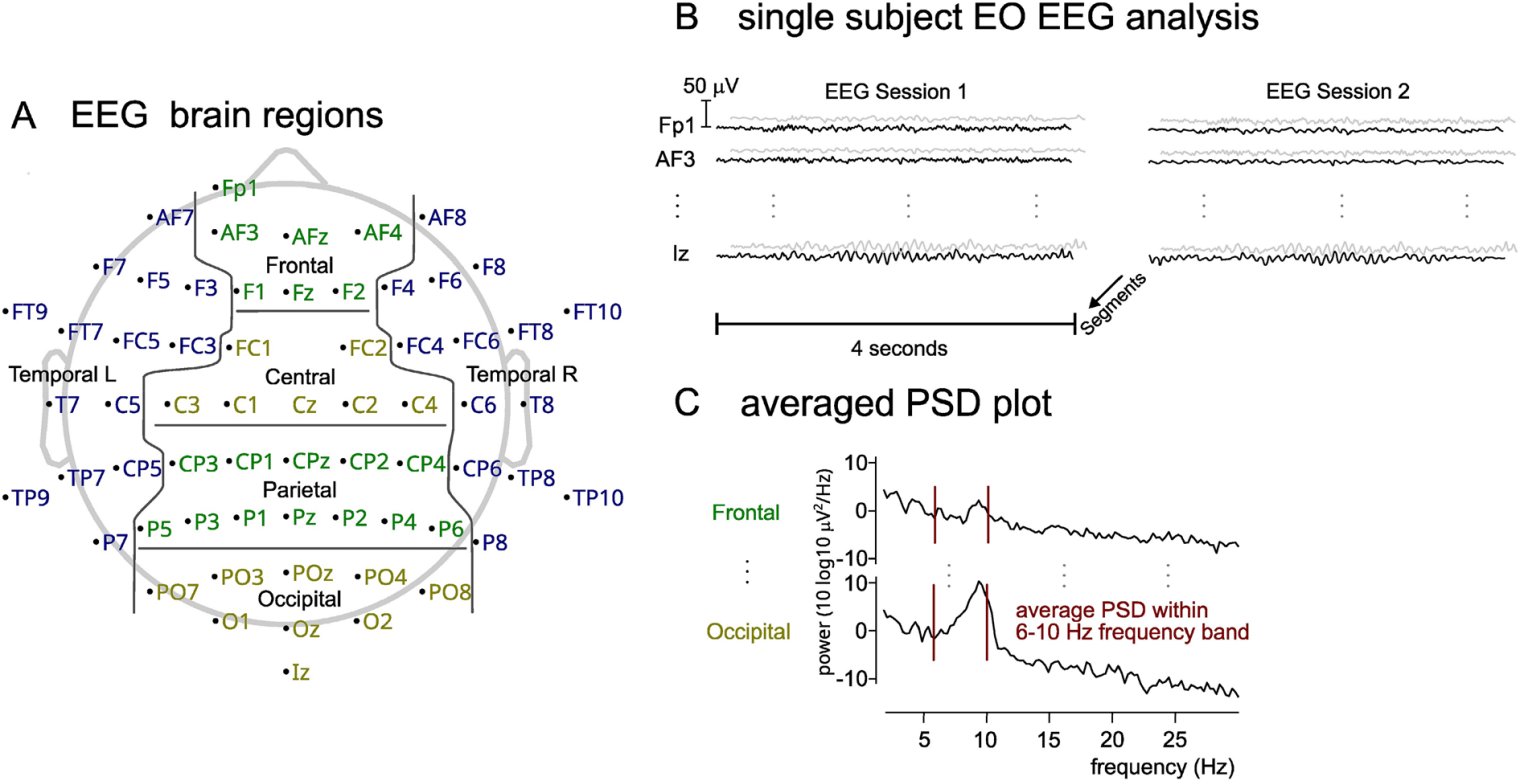
(**A**) Brain regions constituting each averaged region; (**B**) data segmentation, for each channel; (**C**) extraction of PSD information within a frequency band of 6–10 Hz; for each brain region; for each session.

The data were first sampled down to 250 Hz and visually inspected to remove data segments containing artefacts. This was followed by high-pass filtering of 1 Hz, re-referencing to average reference, and decomposition by individual component analysis (infomax ICA) to de-mix the EEG signal into 62 components^22^. Components were classified into artifact and non- artifact components in a semi-automated way using ICLabel^23^, producing a classification level (percentage score) for each type (eyeblink, muscle, line noise, and brain), for each component. After classification, we assessed each component by visual inspection of its power spectrum, classification levels and topographical distribution onto each EEG electrode. On average, about 20% of components were removed. After removal of components, the signals were again re-mixed into a cleaned EEG signal. See Appendix A for more details regarding ICA removal. Finally, the EEG was segmented into 4-second epochs (Fig. 2B) and the average (over epochs) power spectral density (PSD) was estimated for each channel and participant (Fig. 2C). Further averaging was performed over groups of channels to calculate spectra for six regions (see Fig. 1C). The following channels were used for each region: frontal: Fp1, AF3, AFz, AF4, F1, Fz, F2; Left temporal: AF7, F3, F5, F7, FT7, FC5, FC3, C5, T7, TP7, CP5, P7, FT9, TP9; right temporal: AF8, F4, F6, F8, FC4, FC6, FT8, C6, T8, CP6, TP8, P8, FT10, TP10; central: FC1, FC2, C3, C1, Cz, C2, C4; parietal: CP3, CP1, CPz, CP2, CP4, P5, P3, P1, Pz, P2, P4, P6; occipital: PO7, PO3, POz, PO4, PO8, O1, Oz, O2, Iz; see Fig. 2A. PSD plots were calculated in all regions, for the following four conditions: EO + RFon, EO + RFoff, EC + RFon and EC + RFoff.

To statistically assess the effects for EMF on the brain, we exported the data into Rstudio and used the packages: *brms*, *bayestestR*, *tidybayes*, and *emmeans*. Bayesian inference allows for comparing the likelihood of the observed data under alternate models. We performed a Bayesian analysis on the between-session differences of the averaged PSD values, since the change in this value over the sessions could possibly indicate the presence of an EMF effect. More specifically we calculated the PSD contrast for each participant, i.e. the change in PSD over sessions A and B, in a frequency band between 6 and 10 Hz in the EO condition. We assumed that the PSD contrast follows a normal distribution. In our case, we formulated five models (or hypotheses) to explain the data. Within each of the models, we assume that the variance sigma of the PSD contrast does not vary among participants. The simplest model (Model 1) assumes that the PSD contrast (i.e. the change in alpha power between session A and B) has zero mean and has variation sigma. This model would indicate that there is no PSD contrast present in both groups. In Model 2 we assume that the PSD contrast has unknown mean. This model can only be used to detect nonzero PSD contrast assuming that there is no difference between the groups. Model 3 assumes that the PSD contrast differs per group, but not per brain area. In Model 4 it is precisely the other way around, and in this model we assume a separate PSD contrast for each brain area but it does not discriminate between groups. Model 5 combines the last two models and contains separate PSD contrast parameters for each brain area per group**.**

Because the choice of prior plays a role in calculating the posterior density, and we do not possess any prior information, we opted for several diffuse priors of the parameters. For the PSD contrast parameters we chose the Normal distribution (0, 1), the Cauchy distribution (0, 1) and the Uniform distribution (-8, 8). For the sigma parameter, we chose the Uniform distribution (0, 5), the Half-normal distribution (0, 2), and the Gamma distribution (2, 2). By analyzing each possible combination, we obtained nine matrices of 25 comparisons (between two models) each, allowing a sensitivity test in which many different outcomes were possible but should show a similar pattern if the role of the prior is limited.

A Likelihood Ratio (LR) was estimated for each comparison, which is a number showing relative level of support for one model compared to the other, given the data, and the prior assumption on the parameters. A LR value of 1 means that the numerator and denominator model equally support the data given the priors. A LR greater than 1 means that the numerator model explains the data better than the denominator model. A LR value less than 1 means the opposite. Specifically, we computed the likelihood of the observed data under each model (hypothesis), by averaging over the prior assumption on the parameters. For instance, if a parameter is Cauchy distributed, then for each value of that parameter we compute the likelihood of the data, and weight these outcomes according to the Cauchy distribution. Computationally, this can be done in various ways, either by direct computation or by a simulation approximation procedure. The outcome will be the same in both approaches.

We subsequently computed posterior densities of the parameters, which are updated prior densities which describe the knowledge of the parameters after the experiments. To facilitate comparison with previous papers using a frequentist approach, we also provide p-values for the most successful model by performing a classical statistical t-test in this model. To estimate a p-value, we analyzed the observed data using a linear mixed model in which the subject was added as a random variable since we had more than one observation per subject. The p-value was obtained by looking up the estimated t-value and degrees of freedom.

## Results

### EMF exposure and background EMF measurements

**Figure 3:**
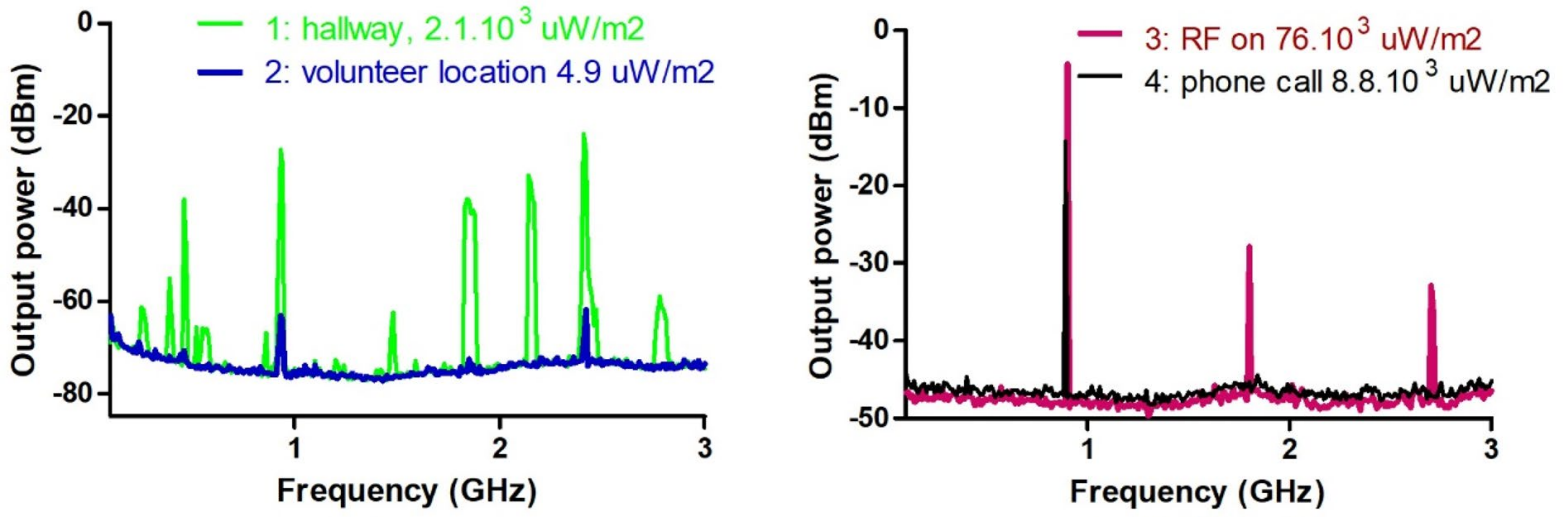
Power spectrums for four different situations and accompanying power densities; left, in green: situation 1—ambient EMF picked up in hallway, with peaks from different devices; mobile phone protocols, WIFI, wireless (DECT) phones. Left, in blue: situation 2—reduced ambient EMF picked up at the volunteer location. Right, in red: situation 3—EMF delivered by our pulse generator, during RF on conditions, at the volunteer location, right, in black: situation 4—typical EMF delivered by a mobile phone at the volunteer location. Note that the right figure (situations 3 and 4) has a different, less sensitive scale compared to the left figure. The reason was that the receiver needed to be set to a lower sensitivity when subjected to EMF to prevent exceeding the maximum sensitivity threshold.

Figure 3 shows the EMF exposure levels in four different situations. At the volunteer location (situation 2) the power density was 3 orders of magnitude lower than in the hallway in the public area of the hospital (situation 1), where multiple frequencies could be measured. When switching on RF via either a mobile phone (situation 4) or a signal generator (situation 3) in the laboratory space at the location of the volunteer one can observe a GSM signal measured at 900 MHz. Variations of the power density between the antennas was limited to a factor of 2, i.e. the power density within the area where the brain of the volunteer was located was roughly uniform (situation 3). The data presented in Fig. 3 emphasize that the ambient EMF levels at the volunteer's location are lower by several orders of magnitude when compared to both the situation with RF radiation on and a typical hospital hallway outside the laboratory area.

### EEG equipment validation

**Figure 4:**
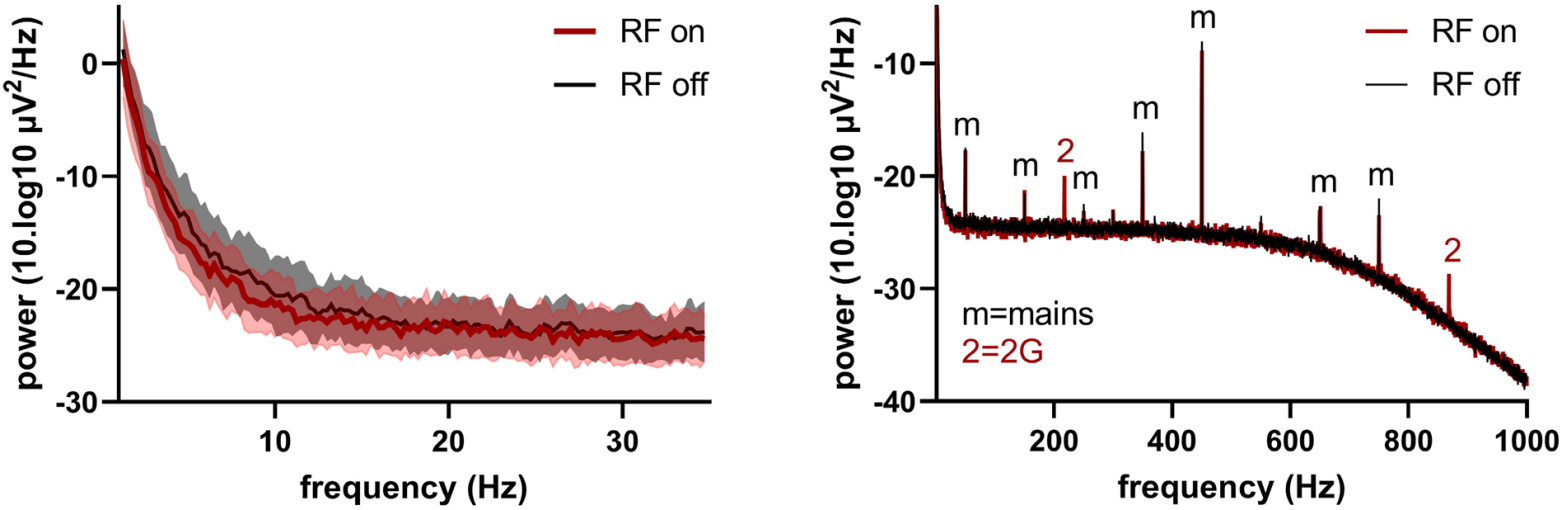
PSD plots of watermelon measurement with RF on and RF off; left: PSD plot in range 0–35 Hz, no differences visible in the physiological range of the signal; right: PSD plot in range 0–1000 Hz, 50 Hz (from the mains) and 217 Hz (from the 2G pulsation) and higher harmonics present in RF on and off signals. Note that both the x-axis and y-axis are scaled differently in the right figure by using a larger range for both axes.

Power plots obtained in the watermelon showed no effect of the EMF, except for the presence of peaks at 217 Hz and 868 Hz, the pulsation frequency and one of its higher harmonics (see Fig. 4). These peaks are outside the physiological frequency range of EEG signals that can be observed in humans (0–50 Hz). In addition in both the RF on and off conditions a 50 Hz peaks from the mains and its higher harmonics are visible (at 150 Hz, 250 Hz, 350 Hz, 450 Hz, 650 Hz and 750 Hz). These frequencies are outside the physiological range. In addition, peaks from both the modulation and the mains are well below the magnitude of measurable human EEG signals. Overall, no interference from the EMF exposure could be detected that could be of relevance to our EEG measurements in the human brain.

### Effects of EMF on brain EEG

**Figure 5:**
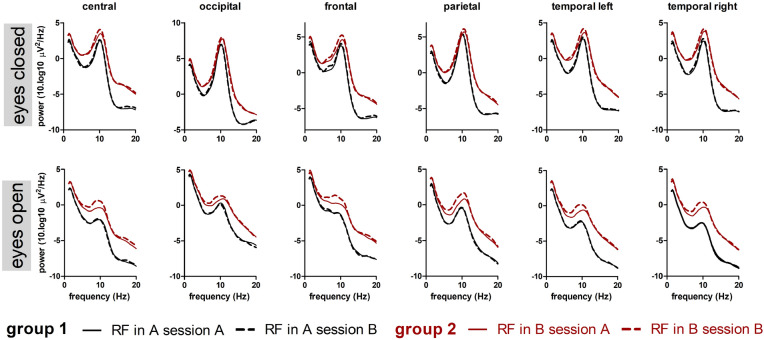
PSD plots in six brain regions for EC and EO conditions. Black lines indicate average PSD over 16 participants in group 1 (receiving RF in session A and sham in session B); red lines average PSD over 16 in participants in group 2 (receiving RF in session B and sham in session A). Continuous lines are the averaged PSD (over participants) from session A; dashed lines are the averaged PSD from session B.

**Figure 6:**
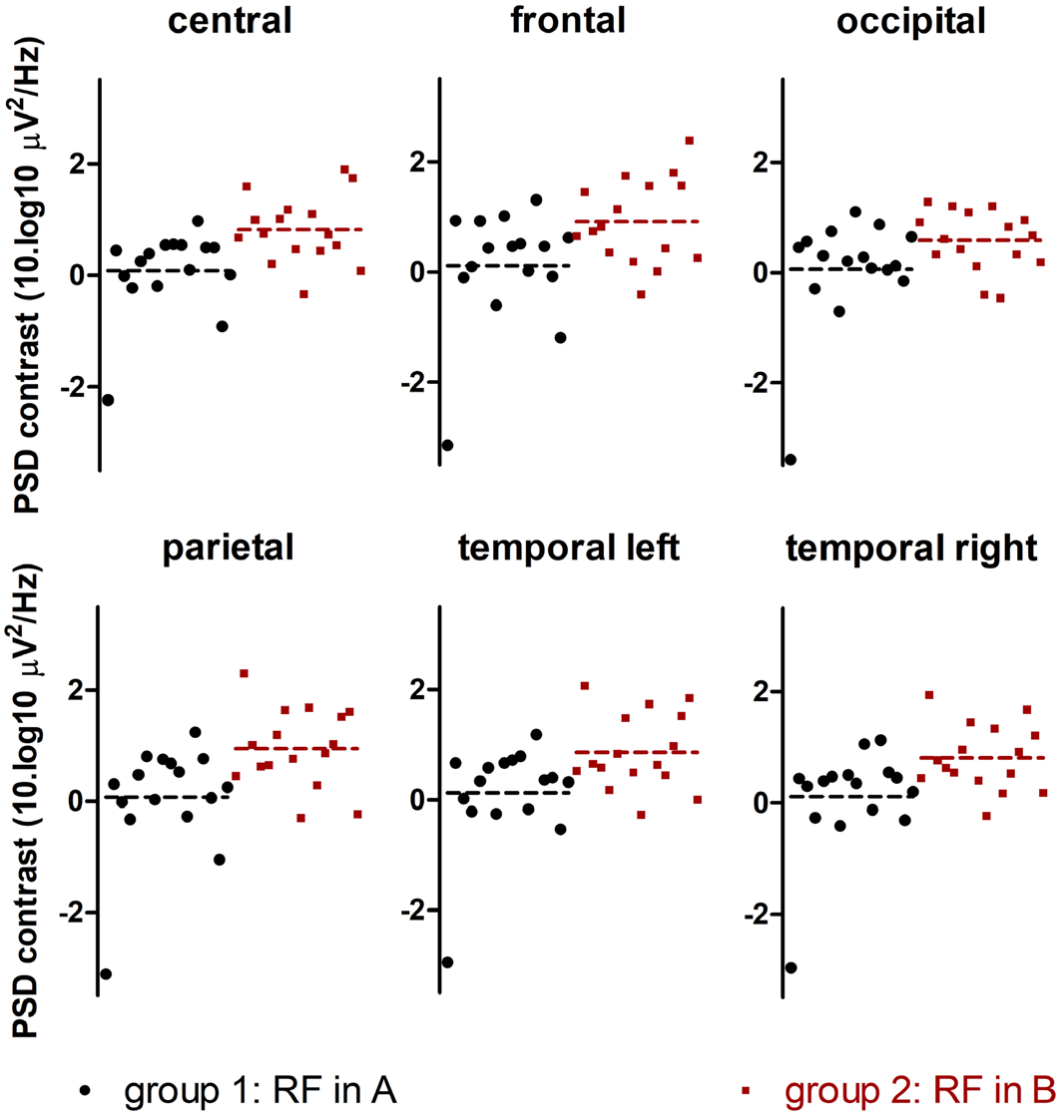
PSD contrast (i.e. difference in averaged EEG power between session A and B in alpha band) in separate brain regions for both groups in the EO condition. Black dots indicate PSD contrast values from group 1 who received RF in session A; red dots indicate PSD contrast values from group 2 who received RF in session B.

Figure 5 shows PSDs for six brain regions for both EC and EO conditions. Each plot contains data from two sessions and two groups, totaling to 4 PSDs per plot. For each group the PSDs in both sessions are similar, except for the EO condition where the two groups differ in the alpha band in case the RF is present in session B. On a per subject level the difference in PSD contrast between both groups is further illustrated in Fig. 6 for the EO condition. The PSD contrasts per subject per region are also shown in Table 1. PSD plots for each subject can be found in Appendix B. The between-subject variability of the power spectra is higher than that displayed by the variability of the PSD contrast as shown in Fig. 6; see Appendix E for individual PSD estimates, overlaid with the average PSD depicted in Fig. 5. Figs 5 and 6 visually illustrate that even though the between-subject PSD differences are large, differences in PSD contrast between group 1 and 2 can still be appreciated. These differences are visible in all brain regions.

**Table 1:** PSD contrasts for all brain regions per subject in groups 1 and 2 in the EO condition.

	Central	Frontal	Occipital	Parietal	Temp. L	Temp. R	Mean		Central	Frontal	Occipital	Parietal	Temp. L	Temp. R	Mean
Group 1	PSD contrast (μV^2^/Hz)							Group 2	PSD contrast (μV^2^/Hz)						
	−2.23	−3.14	−3.39	−3.11	−2.95	−2.96	−2.96		0.69	0.66	0.92	0.46	0.53	0.45	0.62
	0.45	0.94	0.46	0.31	0.67	0.44	0.55		1.59	1.45	1.29	2.31	2.07	1.94	1.78
	−0.01	−0.10	0.57	−0.01	0.02	0.30	0.13		1.00	0.75	0.34	1.02	0.66	0.77	0.76
	−0.23	0.10	−0.30	−0.32	−0.21	−0.26	−0.20		0.75	0.83	0.62	0.63	0.59	0.63	0.68
	0.25	0.93	0.31	0.48	0.34	0.39	0.45		0.21	0.36	1.21	0.66	0.18	0.55	0.53
	0.39	0.44	0.75	0.81	0.58	0.47	0.57		1.02	1.15	0.43	1.20	0.84	0.96	0.93
	−0.19	−0.60	−0.70	0.03	−0.25	−0.41	−0.35		1.19	1.75	1.10	1.65	1.49	1.46	1.44
	0.55	1.02	0.21	0.76	0.67	0.50	0.62		0.47	0.19	0.12	0.77	0.50	0.40	0.41
	0.56	0.47	1.11	0.68	0.72	0.35	0.65		−0.34	−0.41	−0.40	−0.30	−0.27	−0.23	−0.33
	0.55	0.52	0.28	0.53	0.79	1.06	0.62		1.11	1.56	1.21	1.69	1.74	1.34	1.44
	0.10	0.02	0.08	−0.27	−0.17	−0.12	−0.06		0.45	0.02	−0.46	0.29	0.64	0.17	0.19
	0.98	1.31	0.88	1.25	1.19	1.13	1.12		0.74	0.44	0.84	0.87	0.45	0.53	0.65
	0.50	0.47	0.05	0.77	0.36	0.55	0.45		0.55	1.80	0.34	1.04	0.98	0.92	0.94
	0.50	−0.08	0.12	0.06	0.40	0.45	0.24		1.90	1.57	0.96	1.53	1.53	1.68	1.53
	−0.91	−1.19	−0.15	−1.05	−0.53	−0.31	−0.69		1.74	2.39	0.68	1.62	1.85	1.22	1.58
	0.01	0.63	0.65	0.25	0.32	0.20	0.34		0.09	0.26	0.19	−0.23	0.00	0.18	0.08
Mean	0.08	0.11	0.06	0.07	0.12	0.11	0.10		0.82	0.92	0.59	0.95	0.86	0.81	0.82

The main outcome of our Bayesian analysis was that model 3 showed extremely large LRs (see Table 2), indicating that the presence of difference between the PSD contrasts in both groups has high statistical evidence in the EO condition. Of note, this outcome is independent of the prior used. In detail, we created a total of 45 models (five models of nine variations each) and made 225 comparisons (9 variations time 25 comparisons per prior combination). The exact LR naturally differed across each of the variations which can be seen in the Table 2 for the priors Normal (0,1) and Uniform (0,5). The remaining tables can be found in Appendix C. The Bayesian analysis confirms what was illustrated in Figs. 5 and 6: a PSD contrast difference between the groups does exist (contra models 1 and 2) which cannot be further discriminated in separate brain regions (contra models 4 and 5).

**Table 2:** LRs for comparing five models with prior parameters for the PSD contrast: normal (0,1) and prior overall sigma parameter: uniform (0,5).

	Model 5	Model 4	Model 3	Model 2	Model 1
Model 5	1	4.84e−05	8.23e+05	137	2.71e−10
Model 4		1	1.70e+10	2.83e+03	5.60e−06
Model 3			1	1.67e−07	3.29e−16
Model 2				1	1.98e−09
Model 1					1

Highest LR can be found for Model 3, which therefore explains the data best.

For Model 3 the t-score was 5.5, leading to a p-value < 0.001. Table 3 illustrates the prior and posterior densities of Model 3 for each combination of priors. It also shows that the change in power between the sessions differed between both groups (RF in session A and RF in session B). The results are almost identical and show a PSD contrast difference of −0.72 μV^2^/Hz. This number indicates the size of the EMF effect on the magnitude of the EEG power in the alpha band.

**Table 3:** Estimation of the PSD contrast difference according to Model 3.

PSD contrast prior	Sigma prior	PSD contrast group 1 (μV^2^/Hz) (modus and 95% credible interval)	PSD contrast group 2 (μV^2^/Hz) (modus and 95% credible interval)	Sigma (modus and 95% credible interval)	PSD contrast difference (μV^2^/Hz)
Normal (0,1)	Uniform (0,5)	0.0961 (−0.0648; 0.26)	0.8191 (0.6576; 0.98)	0.81 (0.74; 0.90)	−0.723 (−0.948; −0.489)
Normal (0,1)	Gamma (2,2)	0.0956 (−0.0644; 0.26)	0.8196 (0.6580; 0.98)	0.81 (0.73; 0.90)	−0.723 (−0.954; −0.498)
Normal (0,1)	Half-normal (0,2)	0.0958 (−0.0661; 0.257)	0.8193 (0.6610; 0.982)	0.81 (0.73; 0.90)	−0.724 (−0.952; −0.495)
Uniform (−8,8)	Uniform (0,5)	0.0923 (−0.0695; 0.258)	0.8258 (0.6633; 0.987)	0.81 (0.73; 0.90)	−0.733 (−0.962; −0.501)
Uniform (−8,8)	Gamma (2,2)	0.092 (−0.0701; 0.253)	0.826 (0.6651; 0.990)	0.81 (0.73; 0.90)	−0.734 (−0.964; −0.507)
Uniform (−8,8)	Half-normal (0,2)	0.0923 (−0.0706; 0.254)	0.8255 (0.6625; 0.987)	0.81 (0.73; 0.90)	−0.733 (−0.964; −0.502)
Cauchy (0,1)	Uniform (0,5)	0.0964 (−0.0647; 0.259)	0.8166 (0.6526; 0.977)	0.81 (0.73; 0.90)	−0.72 (−0.949; −0.491)
Cauchy (0,1)	Gamma (2,2)	0.0966 (−0.0654; 0.258)	0.8168 (0.6554; 0.979)	0.81 (0.73; 0.90)	−0.72 (−0.948; −0.492)
Cauchy (0,1)	Half-normal (0,2)	0.0962 (−0.0676; 0.257)	0.8168 (0.6567; 0.981)	0.81 (0.73; 0.90)	−0.721 (−0.949; −0.492)

## Discussion

In this work, we assessed the effects of EMF on brain EEG while controlling for environmental and experimental confounds that are commonly present in (older) EMF literature. We report that our experimental space is almost free of EMF that is normally present in laboratory spaces, that our EEG equipment itself is unaffected in any significant ways by the EMF that is used in this study, and that EMF has an effect on the brain that is characterized by a subtle increase in EEG power in a band between 6 and 10 Hz.

The measurements of the EMF power densities indicated that the background radiation at the location of the volunteer was 3 orders of magnitude smaller than typical values in the public space. In this way, the outcome of our measurements could not be attributed to the presence of EMF levels already present in normal working spaces. Power density level measured inside the area between the antennas were in the same order of magnitude of the power density close to a mobile phone during a phone call as reported elsewhere in the literature^24^, indicating that our experiment can be considered as a representative for a situation in which one talks with a mobile device close to the ear.

In this paper we dealt with the criticism that was raised in the past, that the application of pulsed RF would lead to artefacts in the EEG and that the spectral energy of these spikes can be folded into the EEG recording, thus erroneously suggesting an EMF effect in the brain^18^. In the phantom experiment we showed that this is not the case. At 217 Hz, a signal is present in the RF on case, but it does not change the signal in the biological relevant range in any significant manner. Previously, others have argued that the absence of an EMF effect in the eyes closed condition also contradicts the presence of electrical interference^25^, since one would expect the latter to be present in both conditions. Furthermore, the utilization of active electrodes in our setup is anticipated to effectively mitigate artifacts commonly introduced into the leads between the electrode and the EEG amplifier.

In our paper we replicated the finding that EMF effects are difficult to capture in the eyes closed state^11,25^. Apparently, the brain is more sensitive to EMF when the eyes are open. However, it is unlikely that the effect of EMF is entirely absent during eyes closed (EC) conditions. This is because alpha power is stronger during EC, making it more challenging to detect subtle modulations arising from EMF exposure.

The experimental design for investigating the relationship between EMF and brain function is not straightforward. To effectively explore subtle EMF effects while minimizing bias, experiments employing a double-blind, counterbalanced, crossover design are considered most suitable. However, one can debate the optimal approach for counterbalancing. In our study, we chose to expose participants to both sham and RF conditions within a single session in a counterbalanced fashion. Alternatively, some studies have implemented counterbalancing over two or more sessions to avoid carryover effects^11,25^. But this approach may come at the cost of introducing additional variation between the sham and RF on conditions, making it more difficult to detect the actual EMF effect. Indeed, in quite some studies with a counterbalanced design no effects in the alpha band were reported^11,12,26^.

We chose to perform both sham and RF measurement in one single session. One may criticize this design for its sensitivity to carryover EMF effects. It has been suggested that they may persist for approximately half an hour^27^, although limited data is available to make any conclusive claims^16^. In our investigation, we did observe that the increase in alpha power was predominantly present in the RF-on condition following the RF-off condition. In the cases where we started with RF on, the PSD contrast was close to zero (see datapoints in black in Fig. 6). This could mean that the EMF effect remained present during session B. Alternatively, our data could be interpreted by assuming the existence of a non-EMF order effect, which leads to a gradual increase in alpha power over time in both groups. In the extreme case that there is no carry over, i.e. the EMF has no transient effect and only an order effect is present one would need to take into account that the reported difference in PSD contrast contains twice the EMF effect. However, even with this consideration, our conclusion regarding the presence of an EMF effect in our data remains unchanged. Another reason for choosing a counterbalanced design where effects are assessed within subjects, is that the between-subject variability (which tends to be higher than the within-subject variability) is controlled for.

Given the above considerations we suggest that our approach to counterbalancing yields robust results and illustrates that the benefit of measuring on multiple days is not self-evident. More consensus on the setup of an EEG experiment is highly desirable in the light of the 5G protocols that are currently deployed. The current 5G protocols do still use similar carrier frequencies as 2G but differ substantially in terms of the carrier wave modulation used. The latter may lead to a different effect on brain function.

The effect that we found in the alpha band is not straightforward to interpret. Alpha waves are commonly observed in healthy adults who are awake but at rest with their eyes closed. However, these waves decrease during sleep or when an individual is engaged in a focused task^28^. The alpha rhythm is believed to indicate a reduction in cortical activity during relaxation and is associated with cognitive inhibition and visual relaxation^29^. It is important to note that modifications in alpha wave activity do not necessarily lead to the development of pathological conditions. Nevertheless, it is true that certain neurological disorders are accompanied by alterations in EEG patterns, and that the sensitivity to changes induced by electromagnetic fields (EMF) may vary among individuals.

In conclusion, our study provides compelling statistical evidence for the occurrence of increased alpha activity during EMF exposure. By carefully addressing confounding variables that posed challenges in earlier studies, we have effectively demonstrated that the observed effect is highly improbable to be an artefact. Moving forward, our research protocol holds promise for investigating the impact of other transmission protocols, such as 5G and 6G, as well as enabling a more comprehensive examination of dose–effect relationships.

### Supplementary Information

Supplementary Information.

## Data Availability

The datasets generated during and/or analysed during the current study are available from the corresponding author on reasonable request.

## Abbreviations

(2,3,4,5)-G: (2,3,4,5)th-generation
EC: Eyes closed
EEG: Electroencephalogram
EMF: Electromagnetic field
EO: Eyes open
GSM: Global system for mobile communications
ICA: Individual component analysis
MP: Mobile phone
PSD: Power spectral density
RF on: Radiofrequency on
RF off: Radiofrequency off
SCPI: Standard commands for programmable instruments
