# Supplementary material for: Effects of mobile phone electromagnetic fields on brain waves in healthy volunteers
Source: Sci Rep. 2023 Dec 8;13:21758. doi: 10.1038/s41598-023-48561-z (PMC10709380; doi:10.1038/s41598-023-48561-z)
Supplement: Supplementary file 1 — Supplementary Information. [file 41598_2023_48561_MOESM1_ESM.docx]

**Supplementary Material**

This document contains additional sections belonging to the main manuscript “Effects of mobile phone electromagnetic fields on brain waves in healthy volunteers”.

**Appendix A: ICA Removal**

After Individual Component analysis de-mixed the EEG signal into different components, they automatically rated as to belonging to brain, muscle, line noise, eyeblink with a percentage score. We visually inspected properties of the ICA components, which were their timeseries, power spectra, classification scores and topographical distribution. Based on these, we classified components as being artifact and non-artifact. The average number of rejected components across all participants was 22. With data of some participants being more noisy than others, the standard deviation of removed components was 4. This leaves on average 40 components (+/-4) to reconstruct the EEG data. Our general guidelines for rejection were to look primarily at the rating scores. If a rating score for eyeblink, muscle or line noise was > 90%, we marked the component for removal. We did not mark/classify components that have had a brain score of > 5%, even if based on the other scores, it should be marked for removal.

**Appendix B: Single-subject analysis of EEG data**

In this section, we show the PSD estimates of session A and session B for each individual separately. Each figure contains the PSD estimates and their respective standard deviations over all 4-second trials (See Methods section and Figure 2B in the main paper for analysis details). The figures are subdivided into protocols (Eyes Closed followed by Eyes Open), and by group (Group 1 receiving RF in session A; Group 2 receiving RF in session B).

EC, Group 1 (RF in session A)


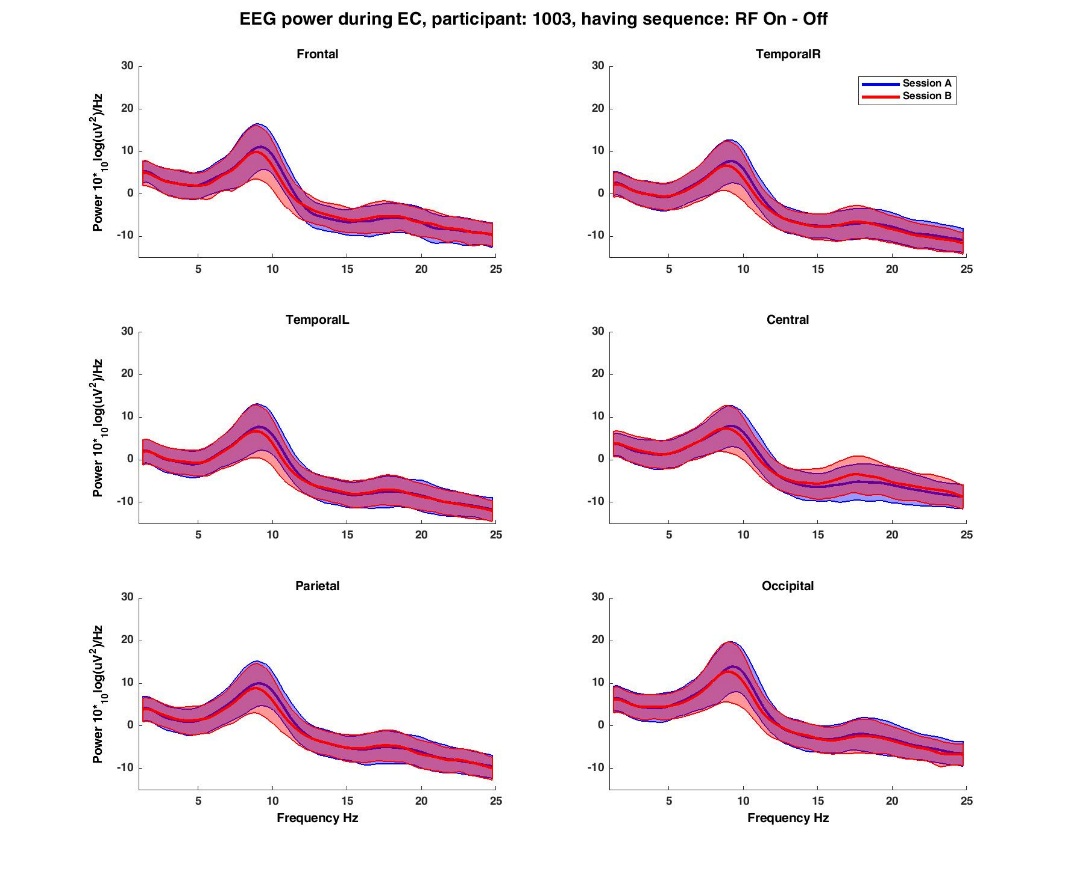


Figure S1. PSDs of participant 1003 for six brain regions in EC condition.


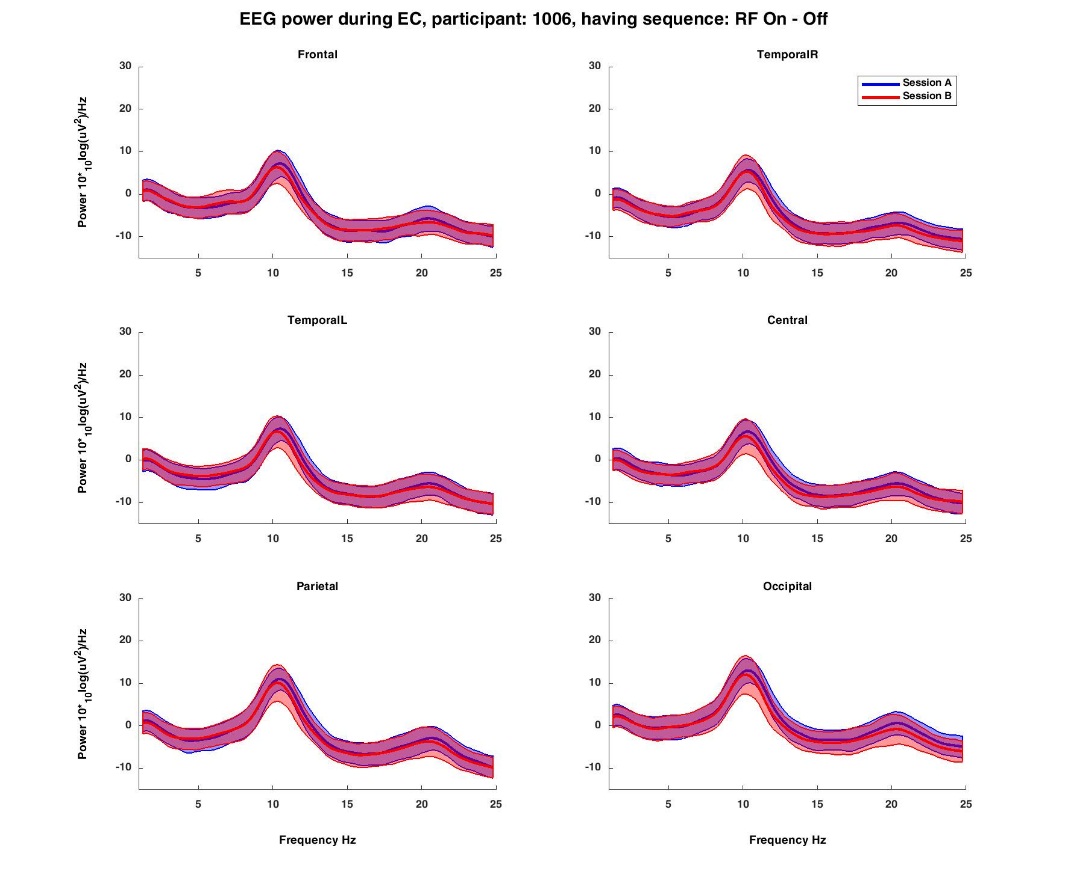


Figure S2. PSDs of participant 1006 for six brain regions in EC condition.


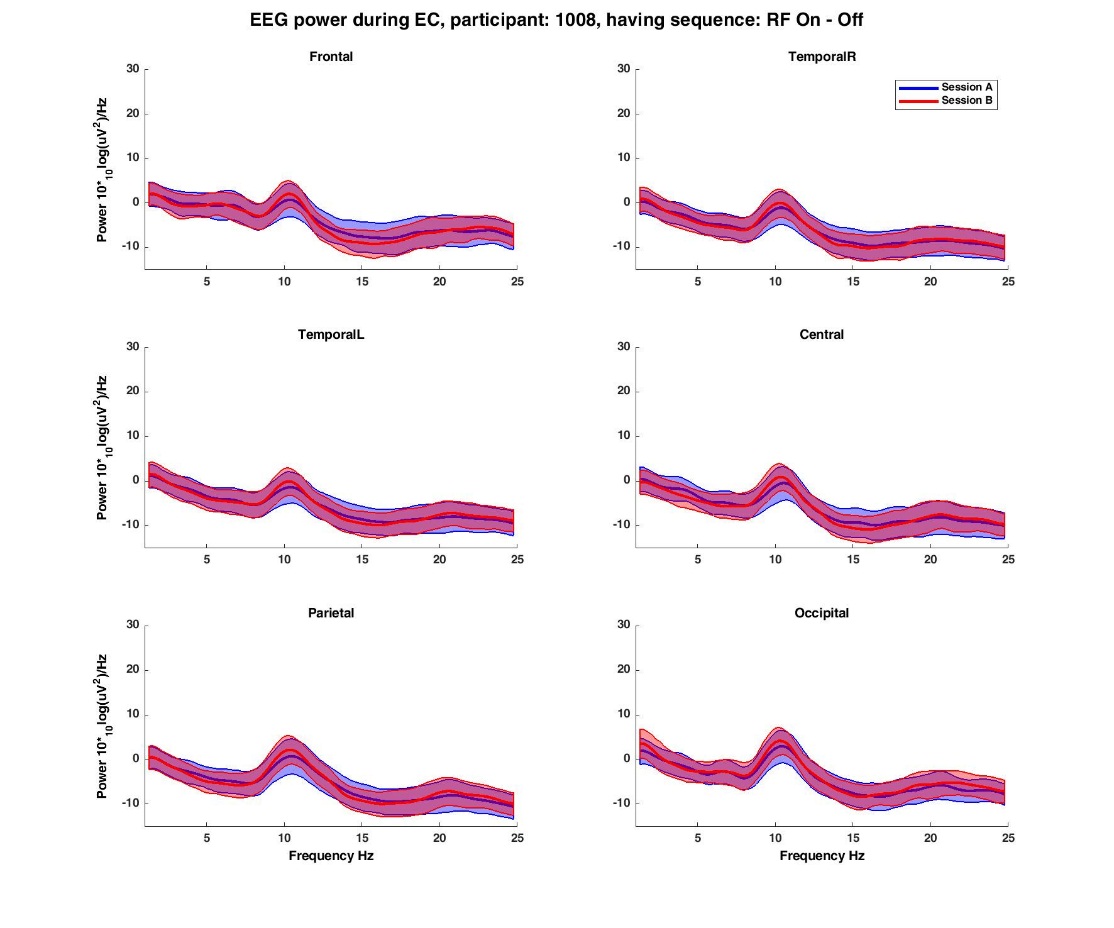


Figure S3. PSDs of participant 1008 for six brain regions in EC condition.


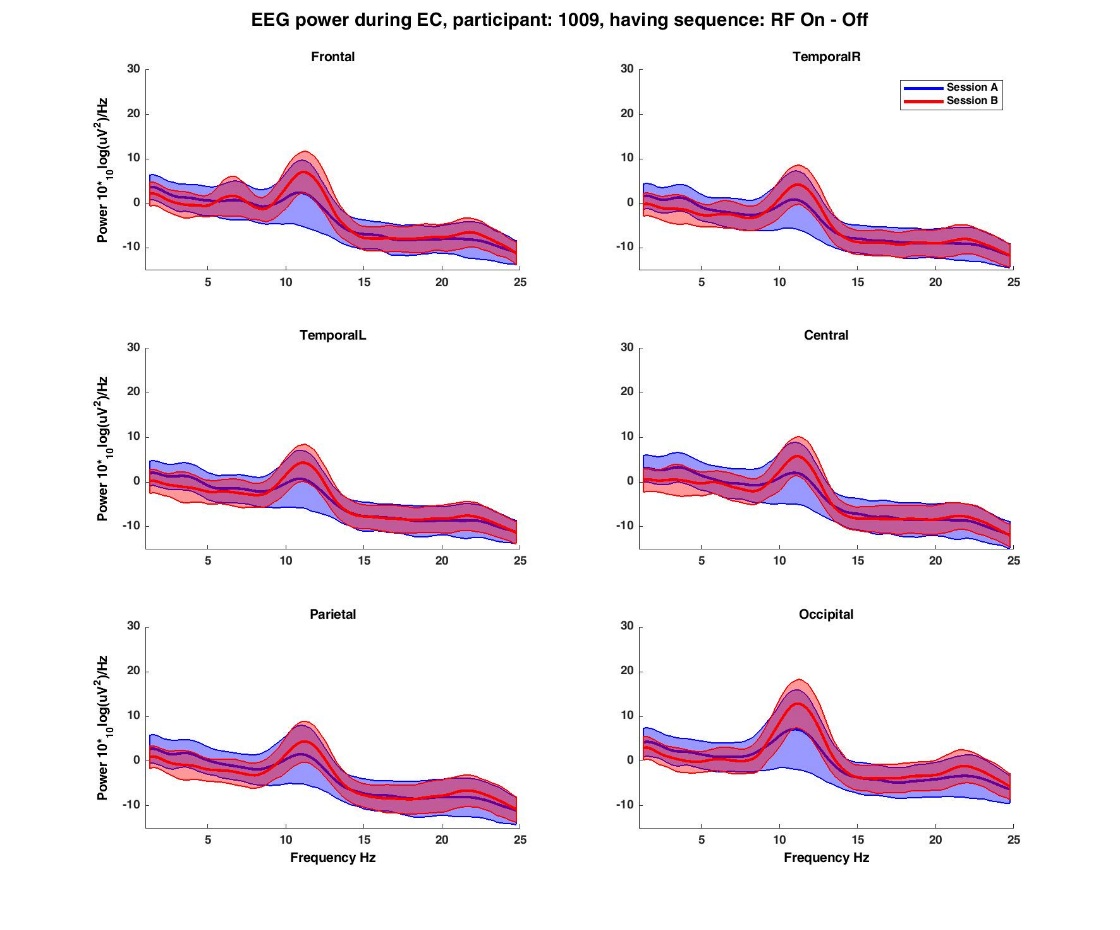


Figure S4. PSDs of participant 1009 for six brain regions in EC condition.


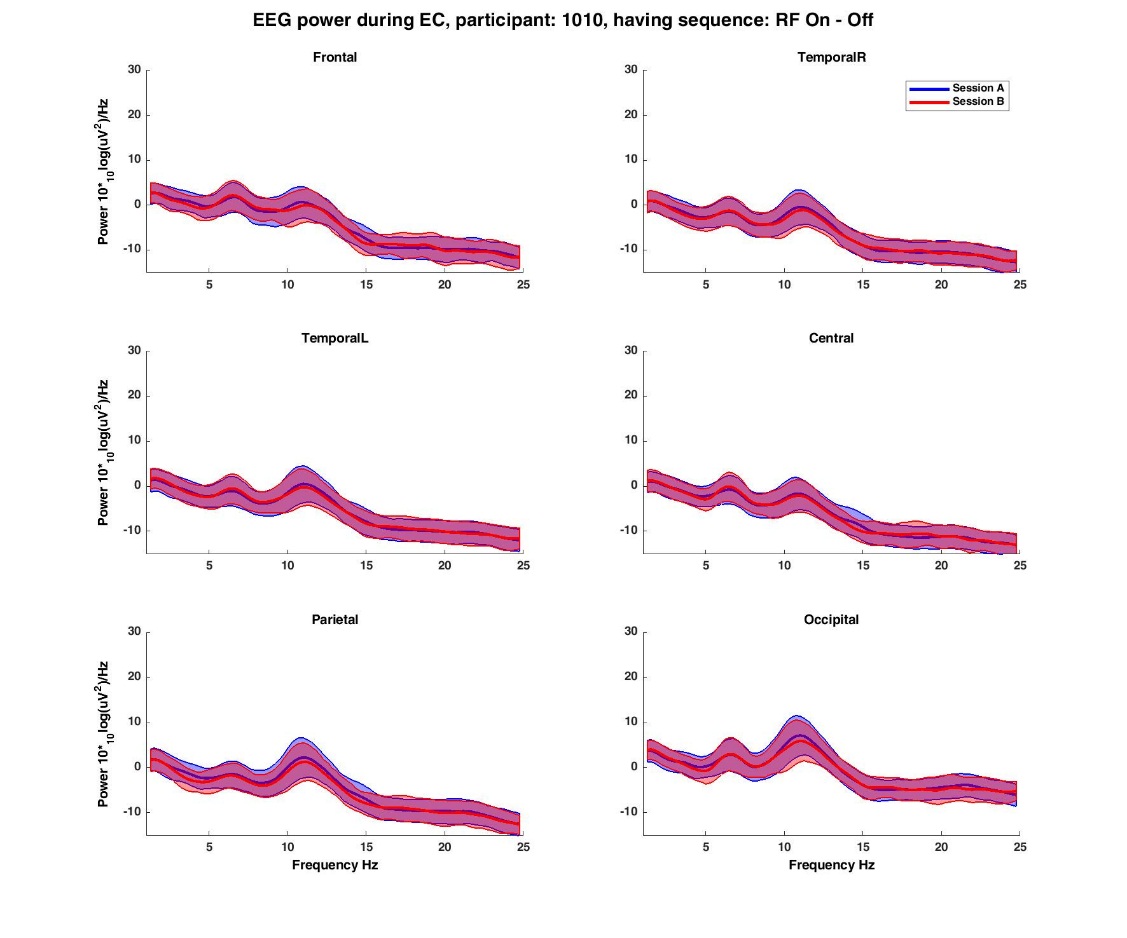


Figure S5. PSDs of participant 1010 for six brain regions in EC condition.


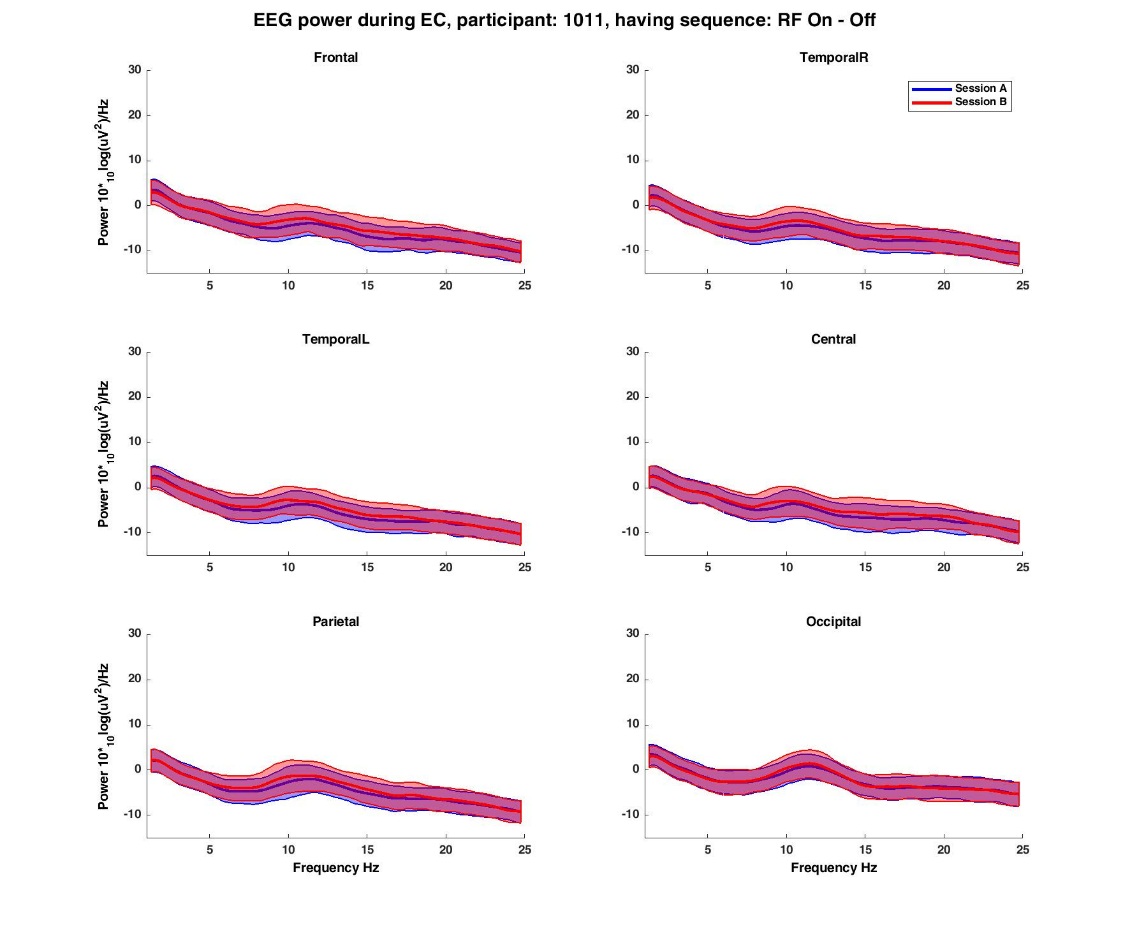


Figure S6. PSDs of participant 1011 for six brain regions in EC condition.


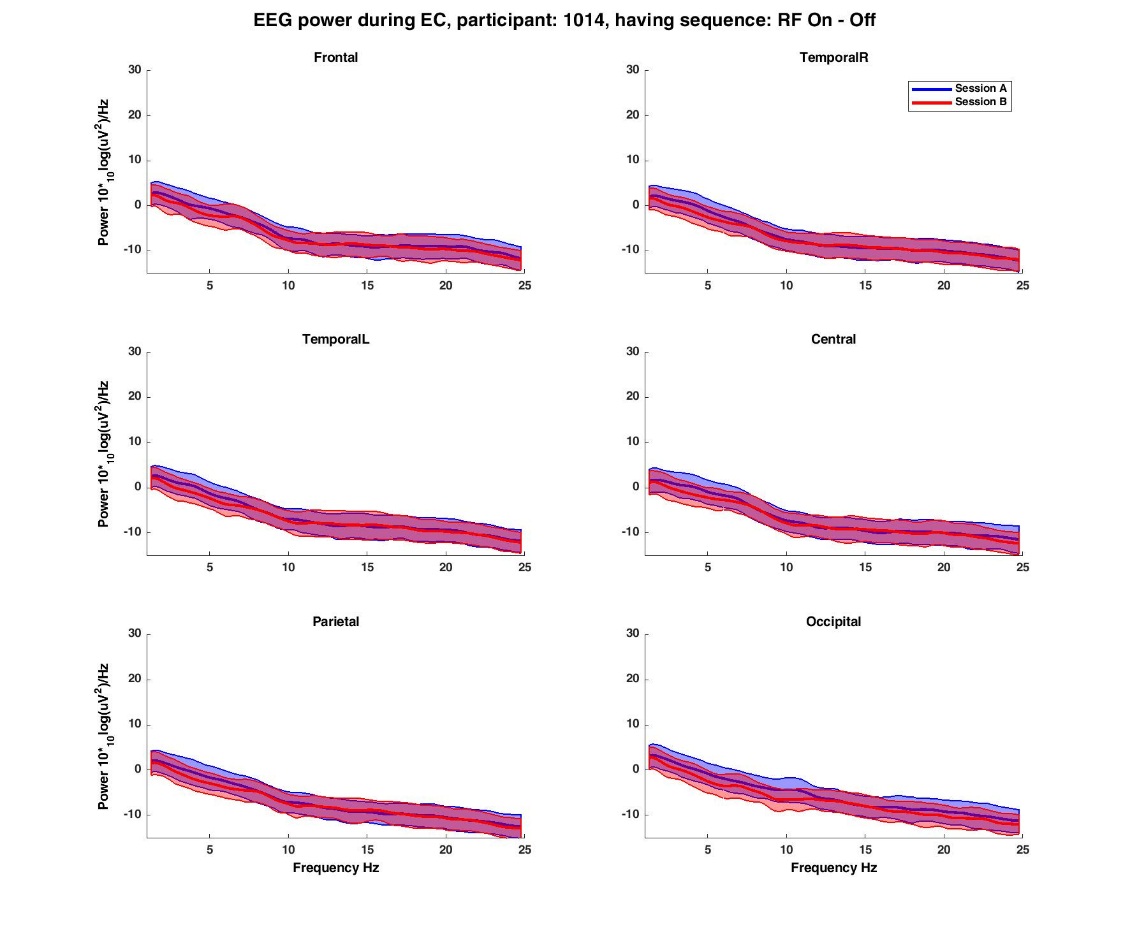


Figure S7. PSDs of participant 1014 for six brain regions in EC condition.


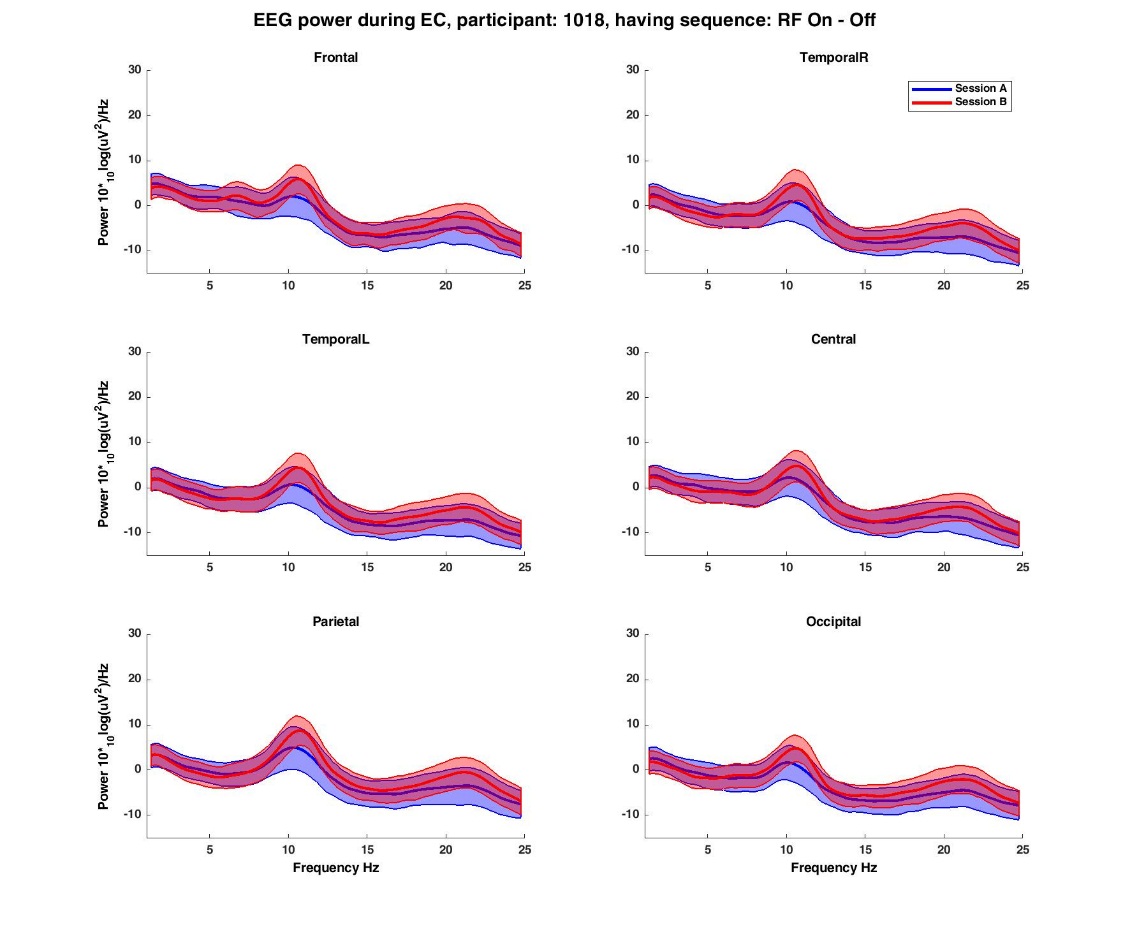


Figure S8. PSDs of participant 1018 for six brain regions in EC condition.


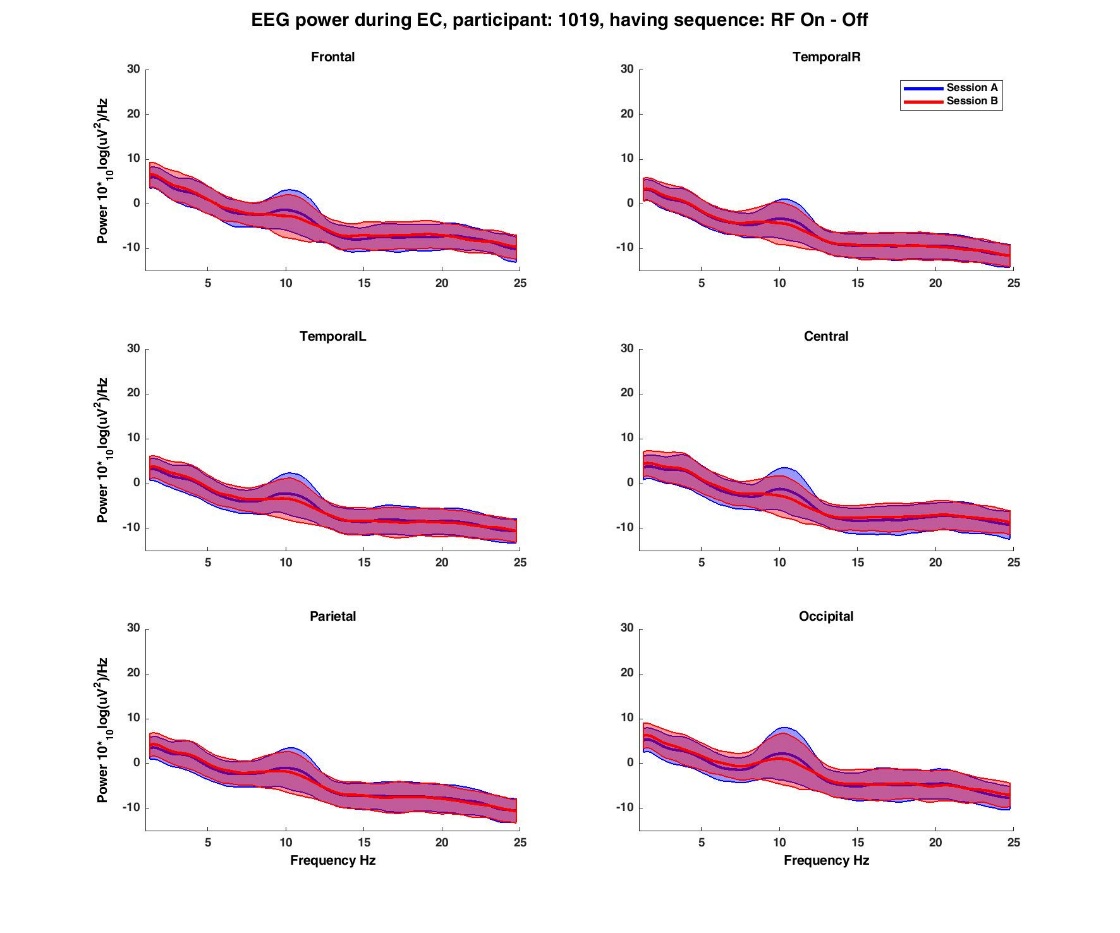


Figure S9. PSDs of participant 1019 for six brain regions in EC condition.


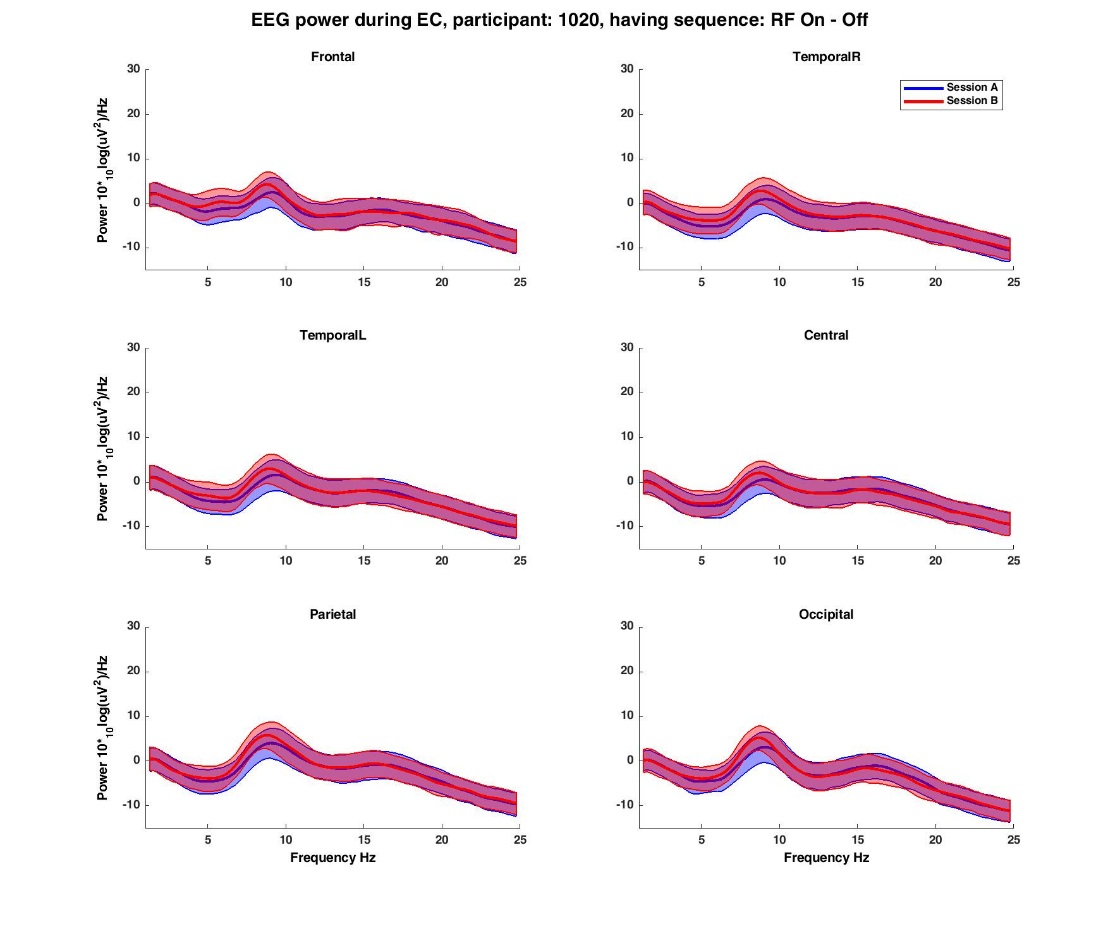


Figure S10. PSDs of participant 1020 for six brain regions in EC condition.


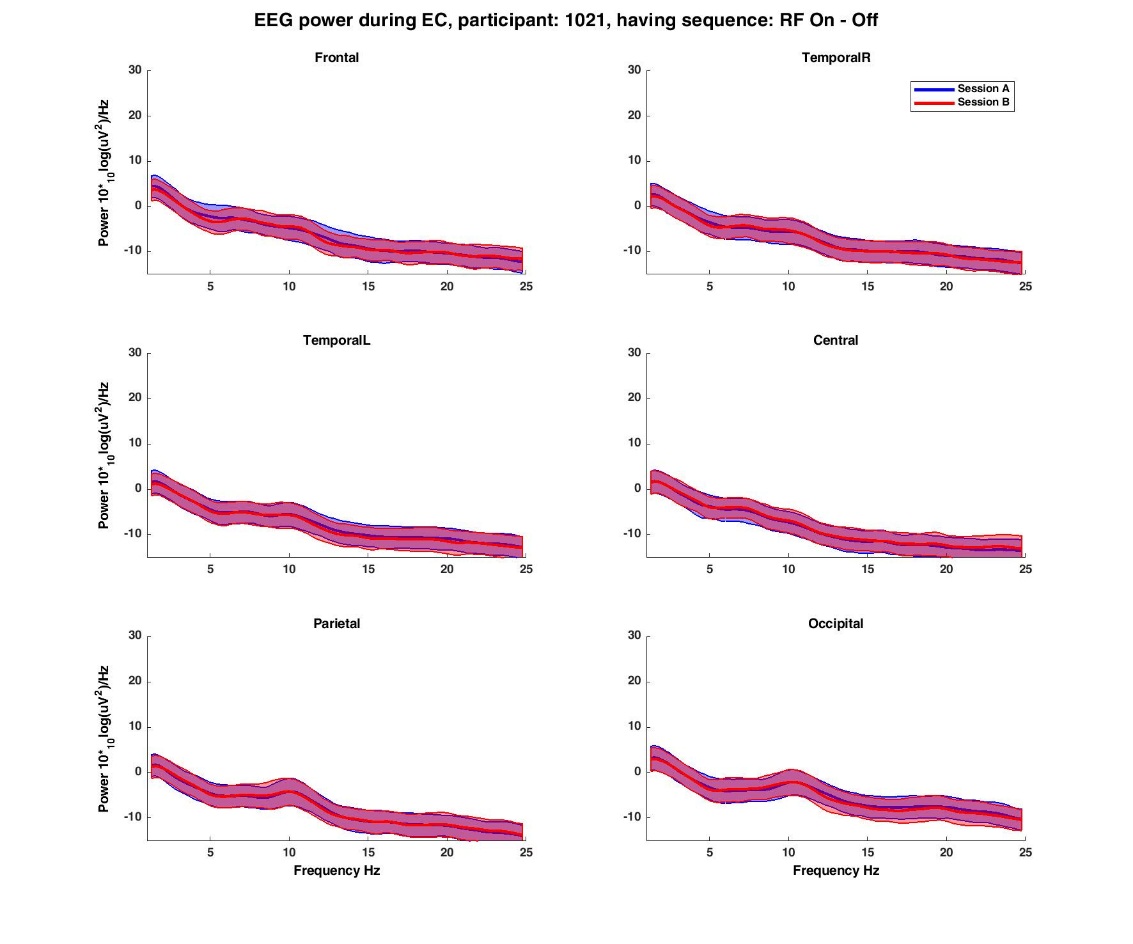


Figure S11. PSDs of participant 1021 for six brain regions in EC condition.


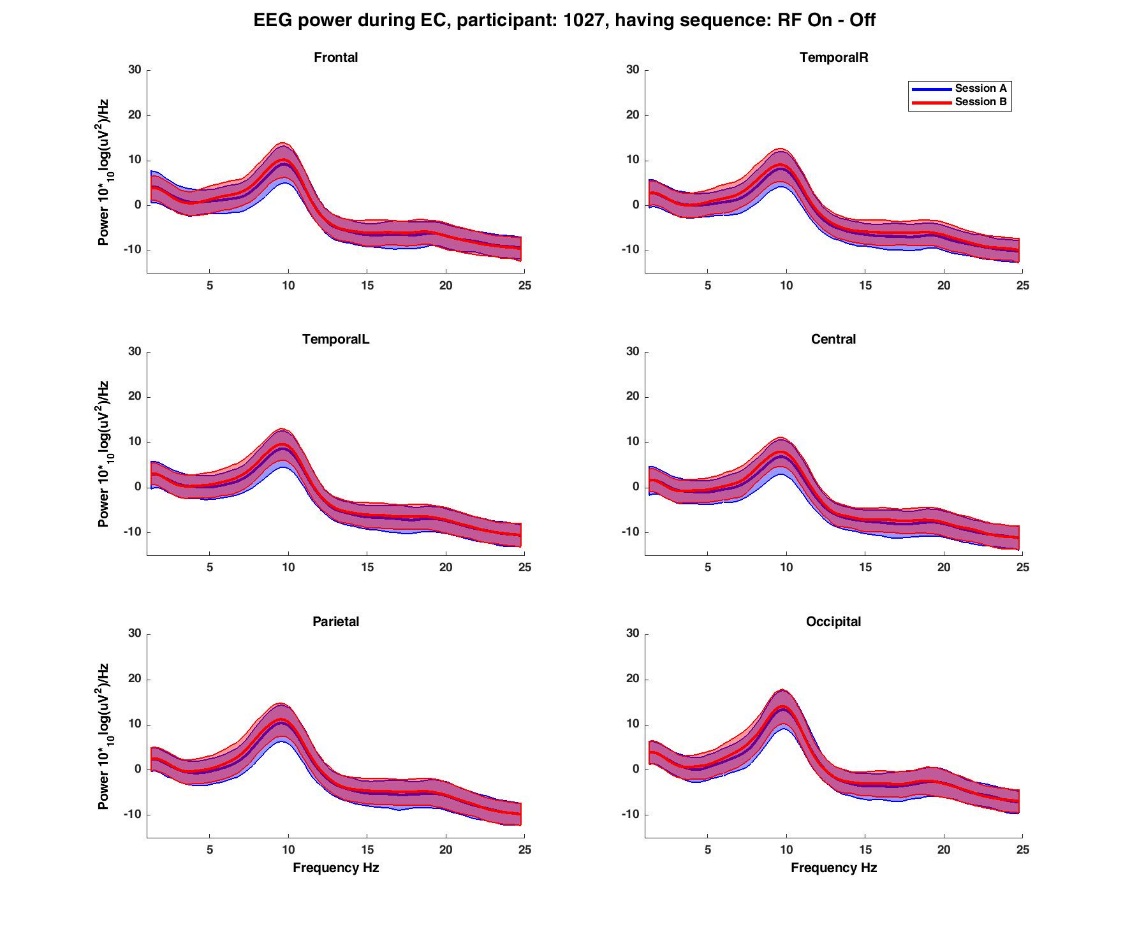


Figure S12. PSDs of participant 1027 for six brain regions in EC condition.


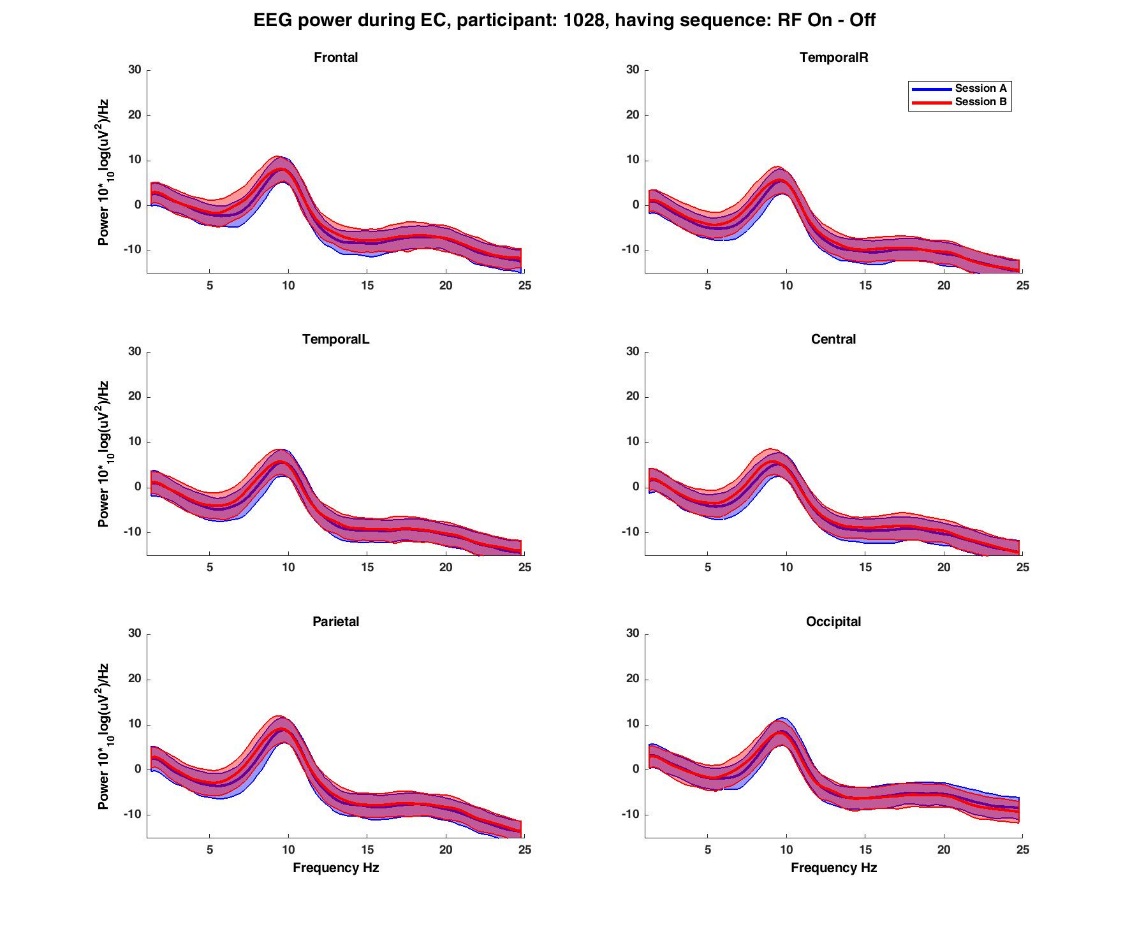


Figure S13. PSDs of participant 1028 for six brain regions in EC condition.


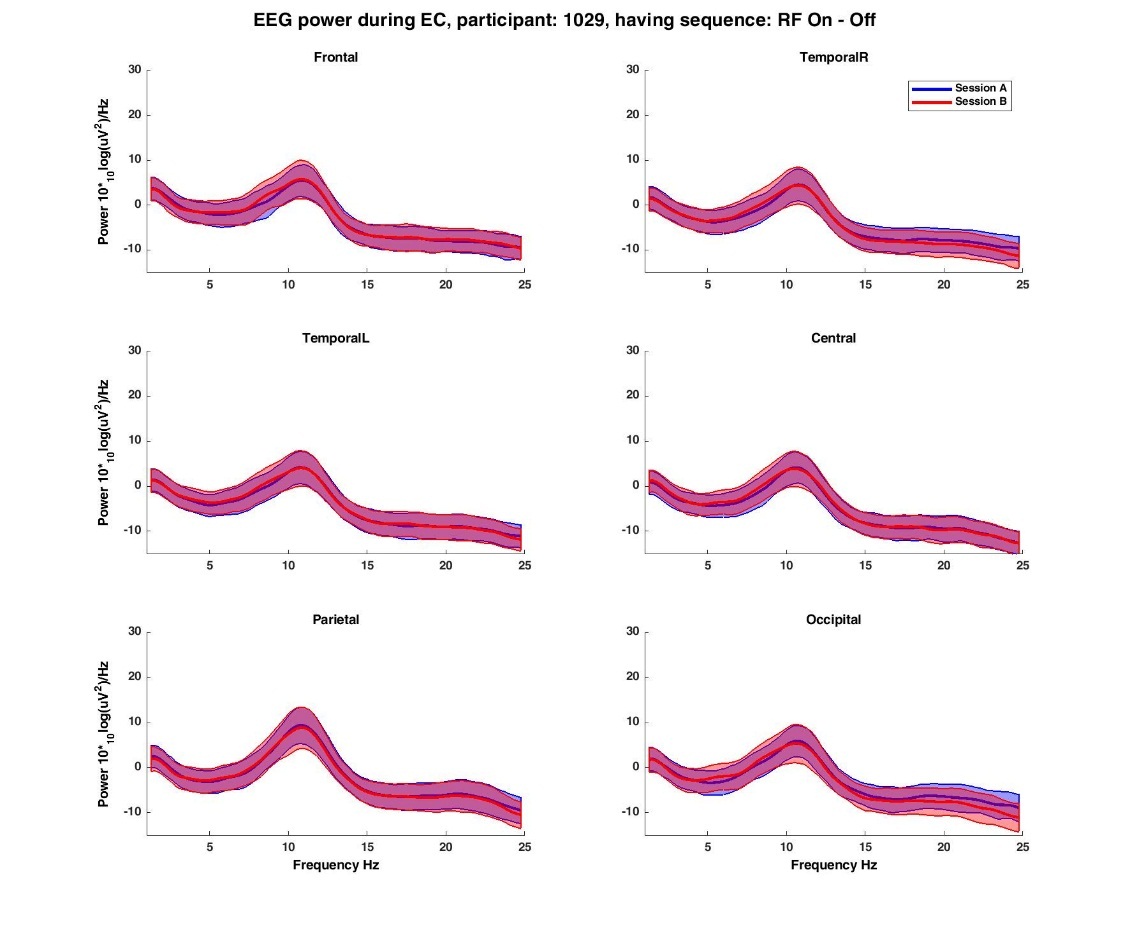


Figure S14. PSDs of participant 1029 for six brain regions in EC condition.


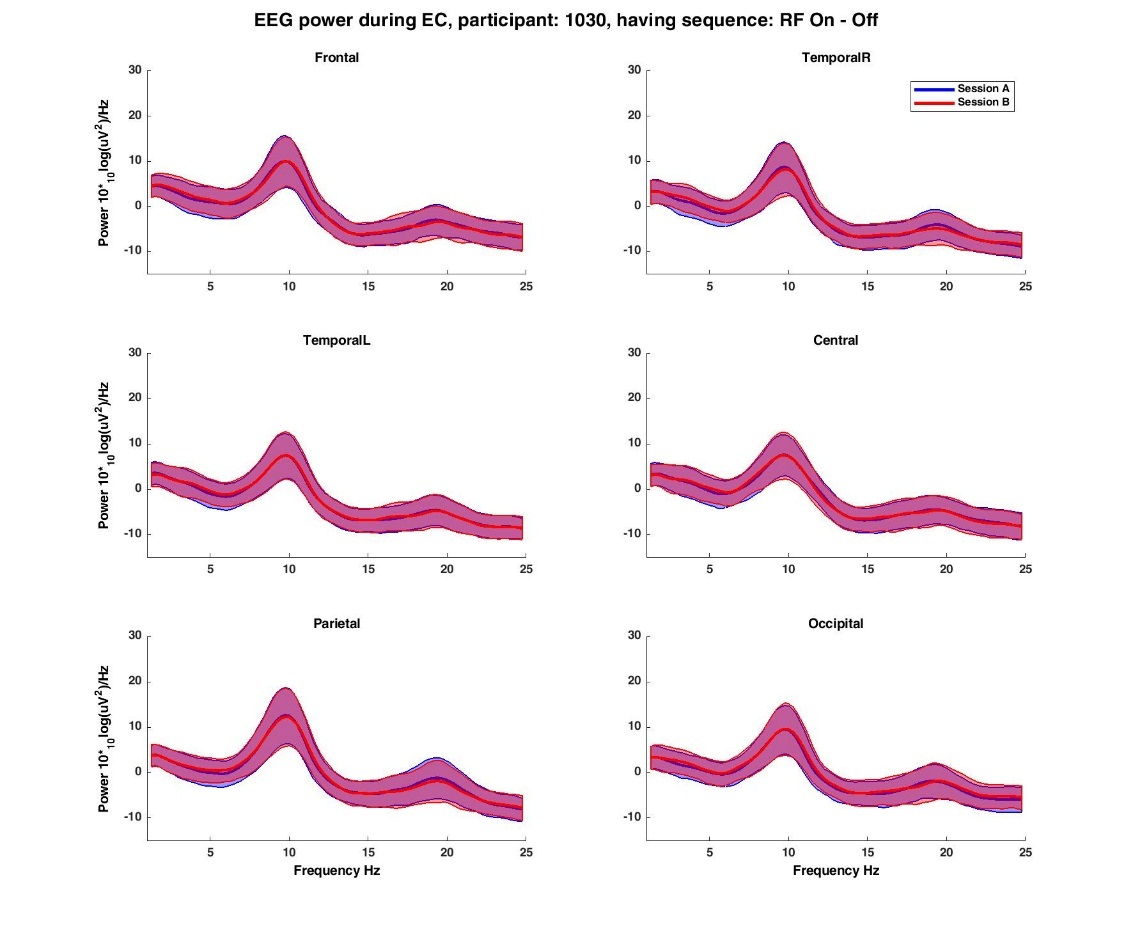


Figure S15. PSDs of participant 1030 for six brain regions in EC condition.


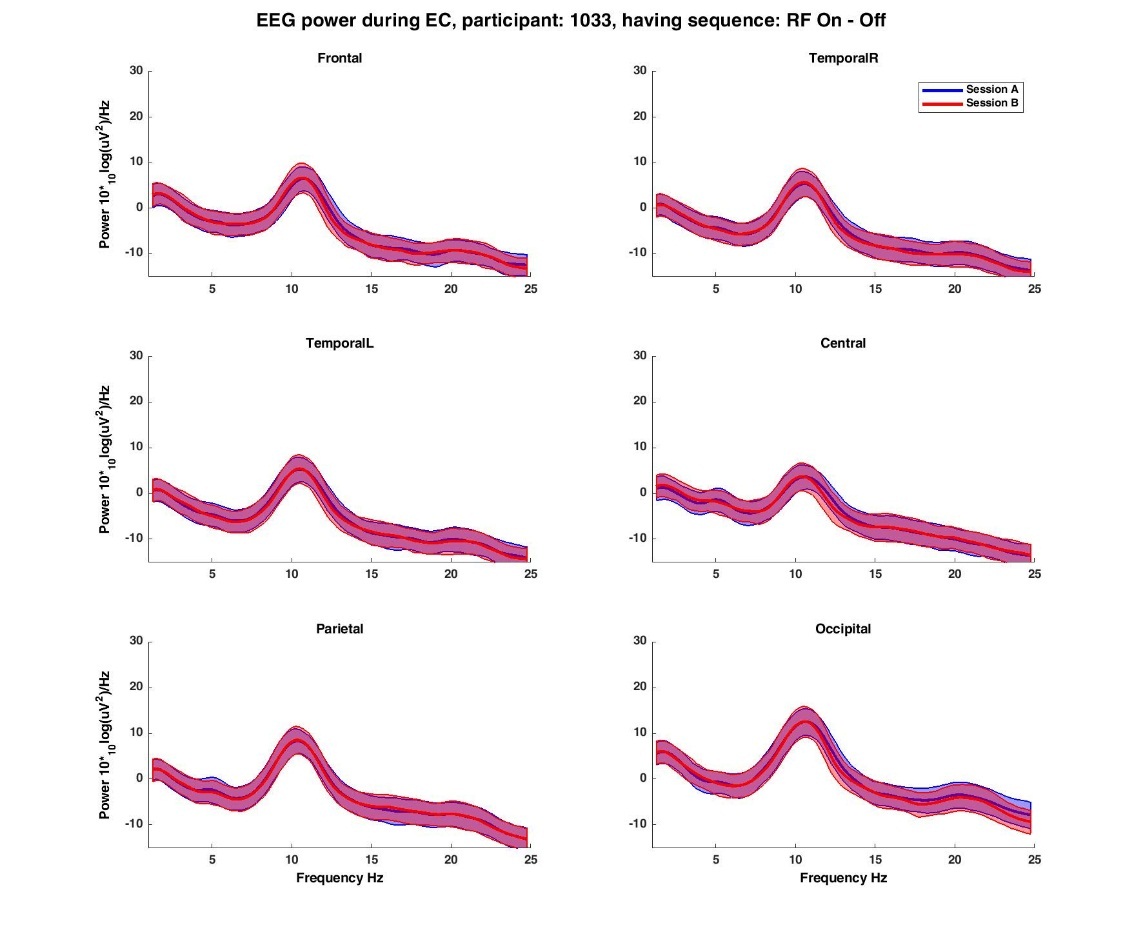


Figure S16. PSDs of participant 1030 for six brain regions in EC condition.

EC, Group 2 (RF in session A)


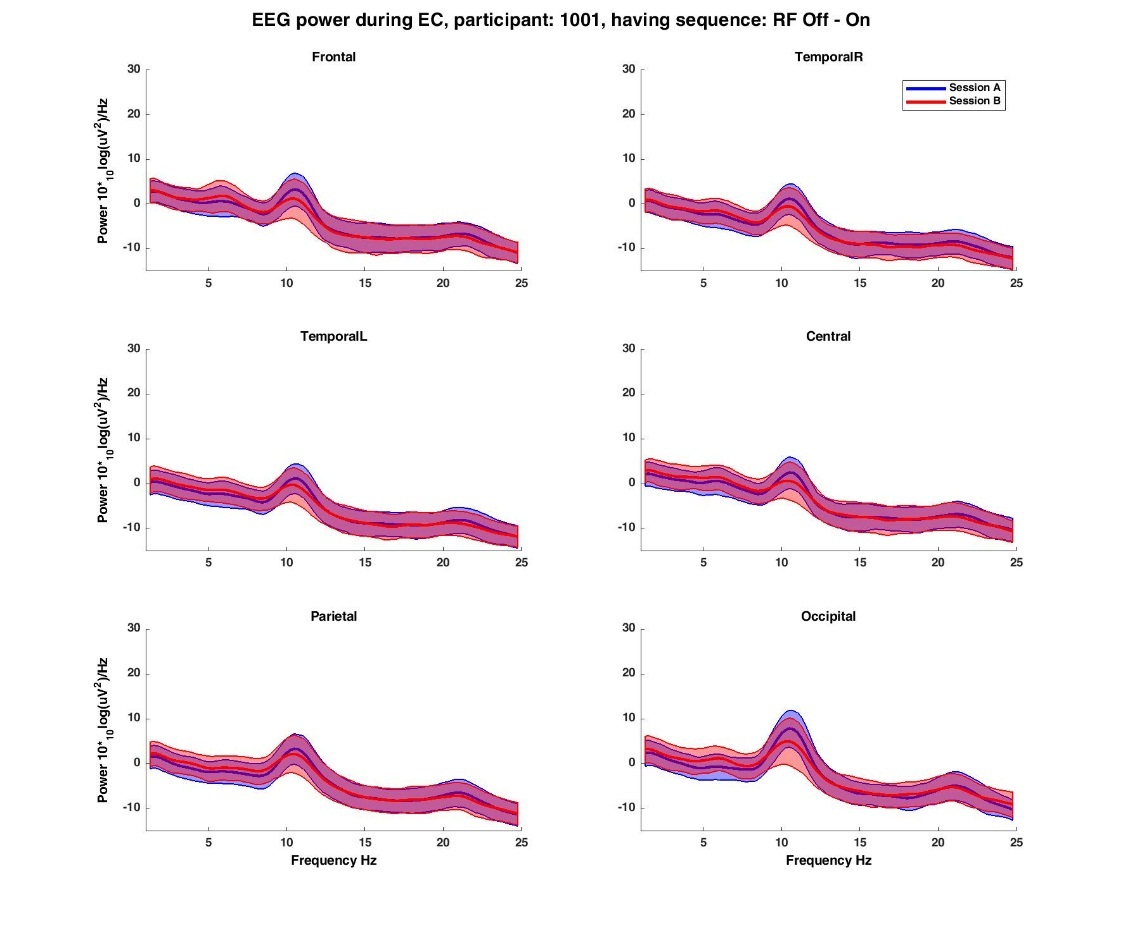


Figure S17. PSDs of participant 1001 for six brain regions in EC condition.


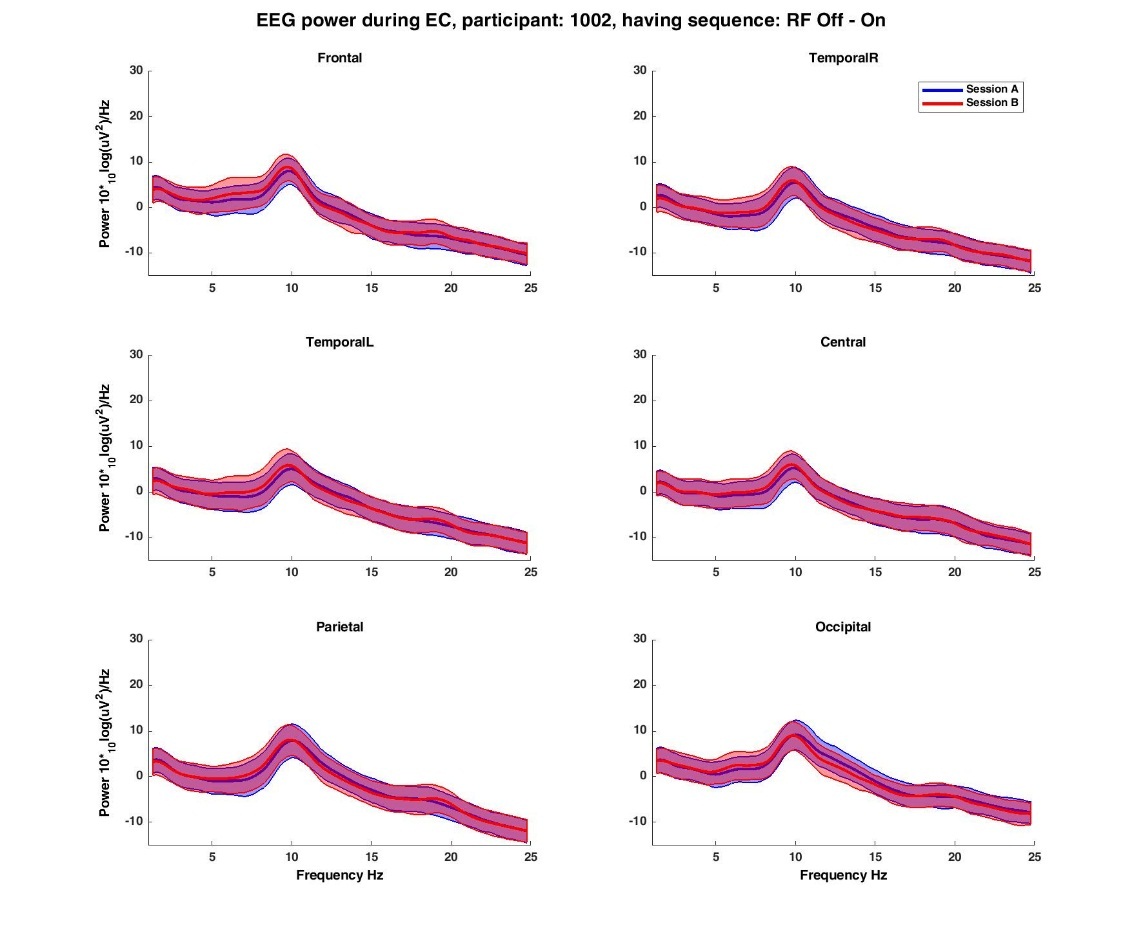


Figure S18. PSDs of participant 1002 for six brain regions in EC condition.


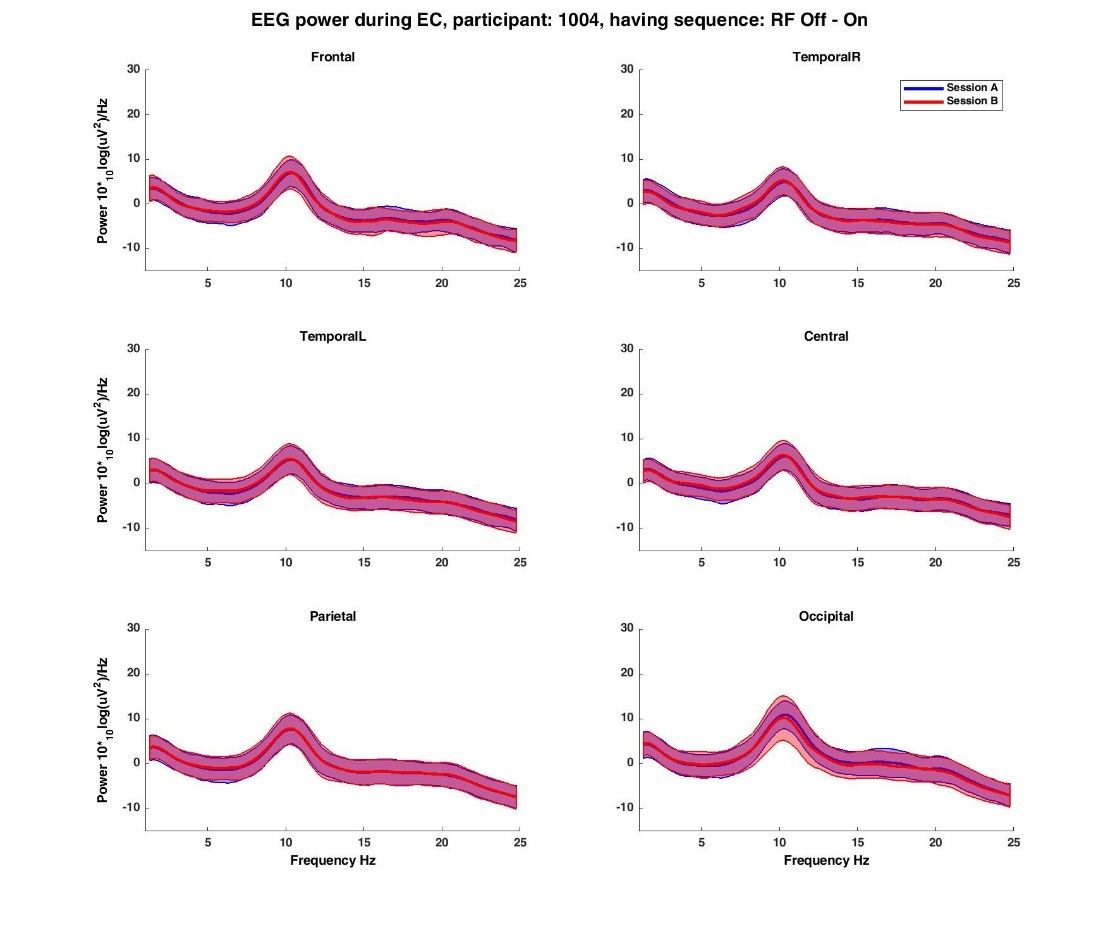


Figure S19. PSDs of participant 1004 for six brain regions in EC condition.


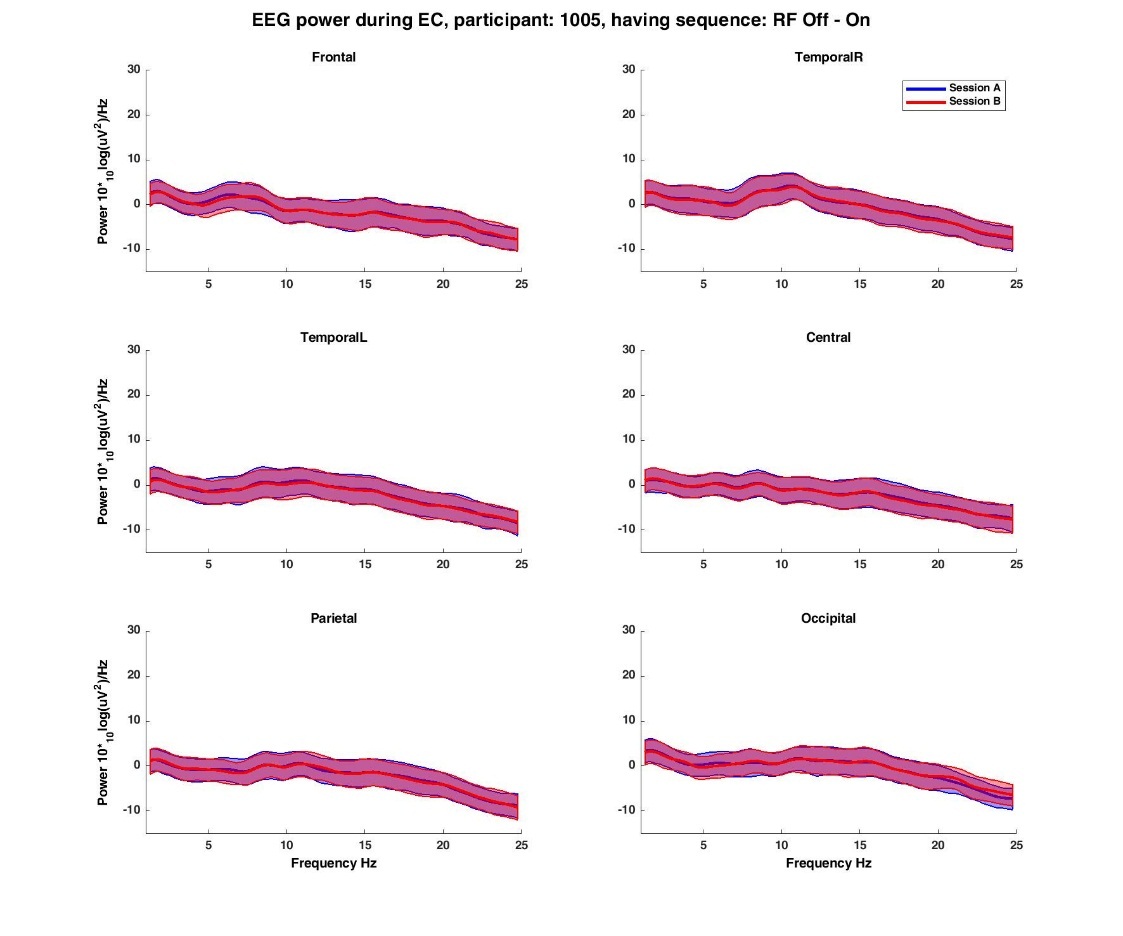


Figure S20. PSDs of participant 1005 for six brain regions in EC condition.


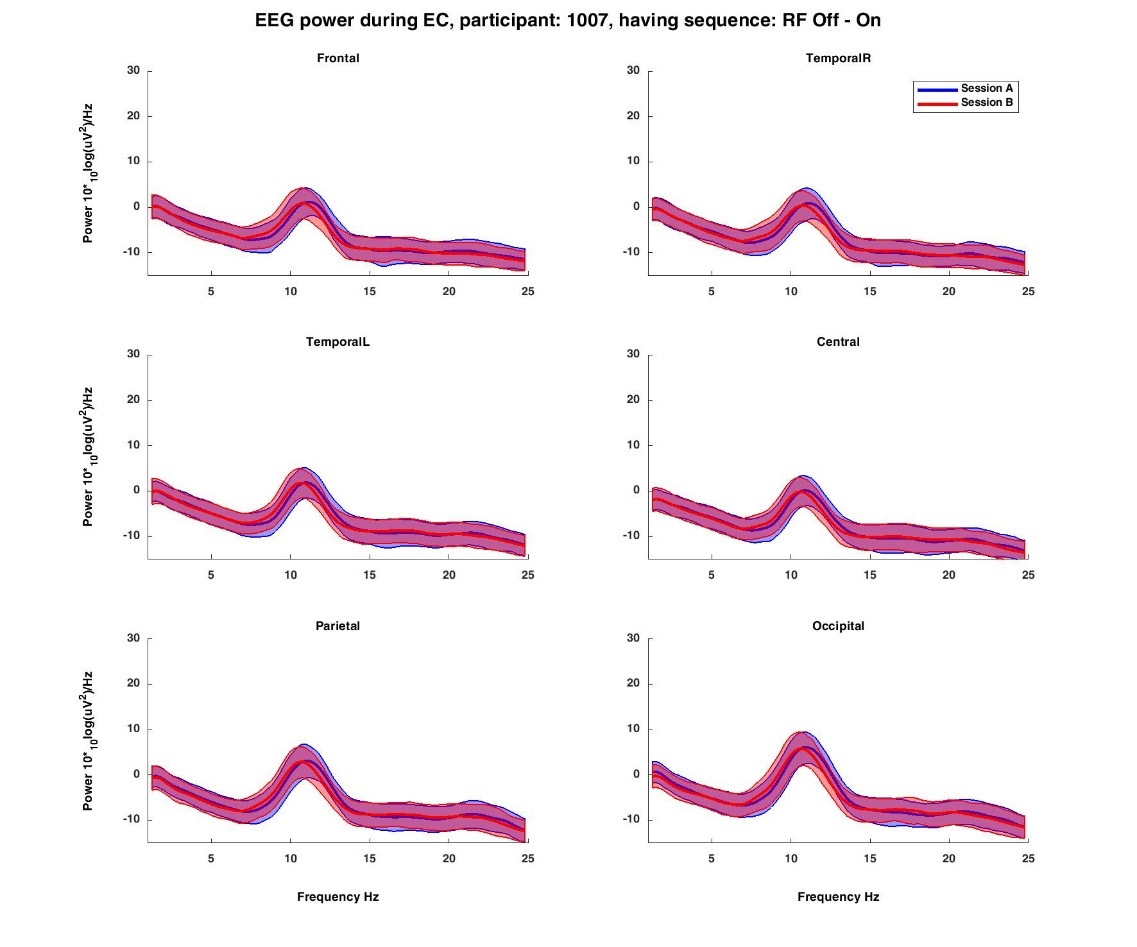


Figure S21. PSDs of participant 1007 for six brain regions in EC condition.


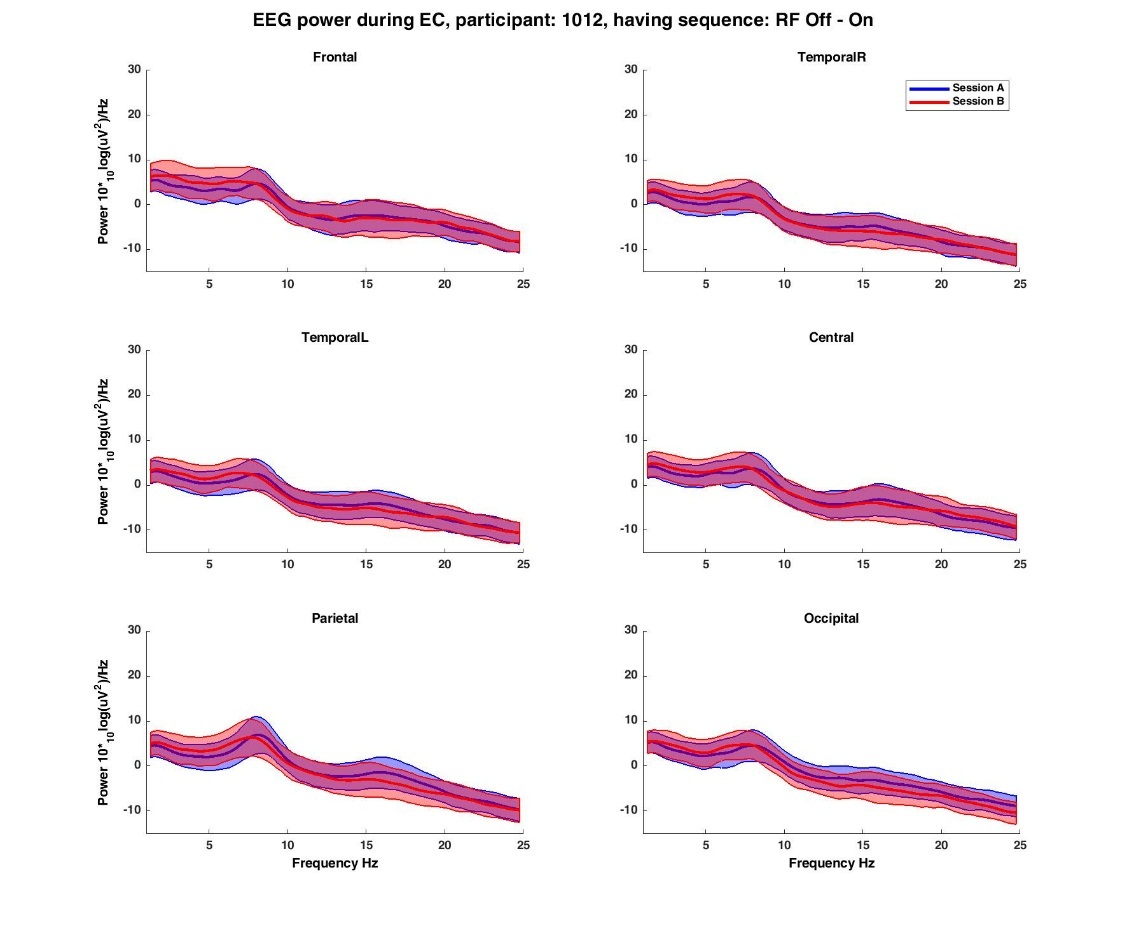


Figure S22. PSDs of participant 1012 for six brain regions in EC condition.


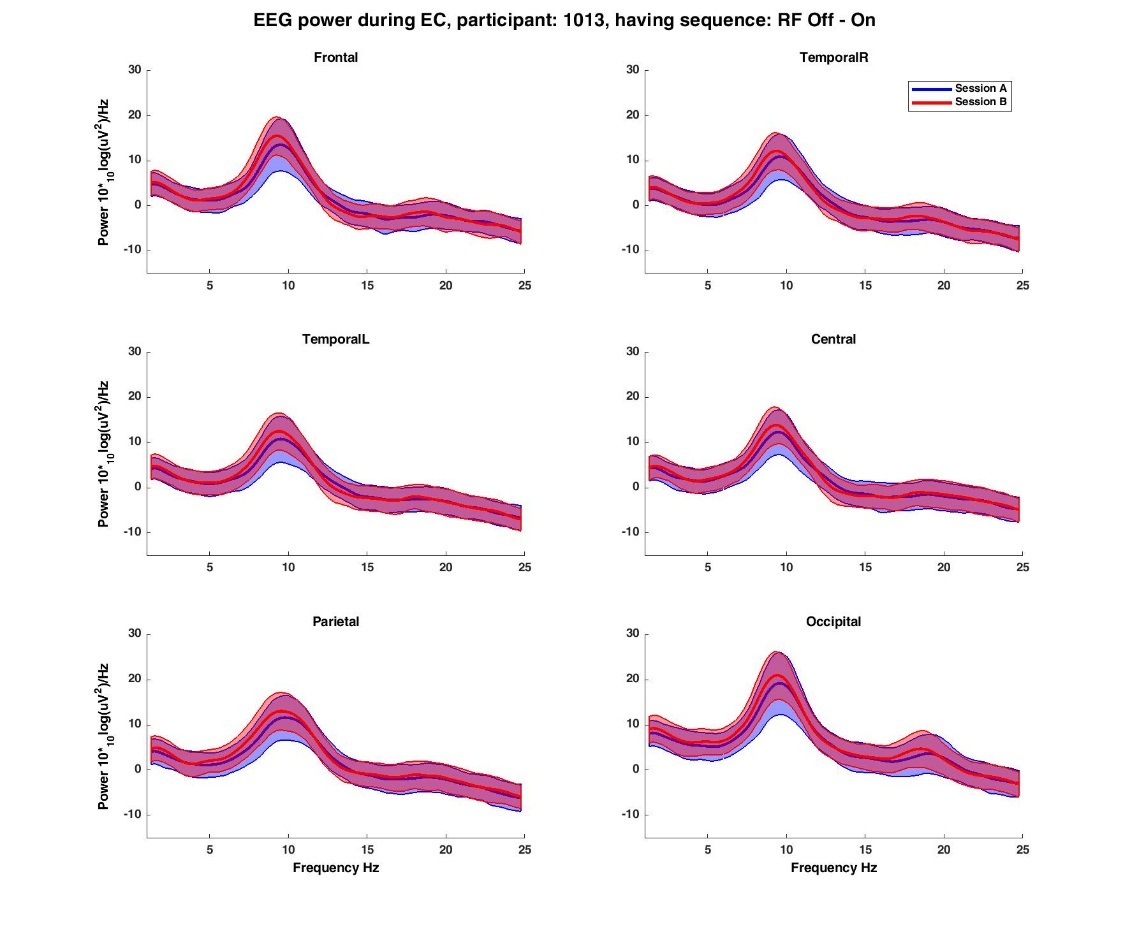


Figure S23. PSDs of participant 1013 for six brain regions in EC condition.


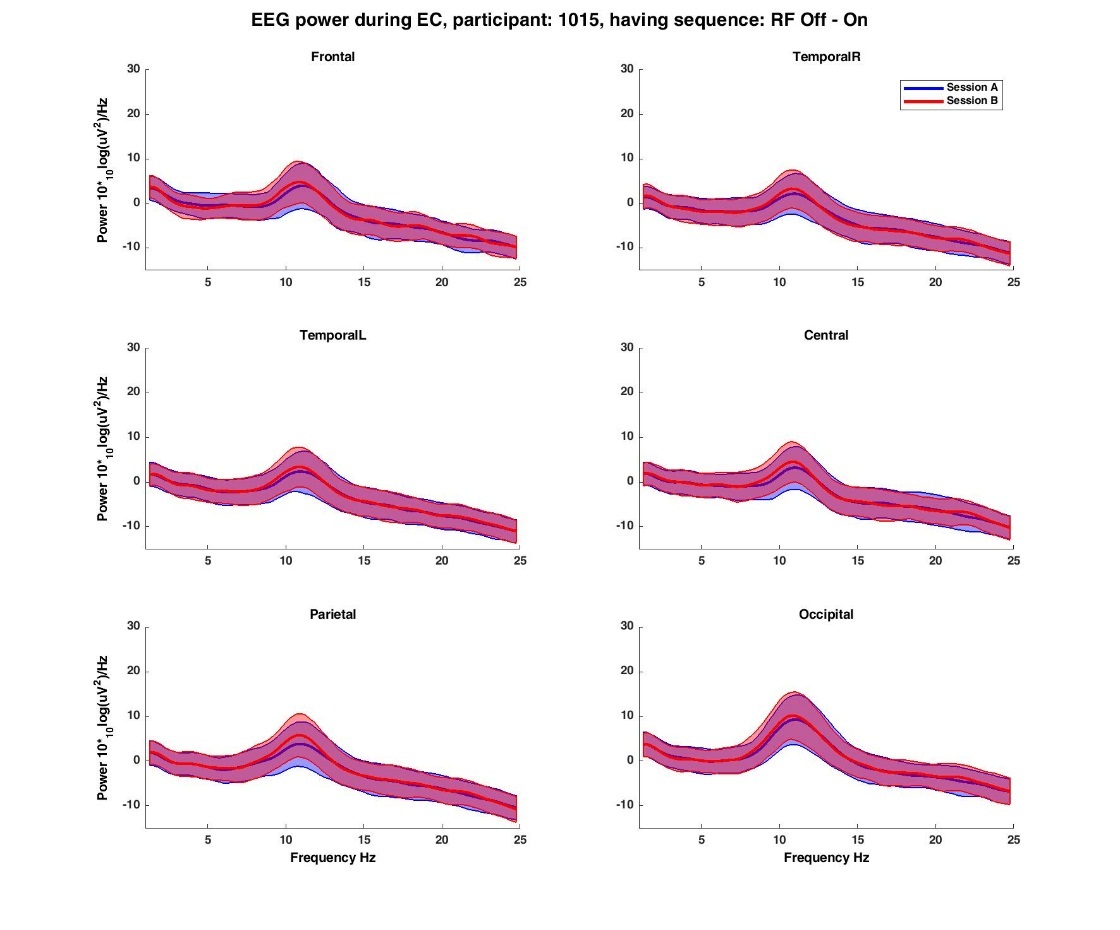


Figure S24. PSDs of participant 1015 for six brain regions in EC condition.


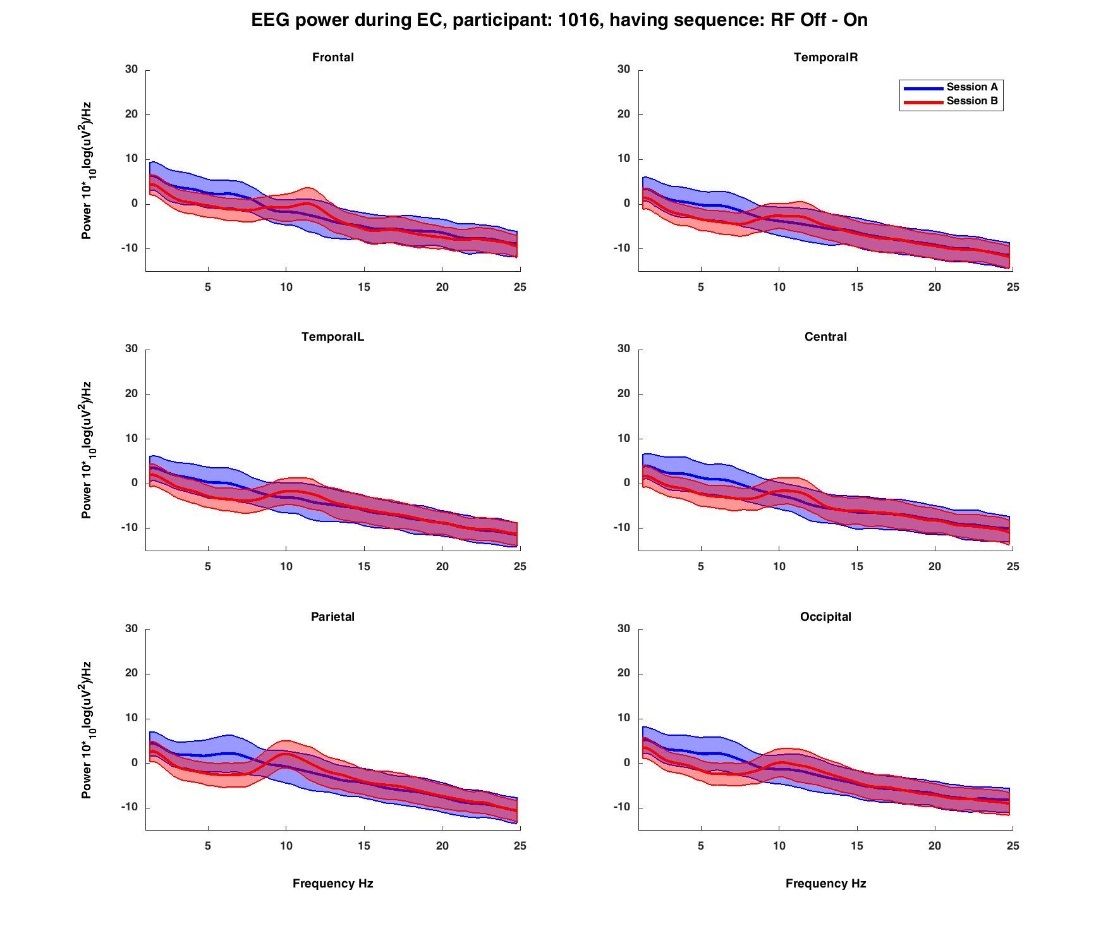


Figure S25. PSDs of participant 1016 for six brain regions in EC condition.


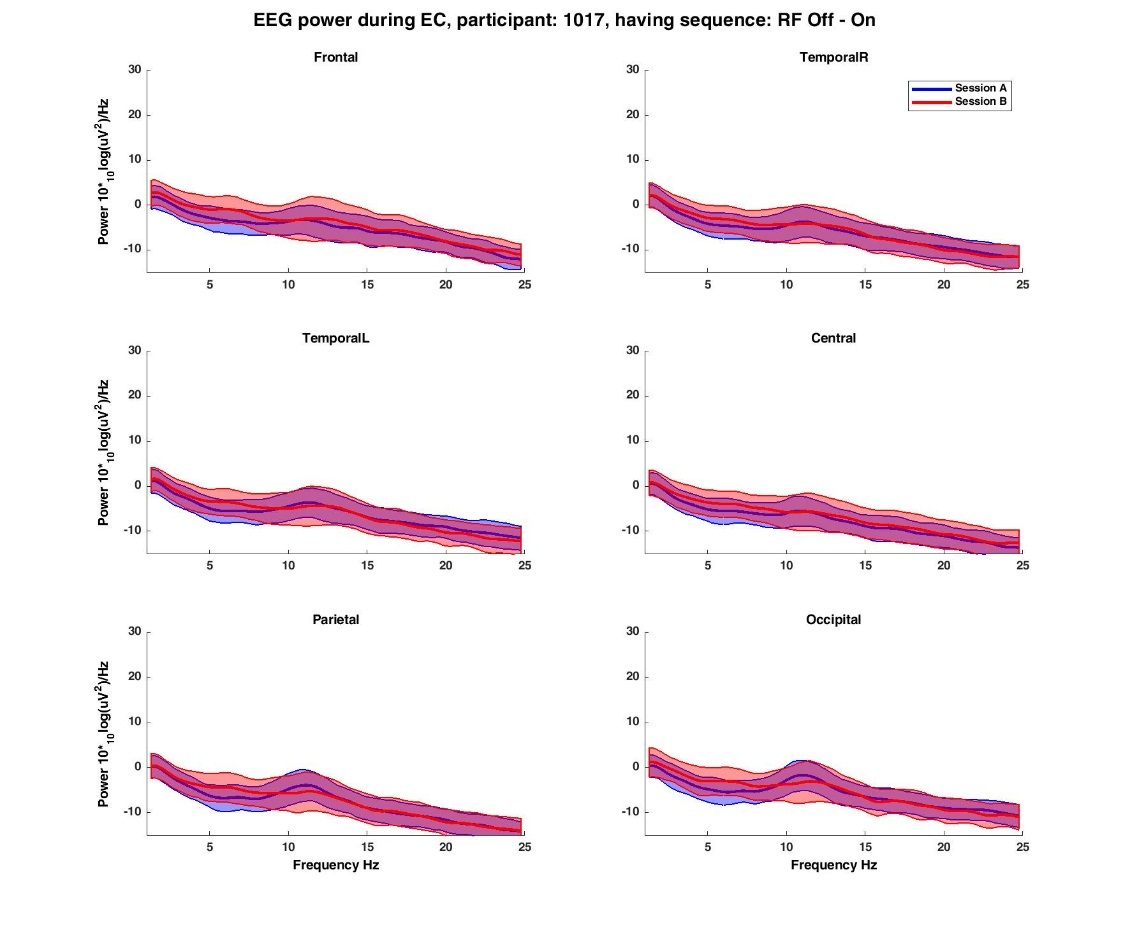


Figure S26. PSDs of participant 1017 for six brain regions in EC condition.


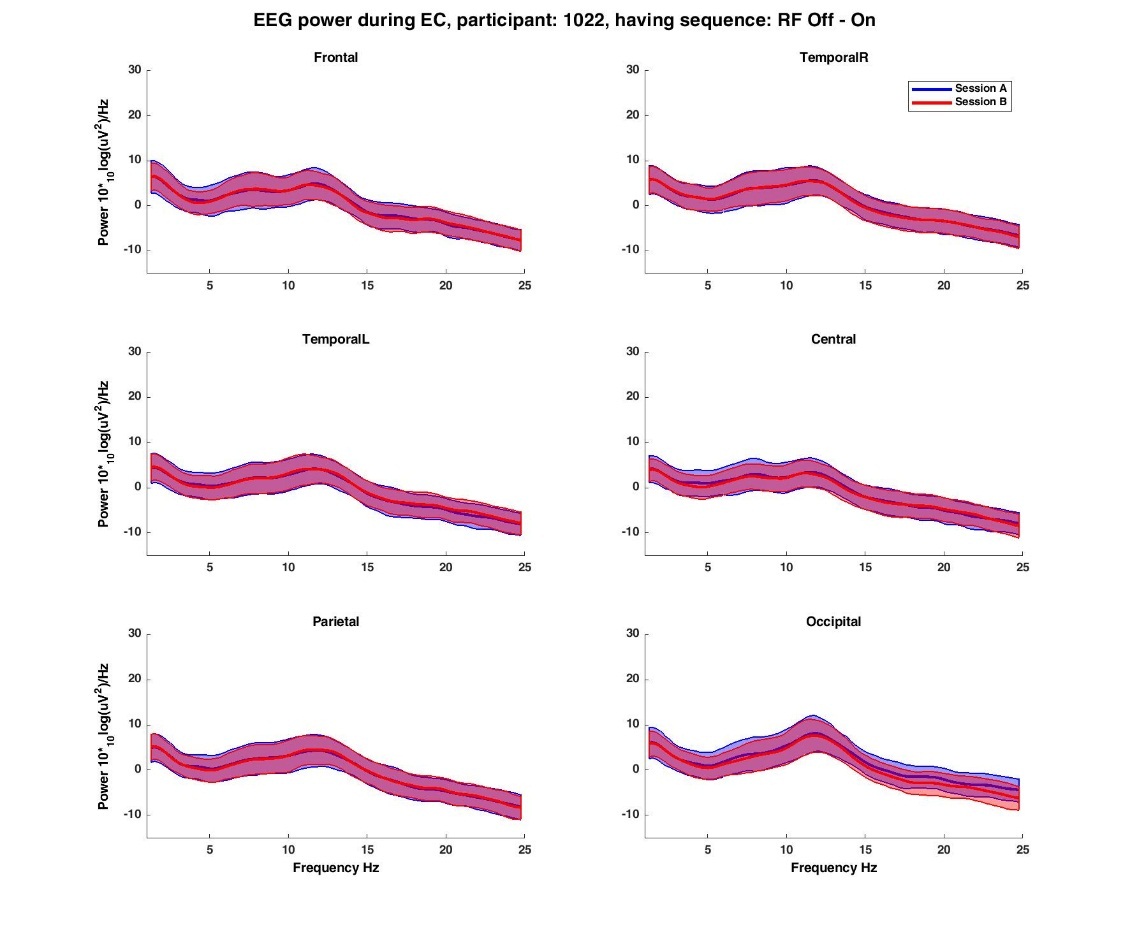


Figure S27 PSDs of participant 1022 for six brain regions in EC condition.


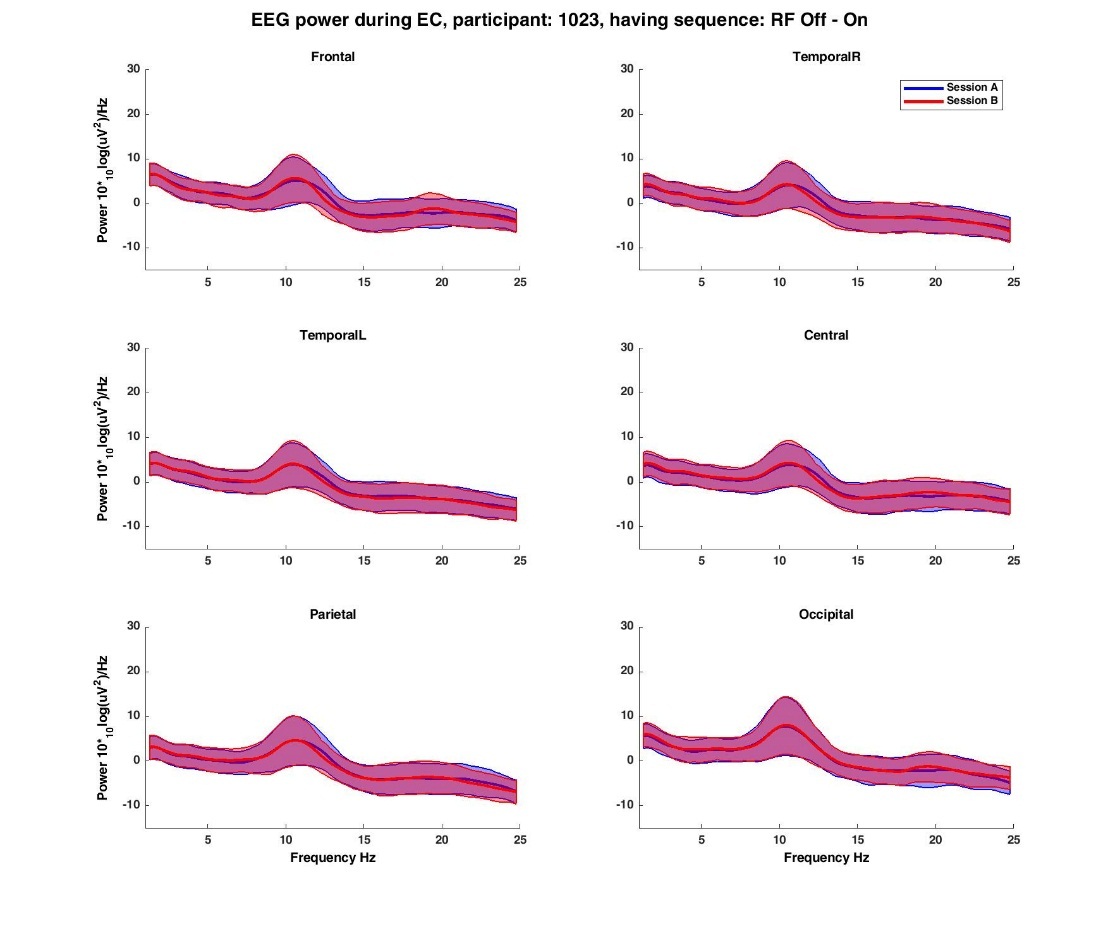


Figure S28. PSDs of participant 1023 for six brain regions in EC condition.


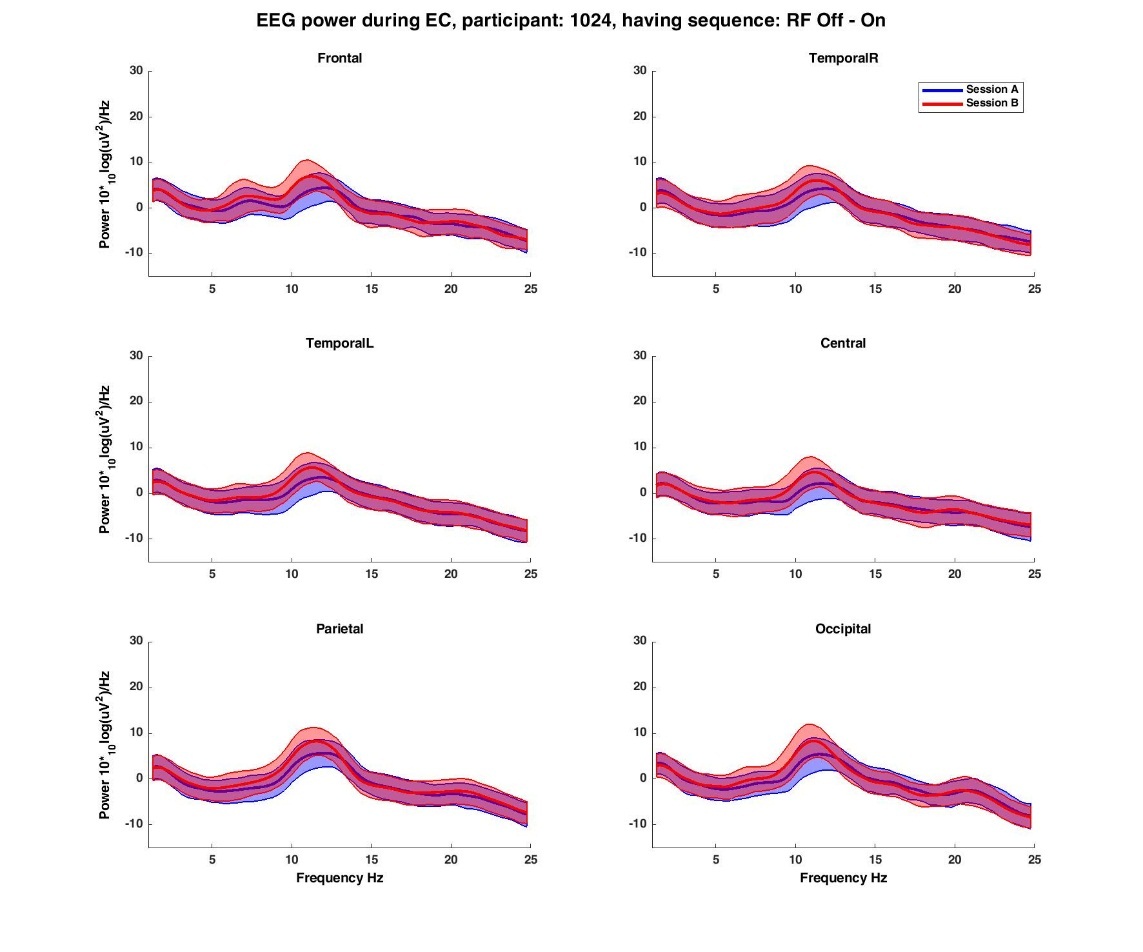


Figure S29. PSDs of participant 1024 for six brain regions in EC condition.


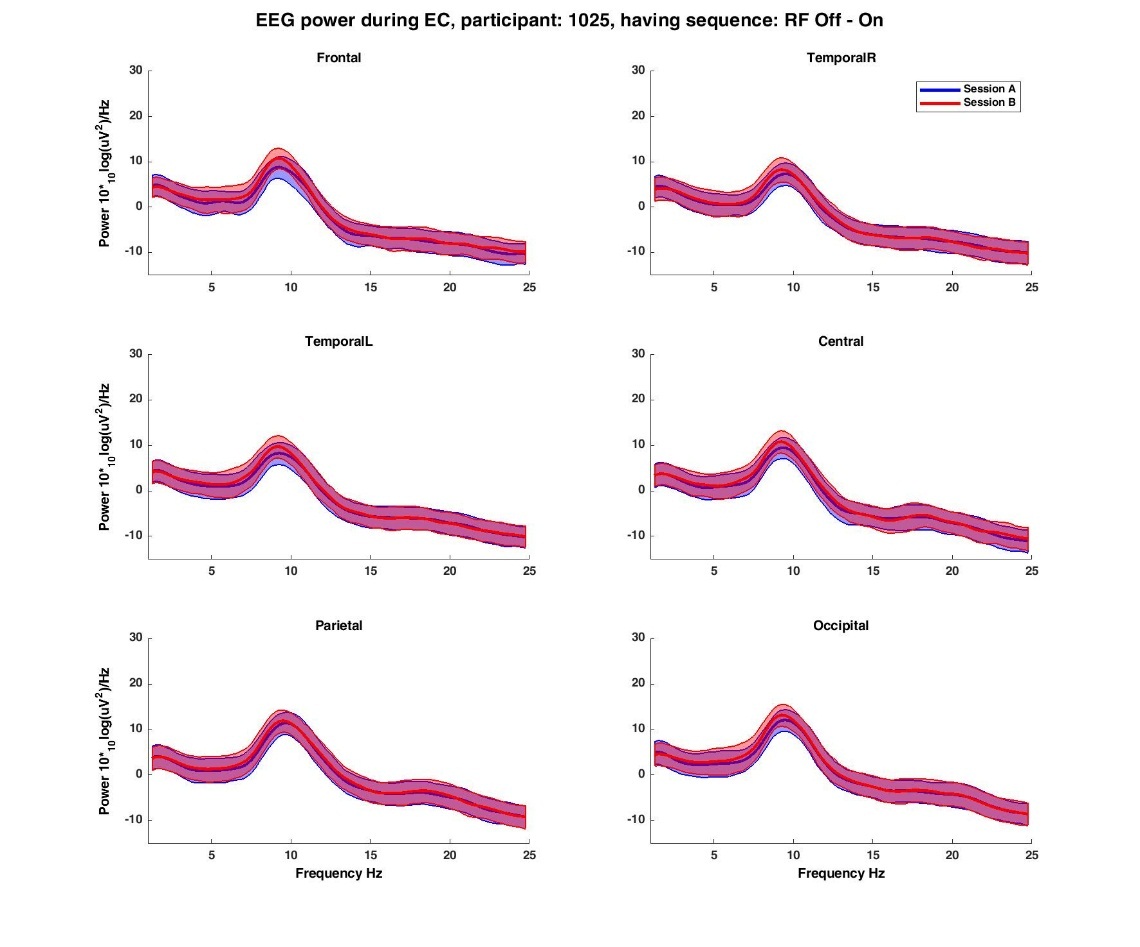


Figure S30. PSDs of participant 1025 for six brain regions in EC condition.


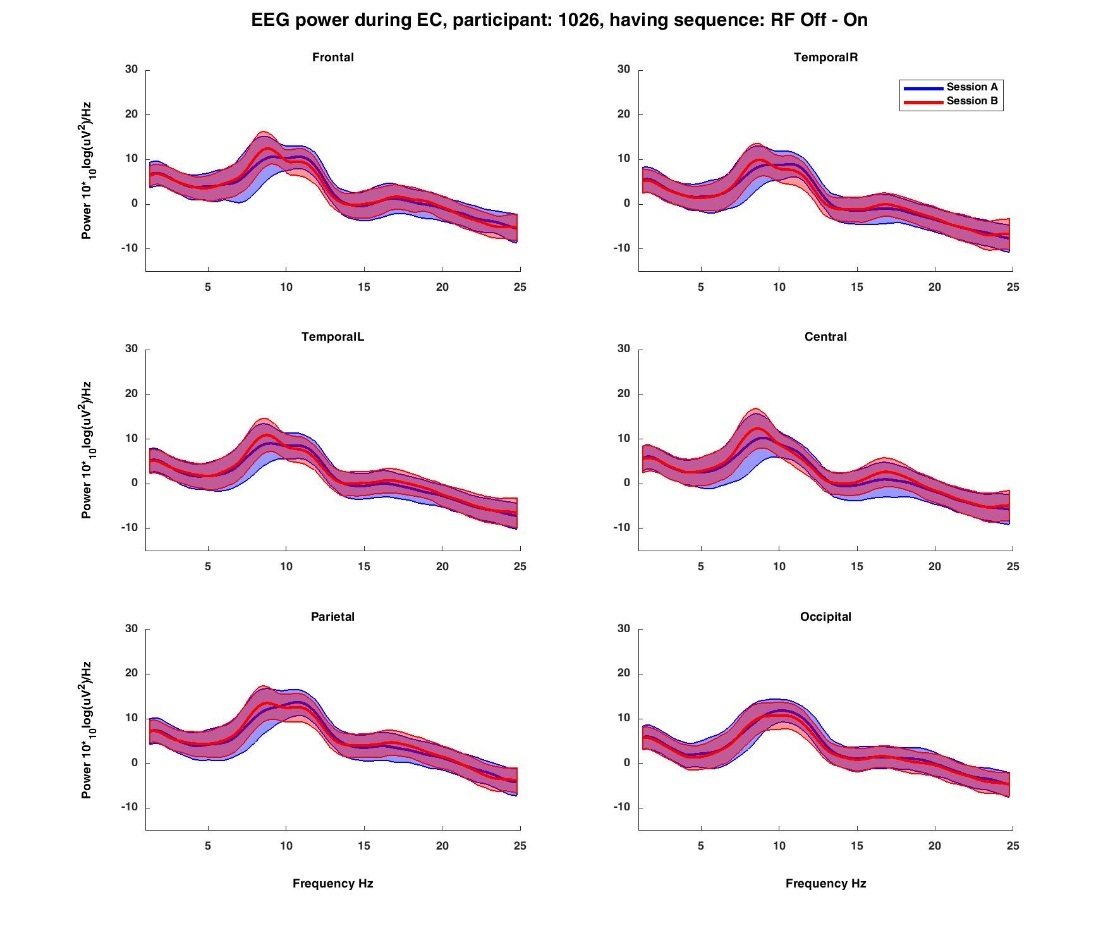


Figure S31. PSDs of participant 1026 for six brain regions in EC condition.


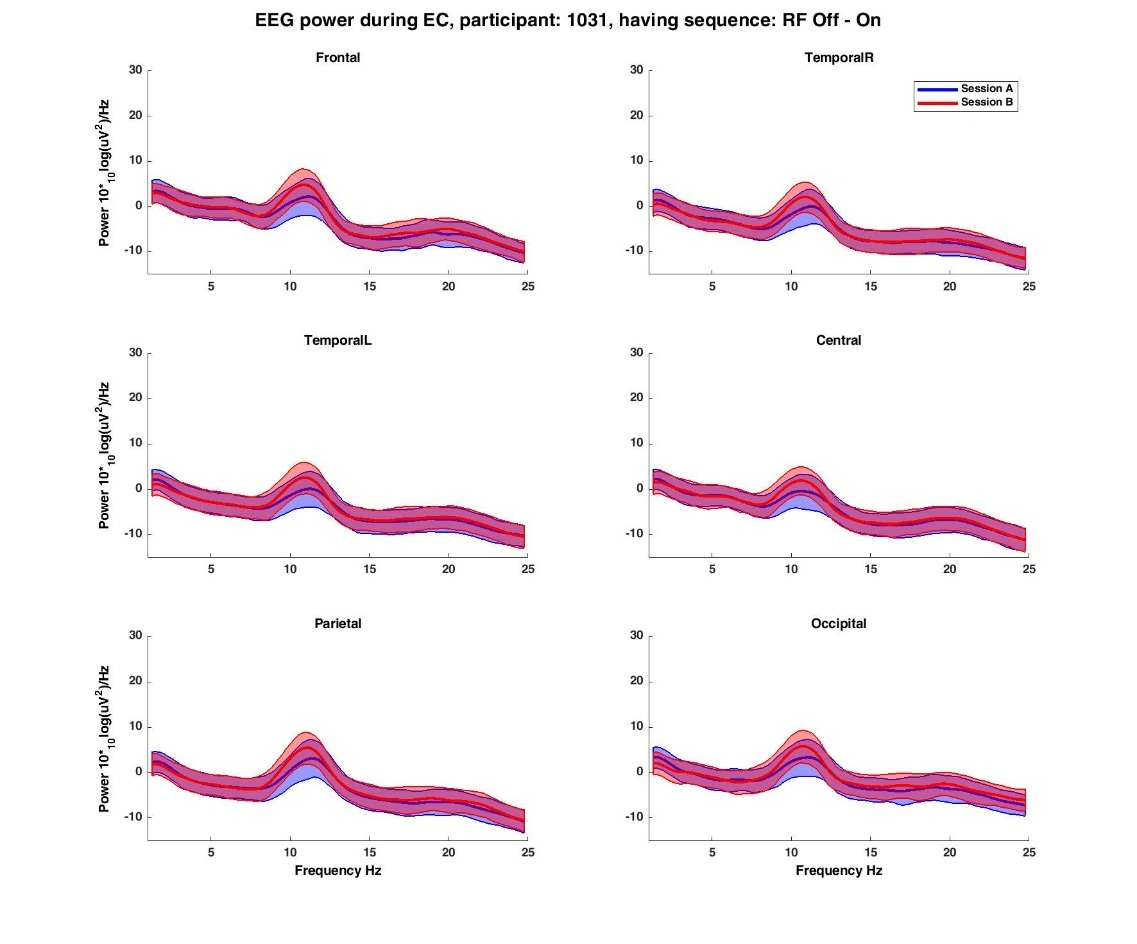


Figure S32. PSDs of participant 1031 for six brain regions in EC condition.

EO, Group 1 (RF in session A)


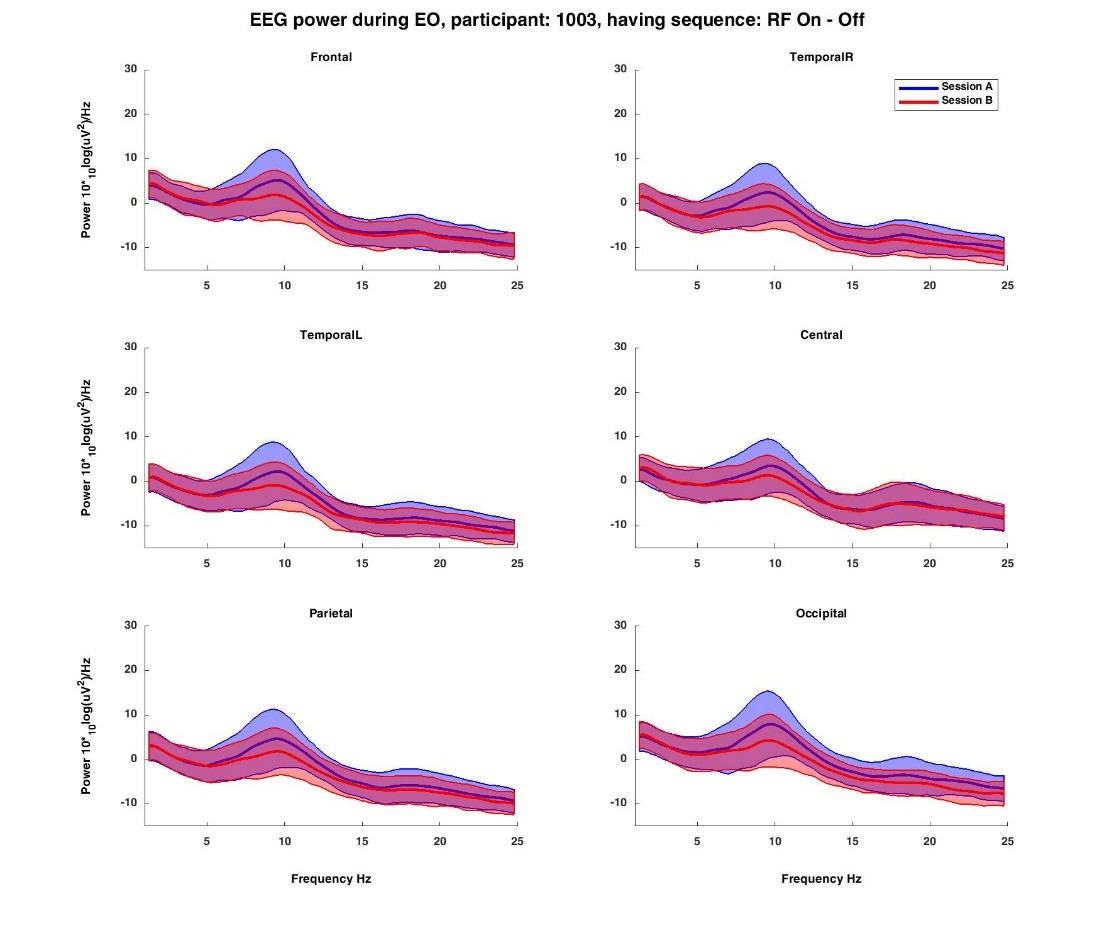


Figure S33. PSDs of participant 1003 for six brain regions in EO condition.


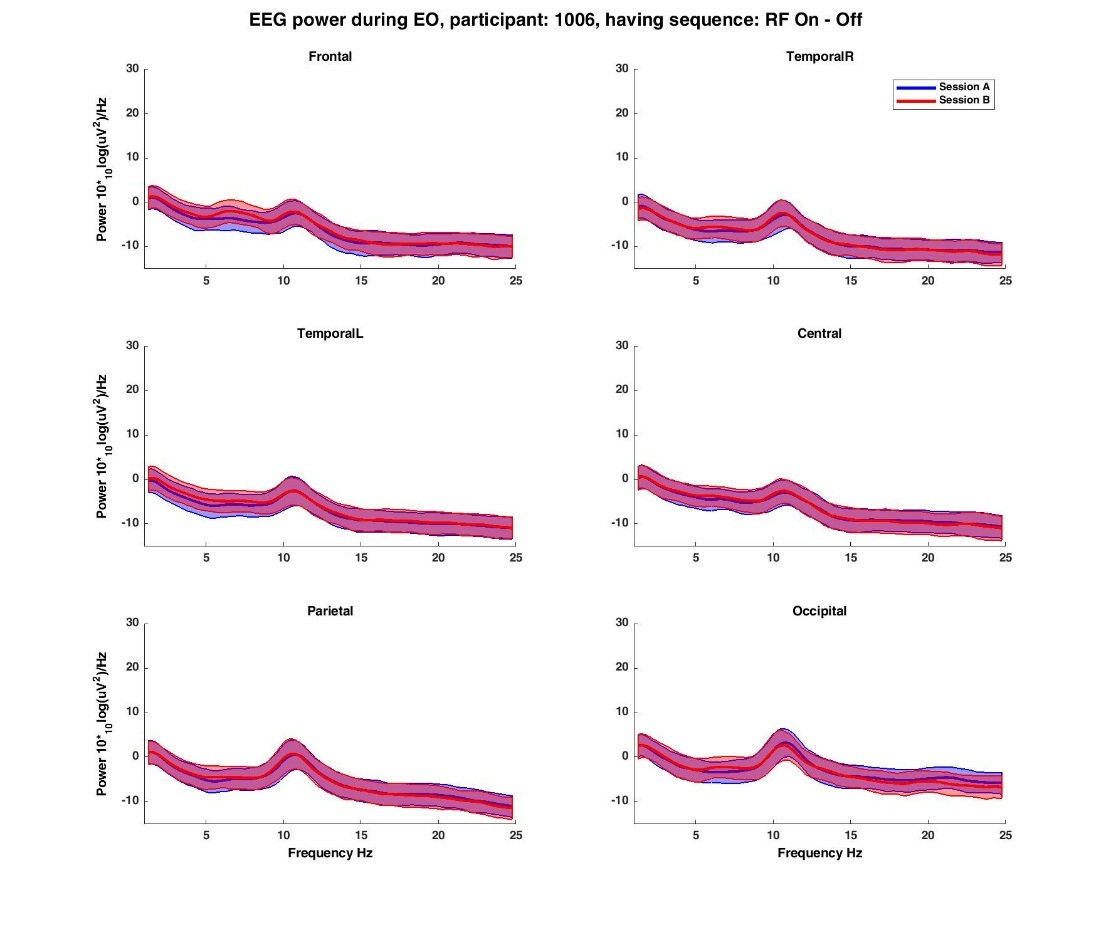


Figure S34. PSDs of participant 1006 for six brain regions in EO condition.


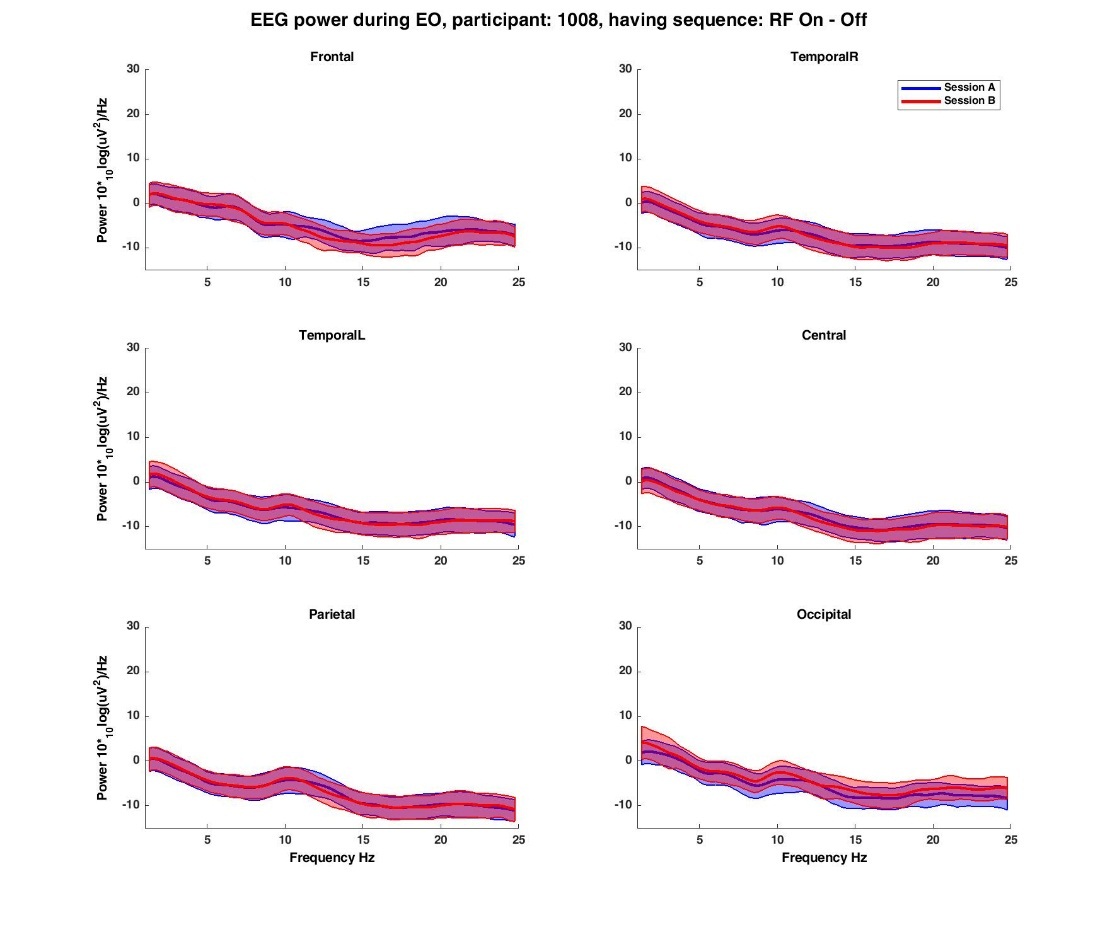


Figure S35. PSDs of participant 1008 for six brain regions in EO condition.


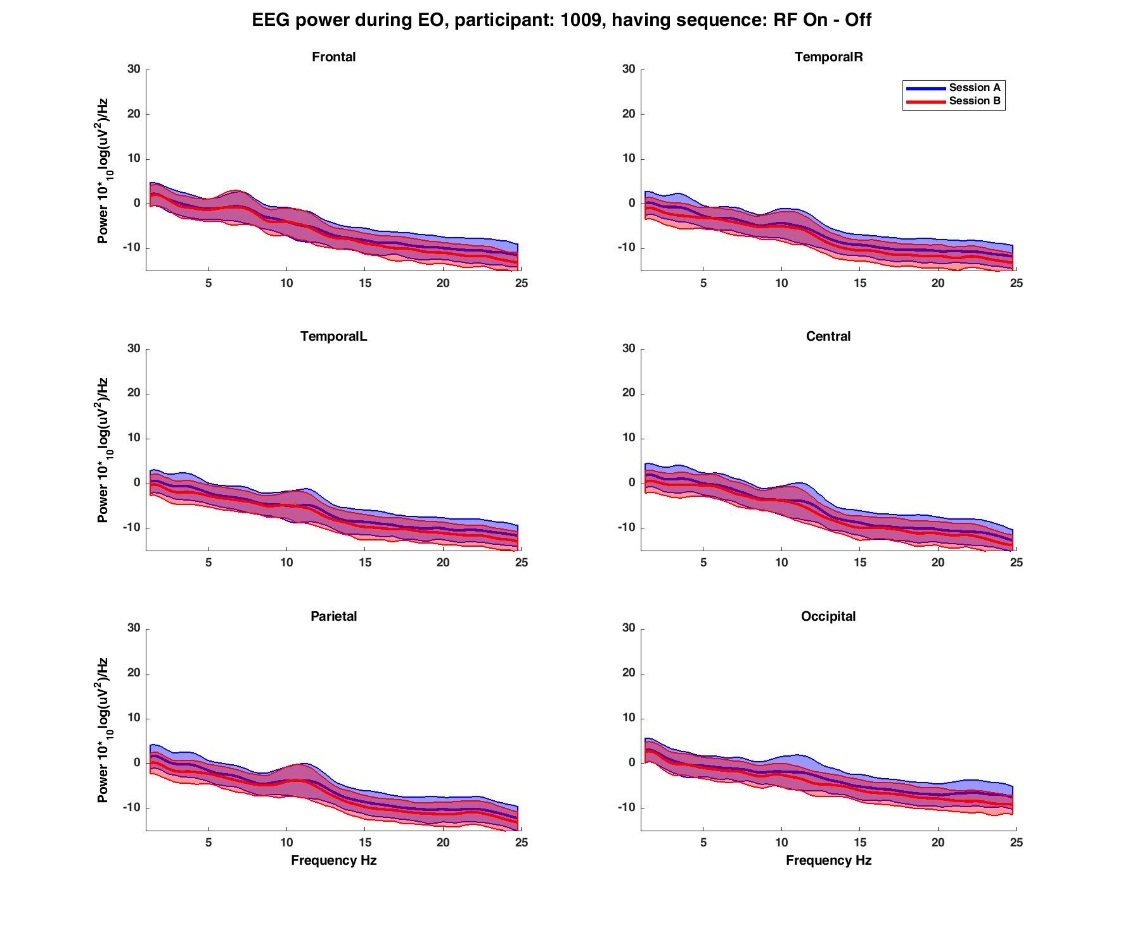


Figure S36. PSDs of participant 1006 for six brain regions in EO condition.


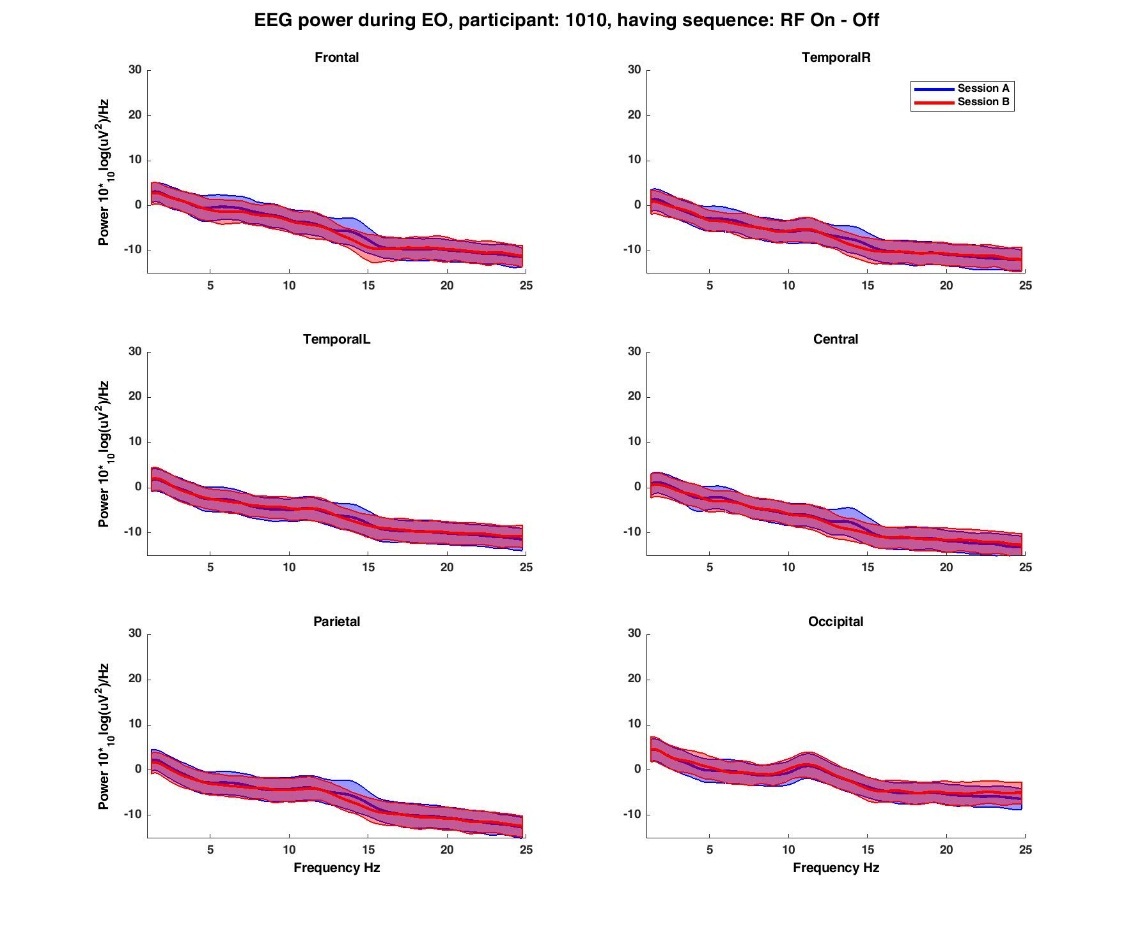


Figure S37. PSDs of participant 1010 for six brain regions in EO condition.


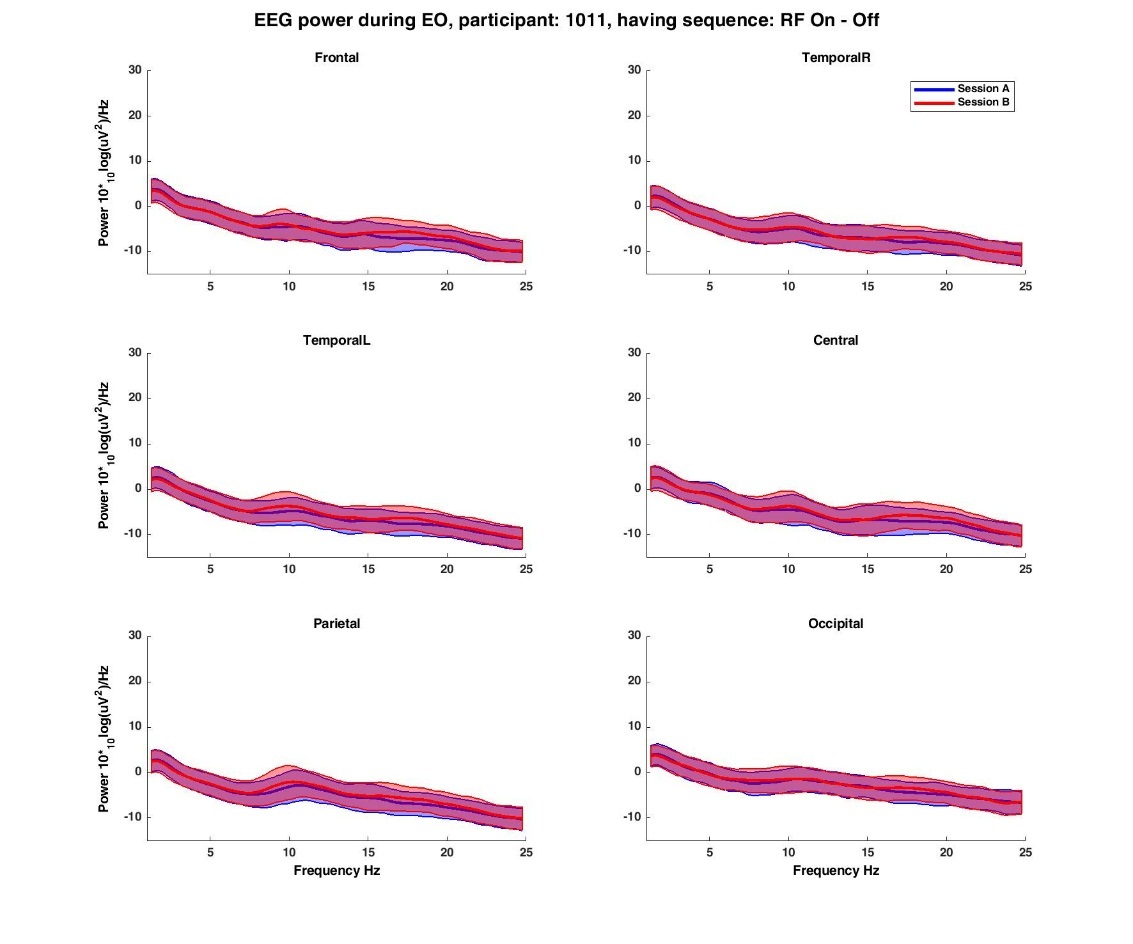


Figure S38. PSDs of participant 1011 for six brain regions in EO condition.


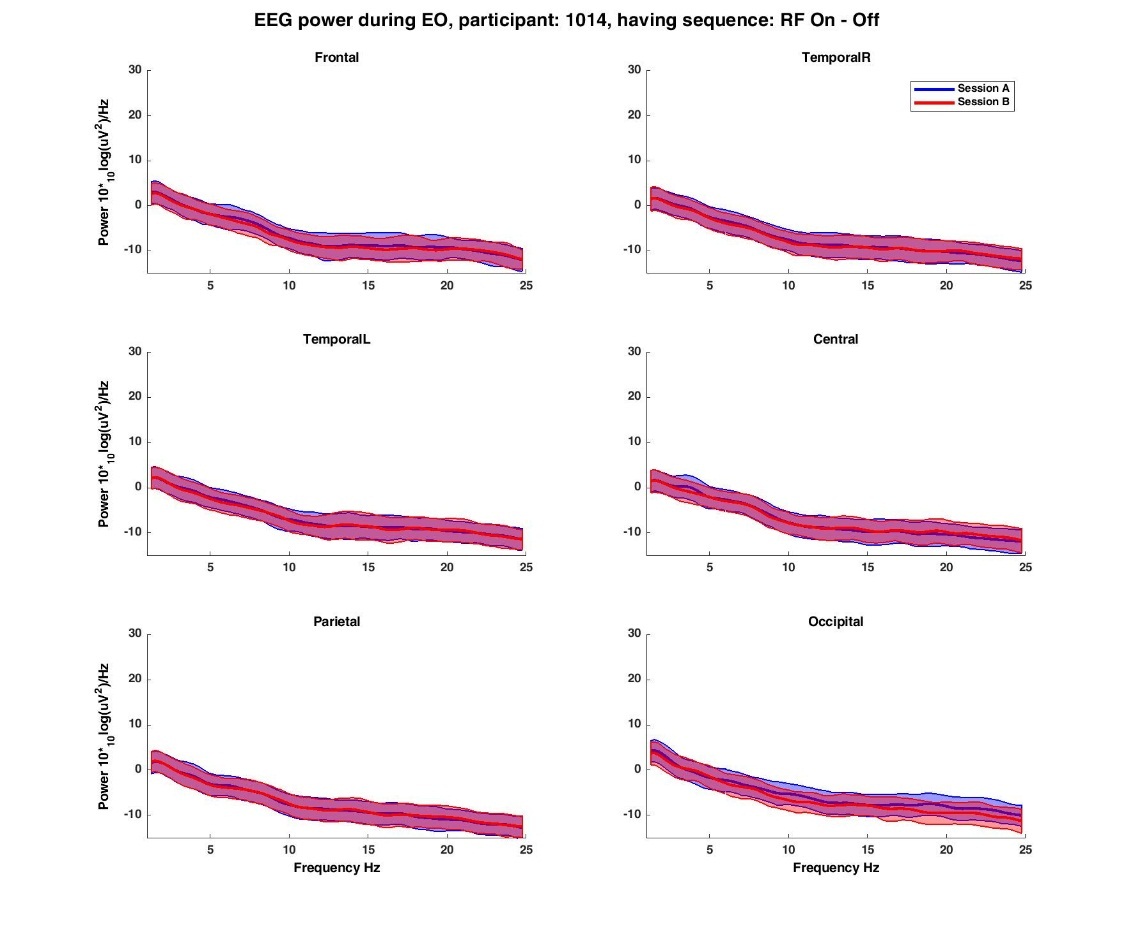


Figure S39. PSDs of participant 1014 for six brain regions in EO condition.


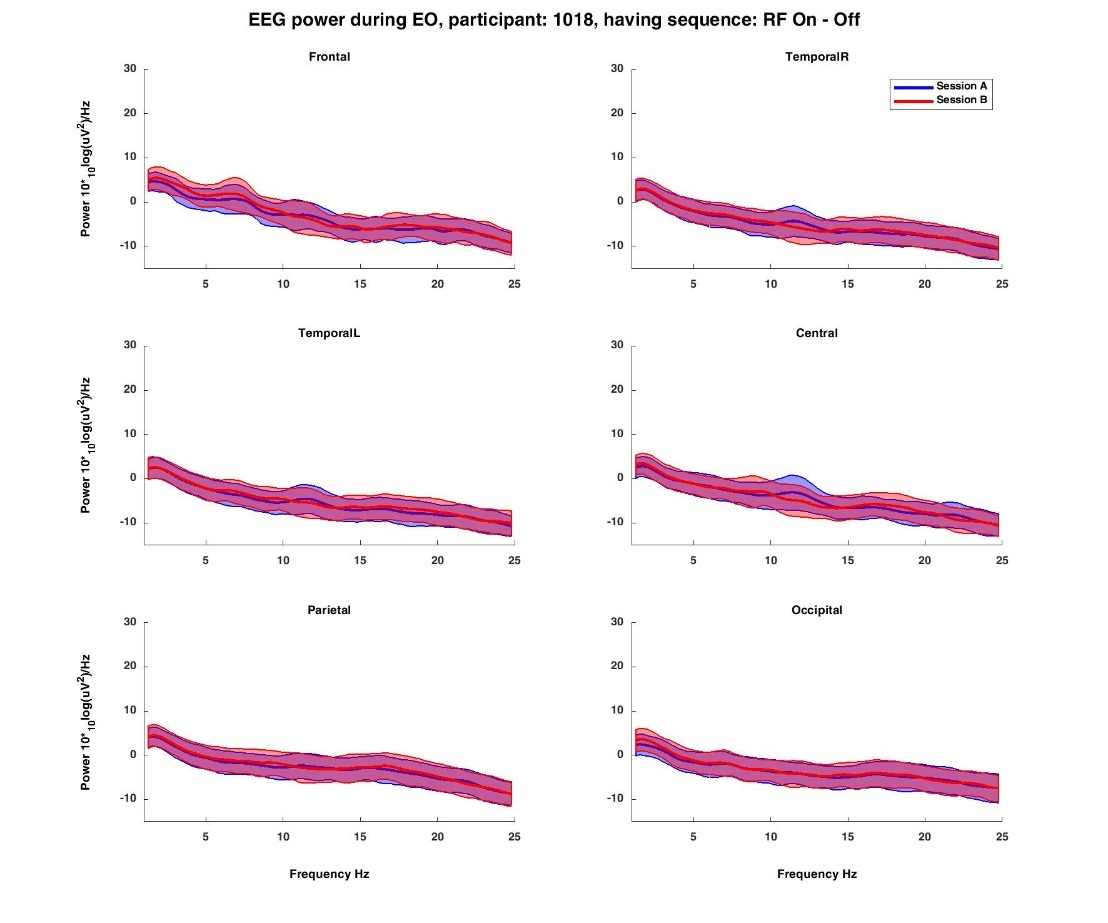


Figure S40. PSDs of participant 1018 for six brain regions in EO condition.


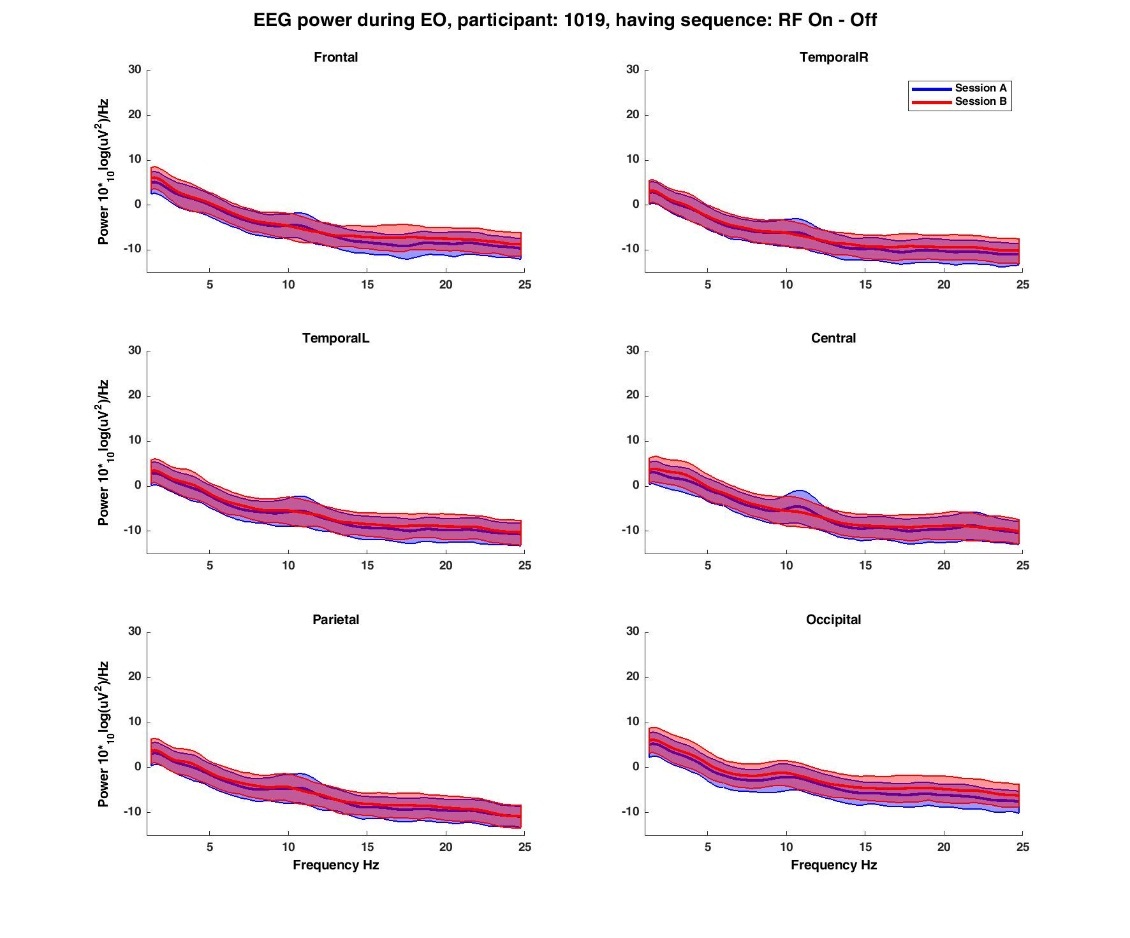


Figure S41. PSDs of participant 1019 for six brain regions in EO condition.


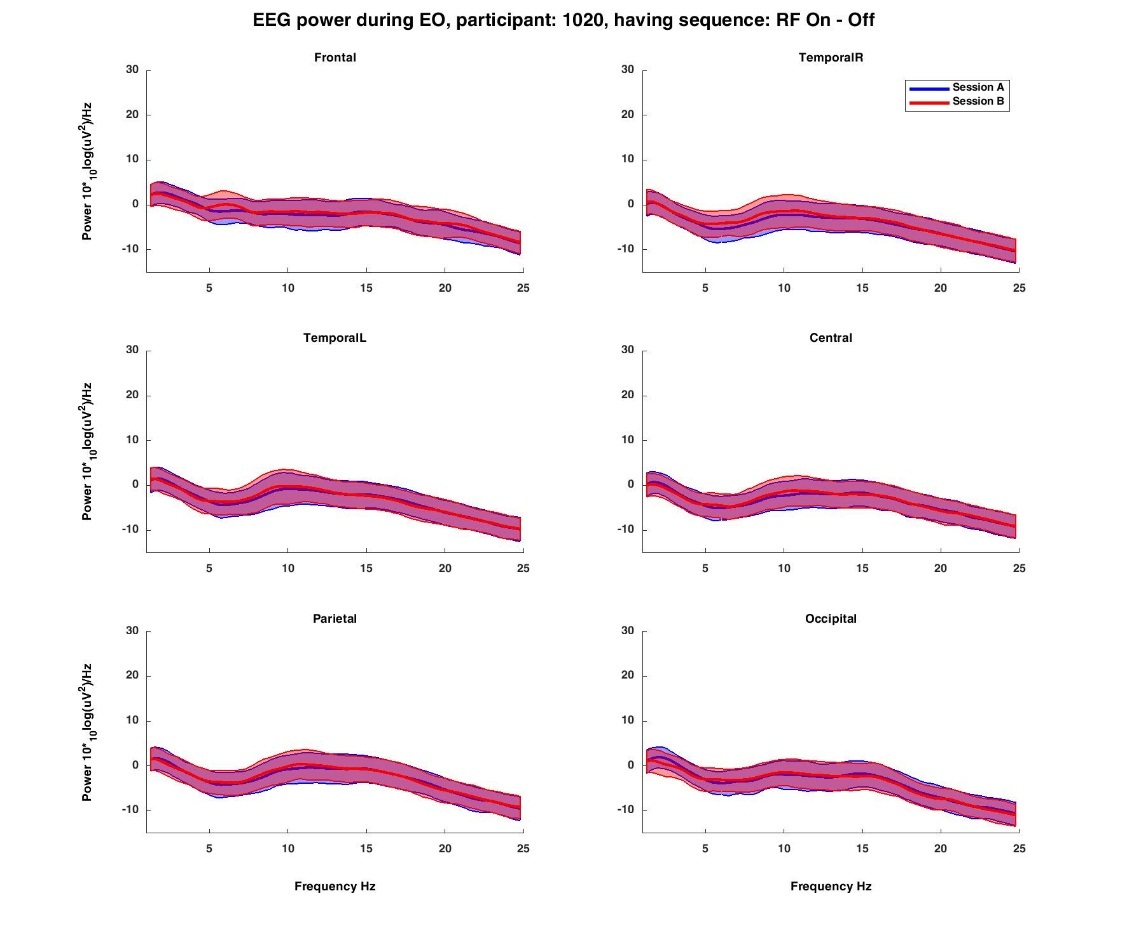


Figure S42. PSDs of participant 1020 for six brain regions in EO condition.


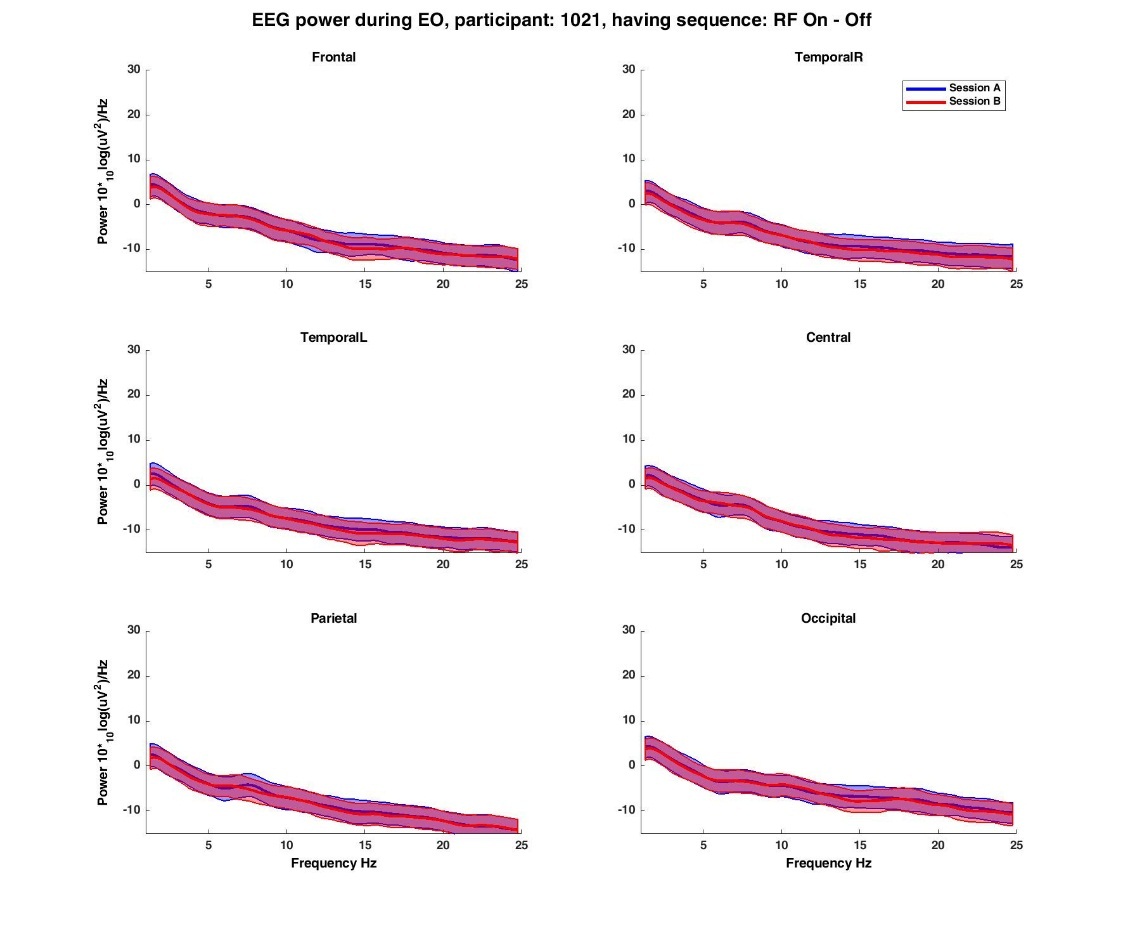


Figure S43. PSDs of participant 1021 for six brain regions in EO condition.


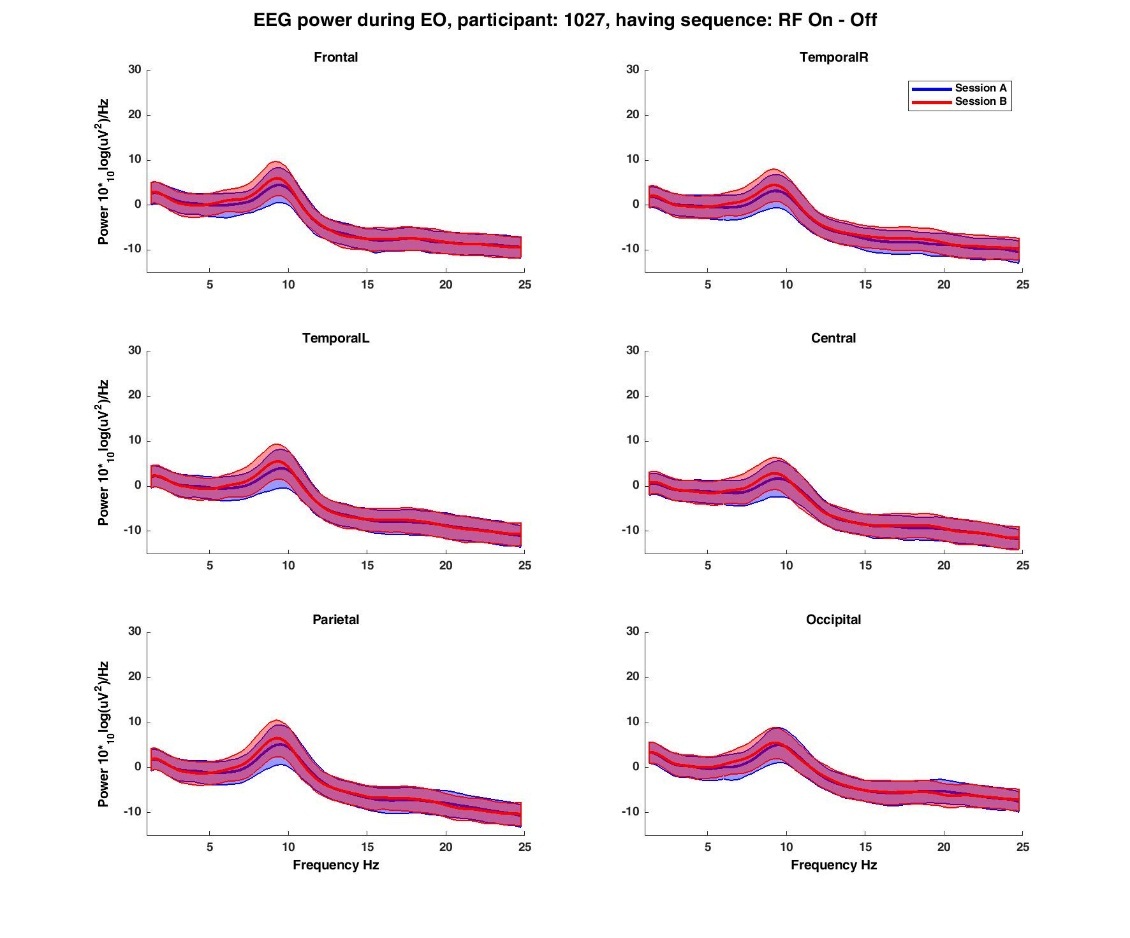


Figure S44. PSDs of participant 1027 for six brain regions in EO condition.


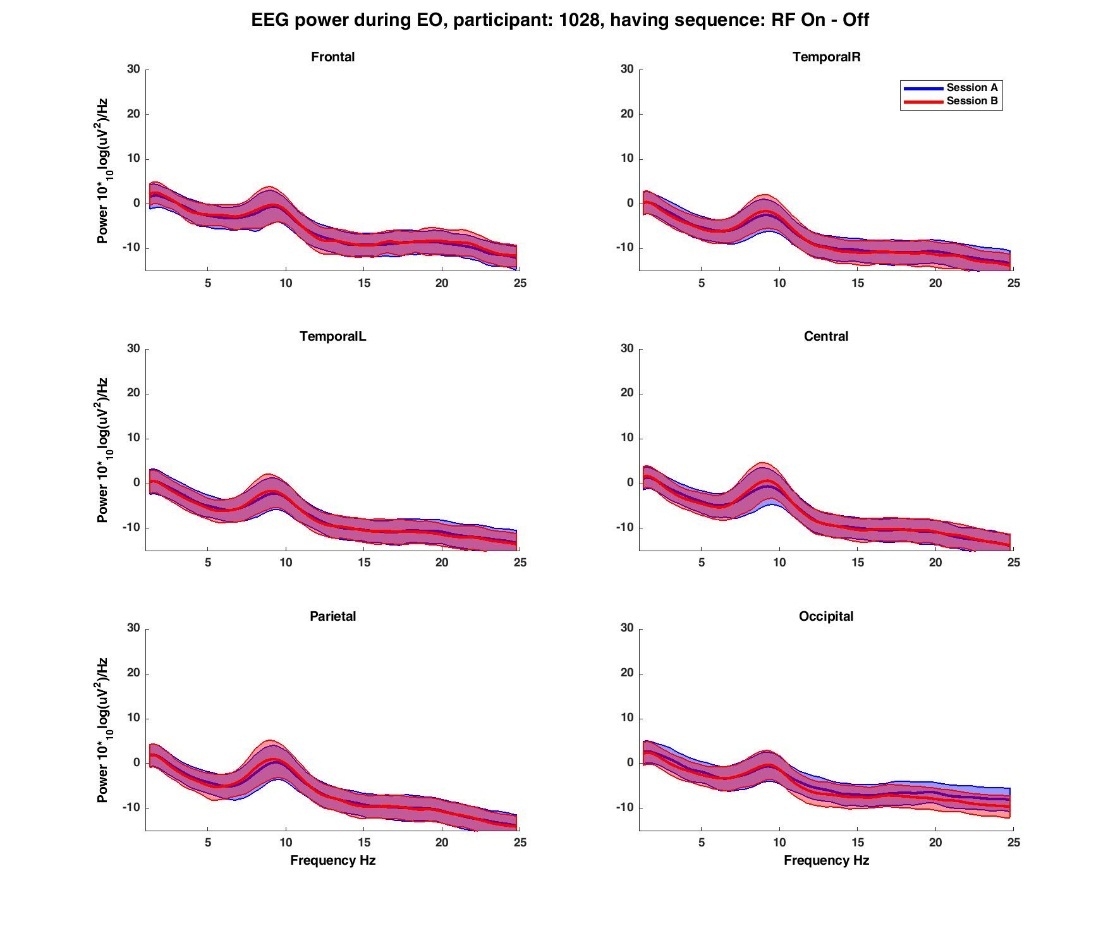


Figure S45. PSDs of participant 1028 for six brain regions in EO condition.


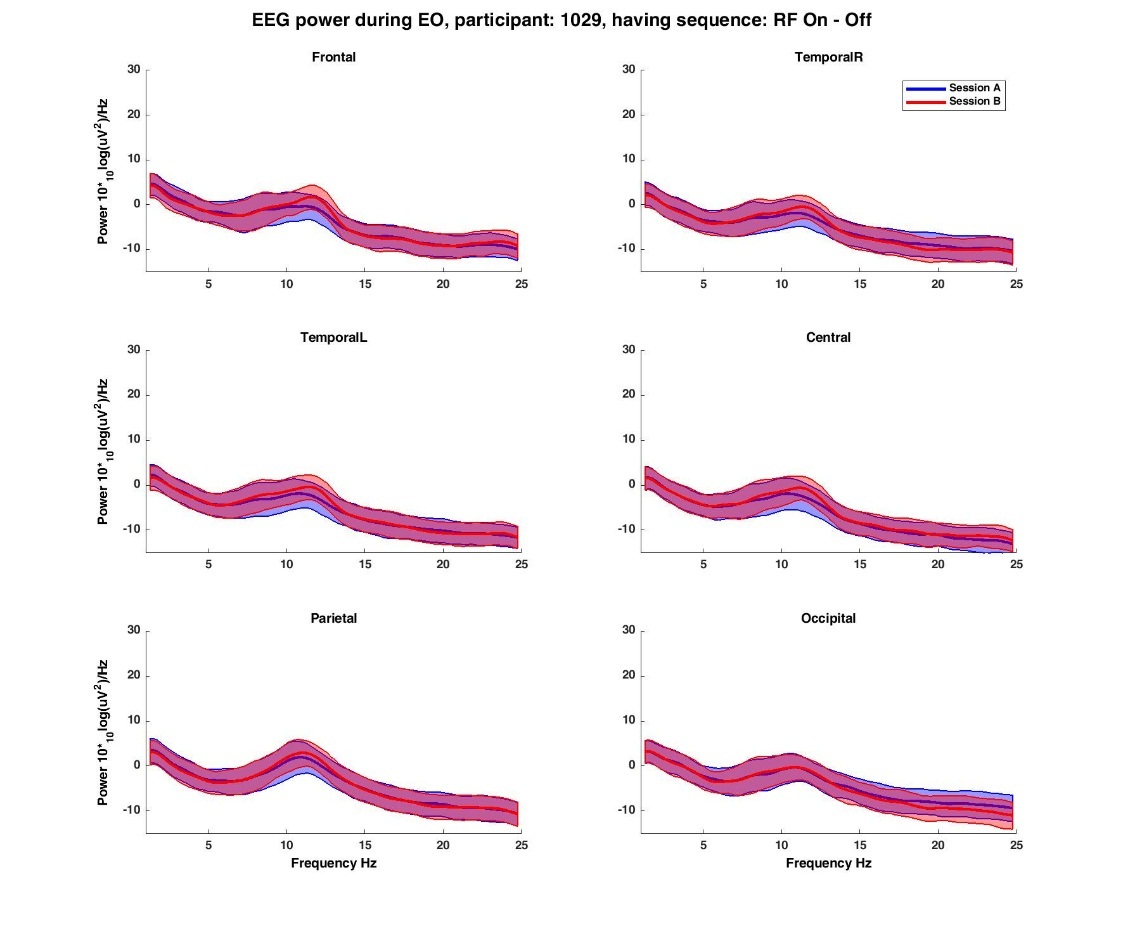


Figure S46. PSDs of participant 1029 for six brain regions in EO condition.


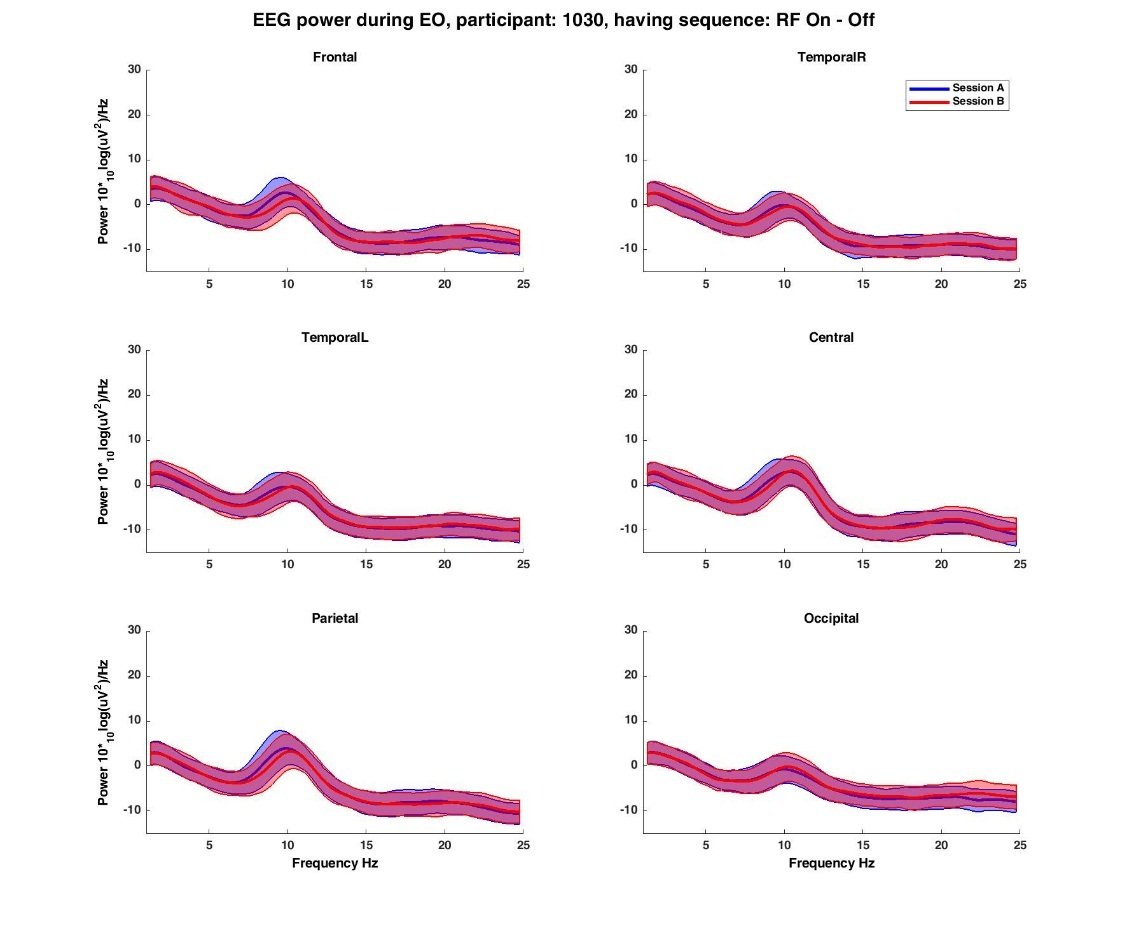


Figure S47. PSDs of participant 1030 for six brain regions in EO condition.


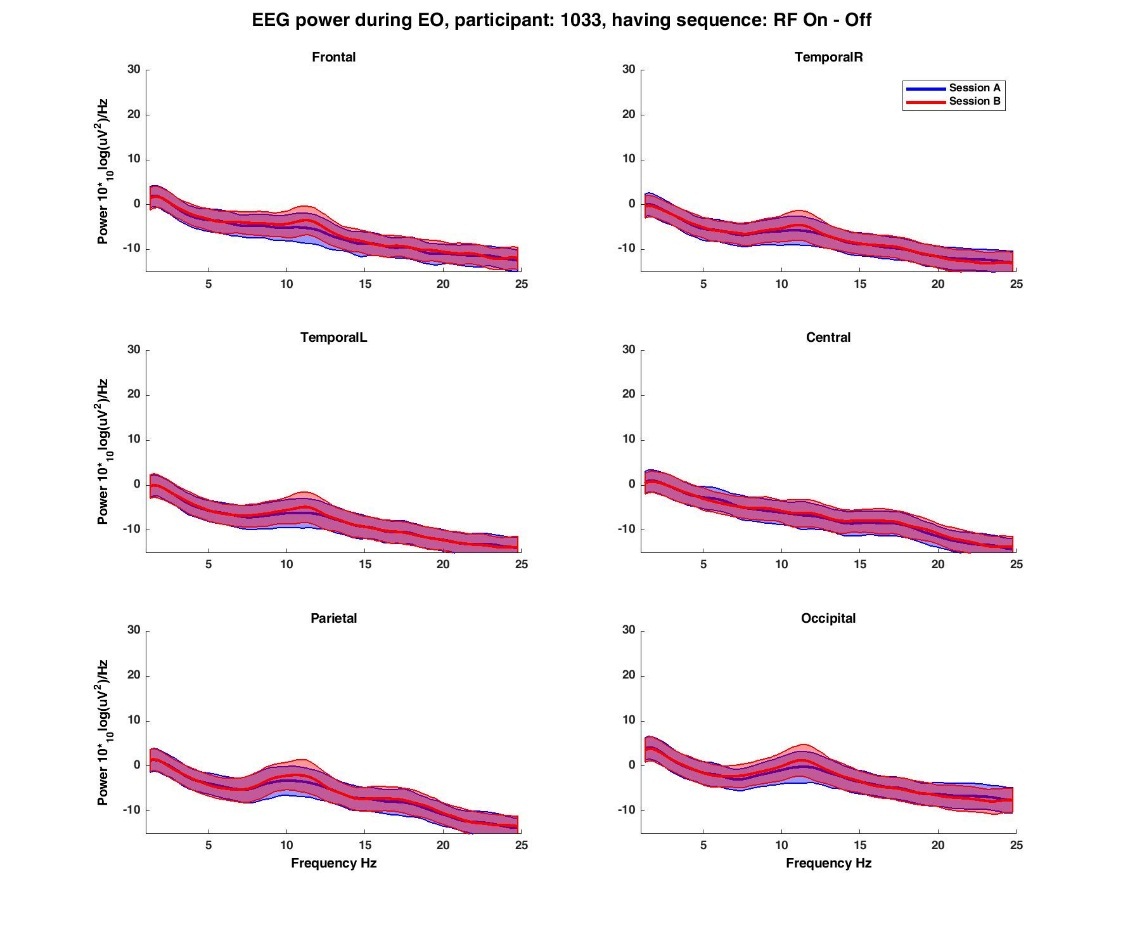


Figure S48. PSDs of participant 1033 for six brain regions in EO condition.

EO, Group 2 (RF in session B)


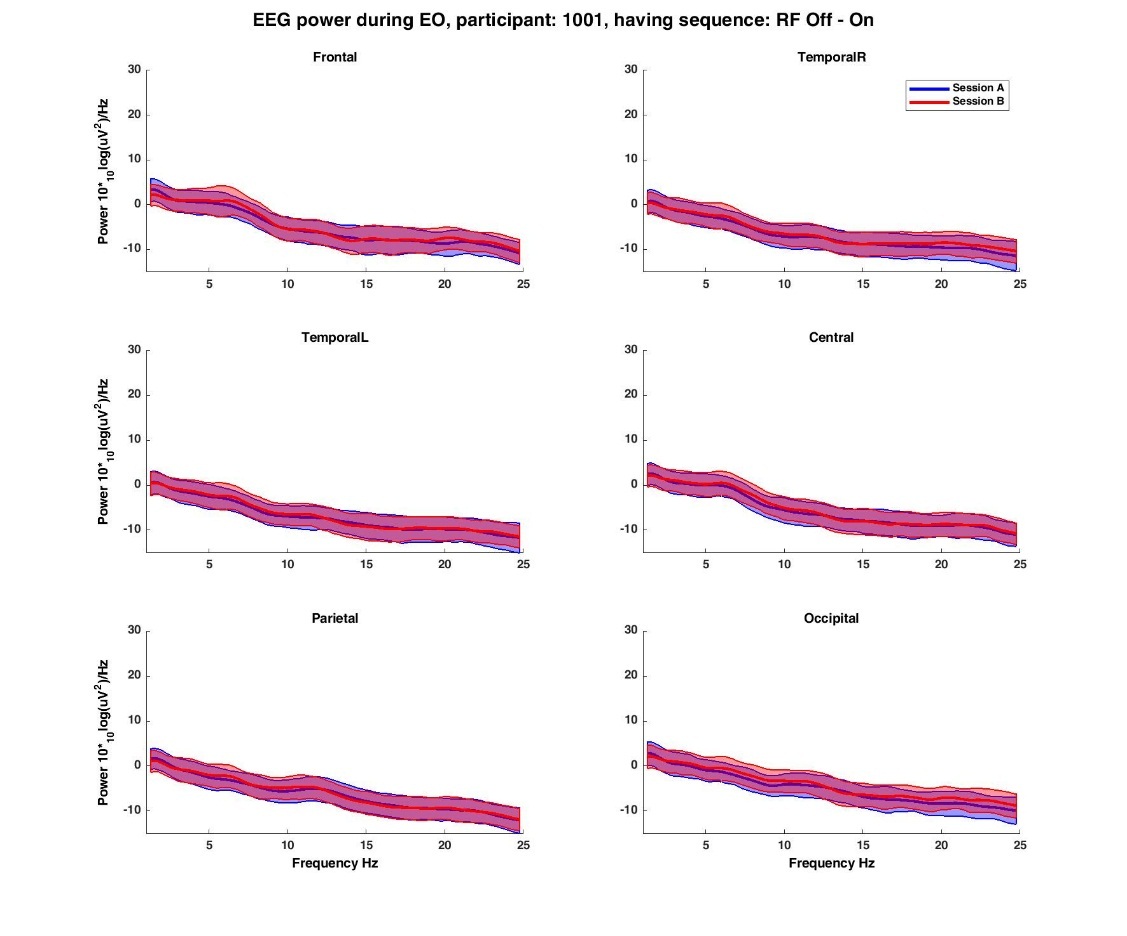


Figure S49. PSDs of participant 1001 for six brain regions in EO condition.


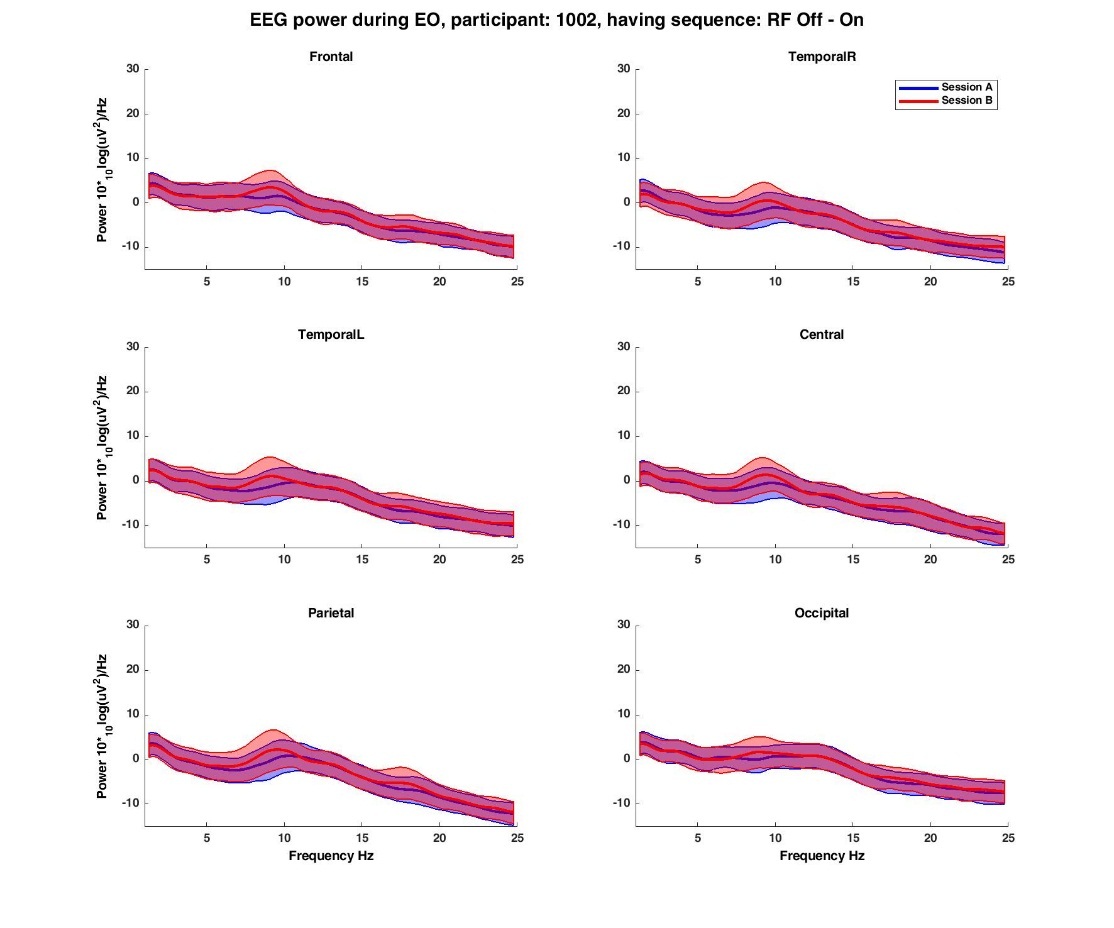


Figure S50. PSDs of participant 1002 for six brain regions in EO condition.


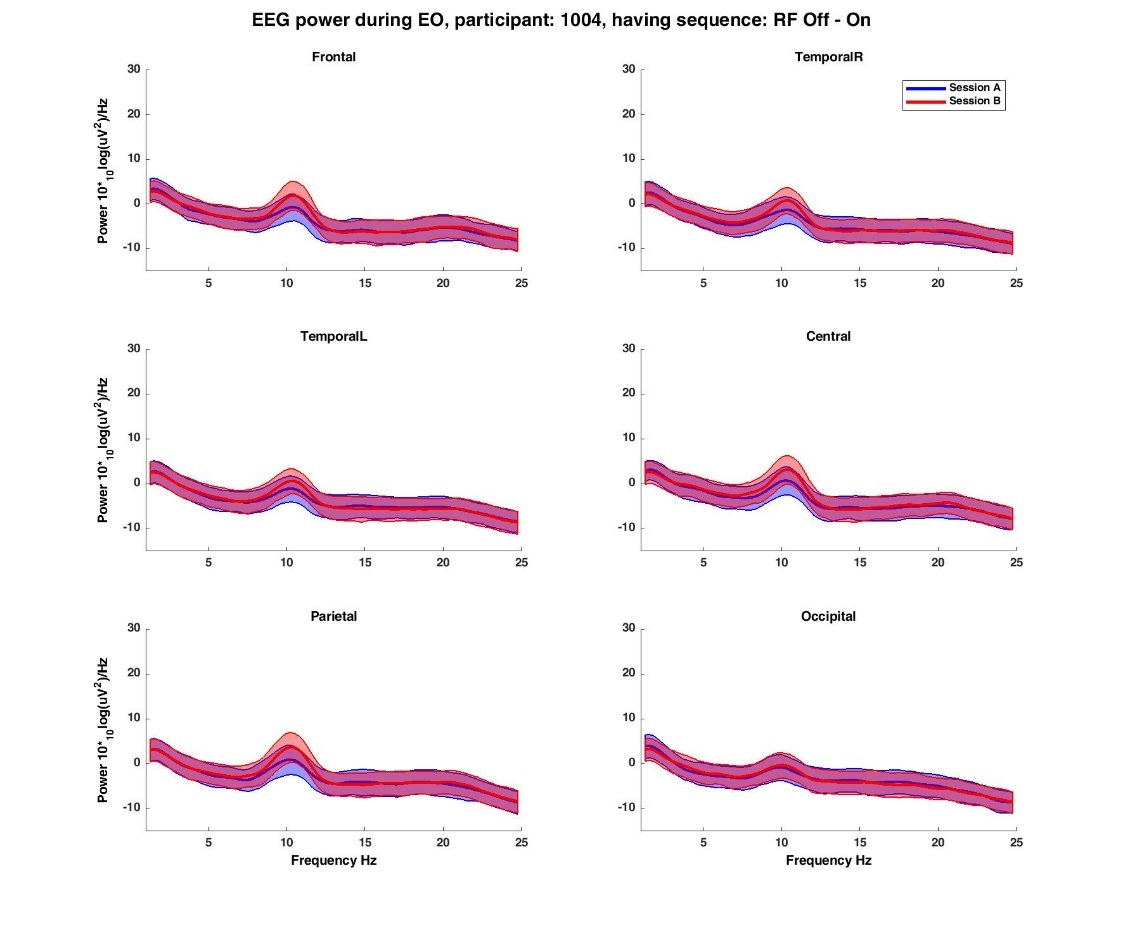


Figure S51. PSDs of participant 1004 for six brain regions in EO condition.


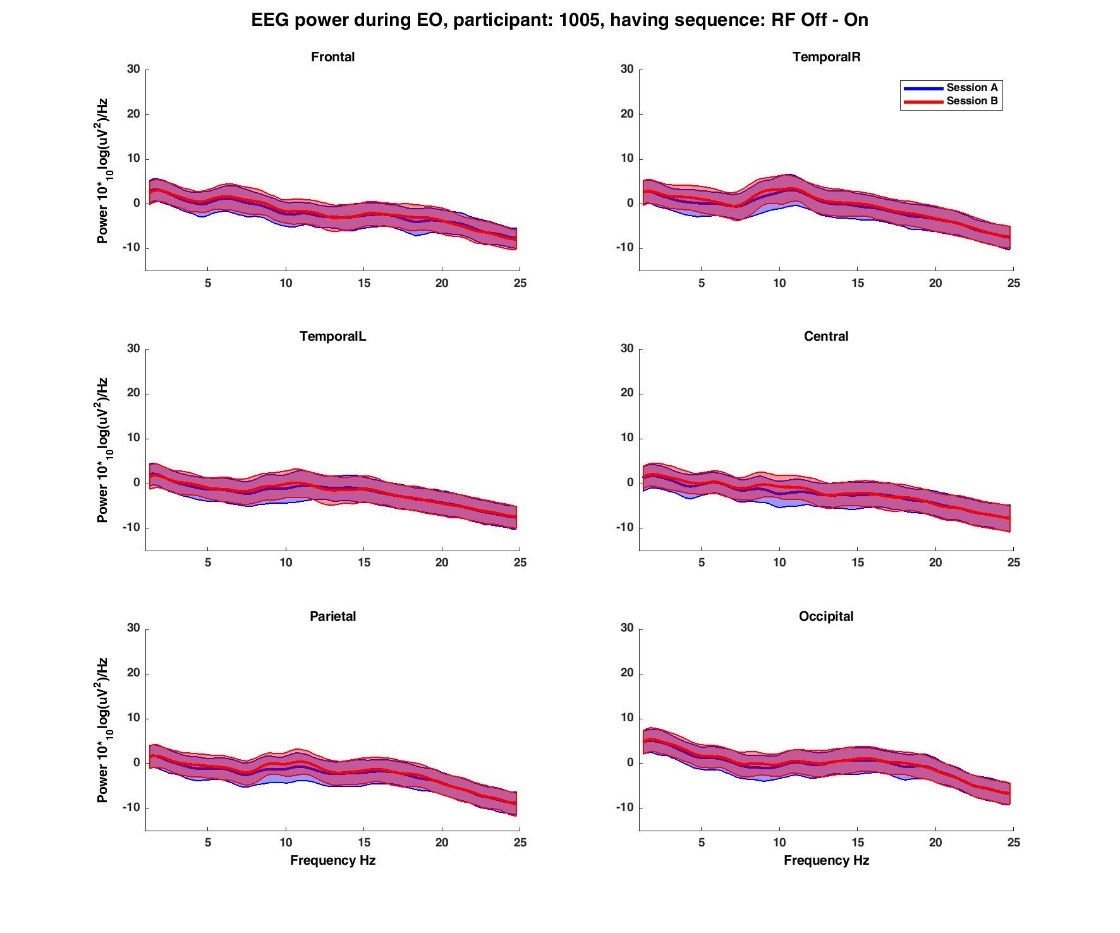


Figure S52. PSDs of participant 1005 for six brain regions in EO condition.


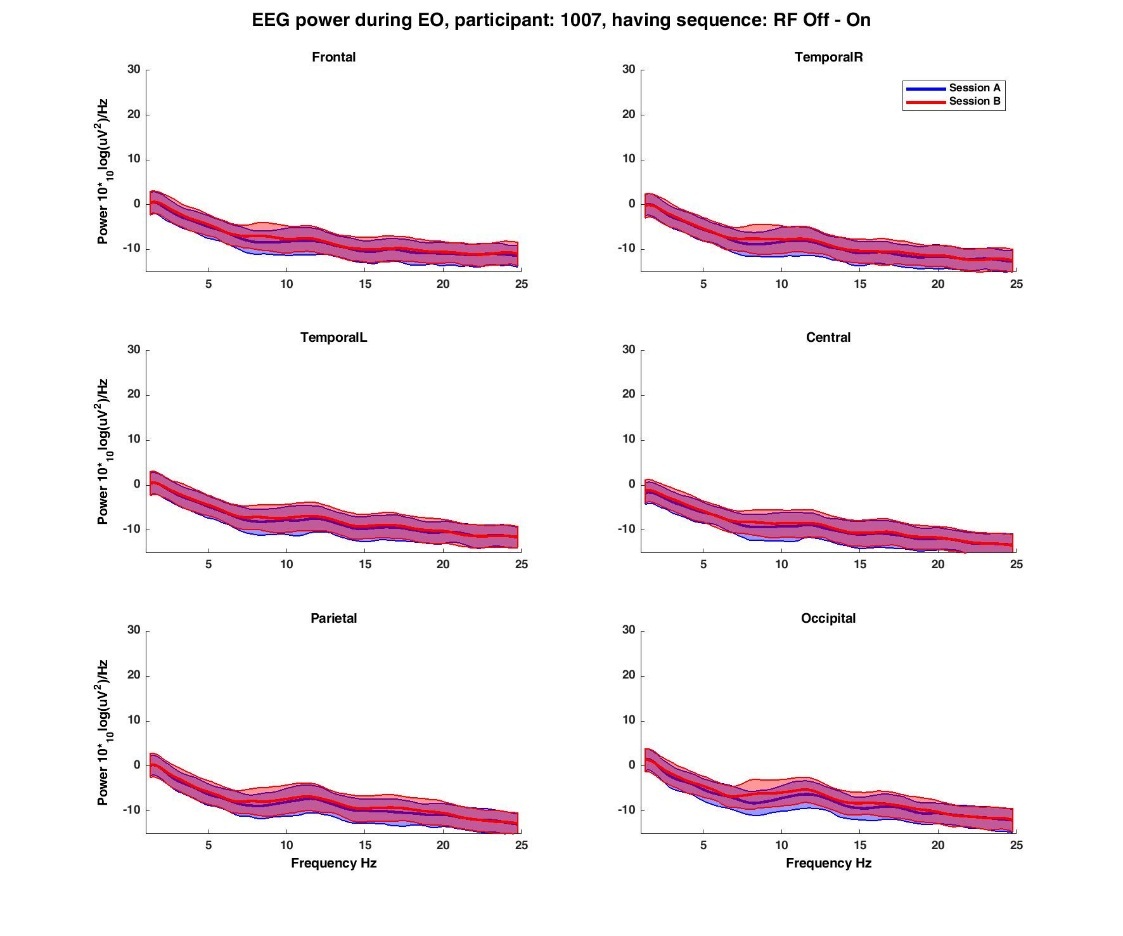


Figure S53. PSDs of participant 1007 for six brain regions in EO condition.


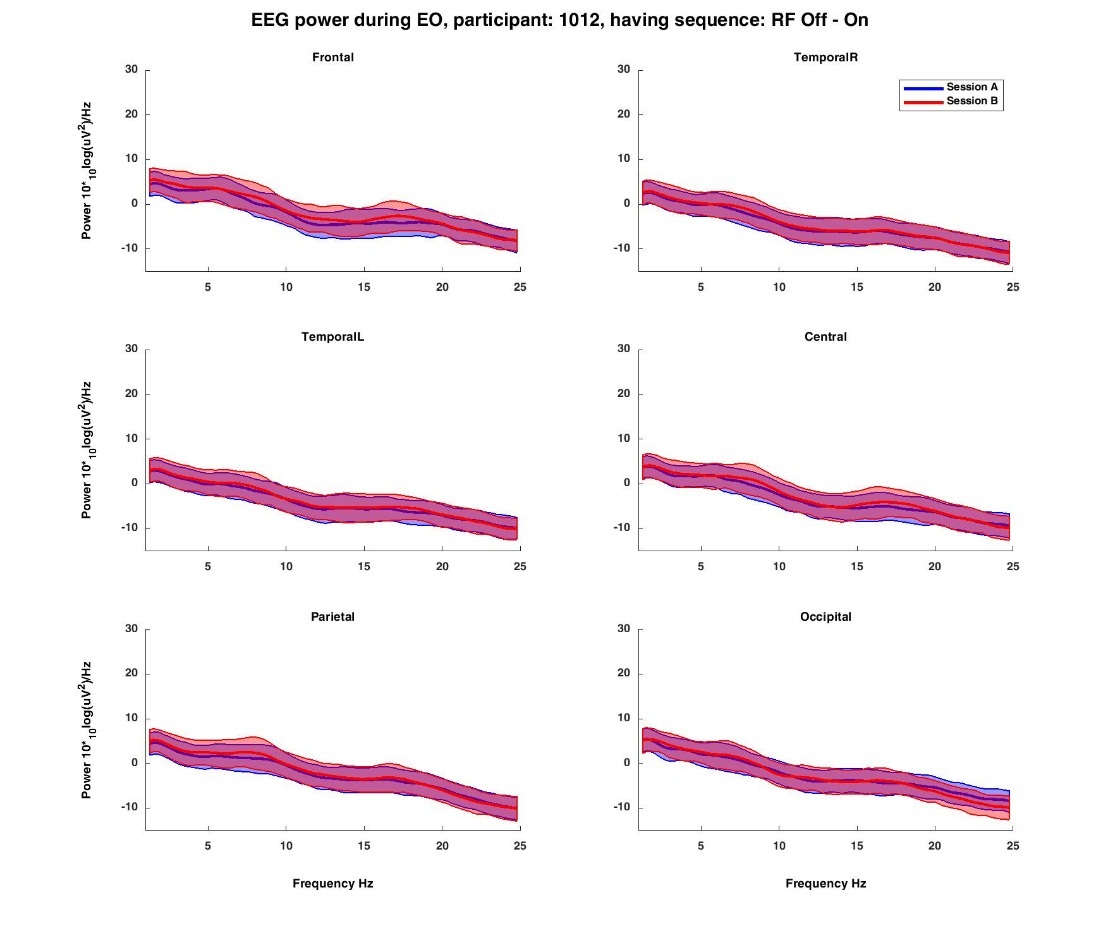


Figure S54. PSDs of participant 1012 for six brain regions in EO condition.


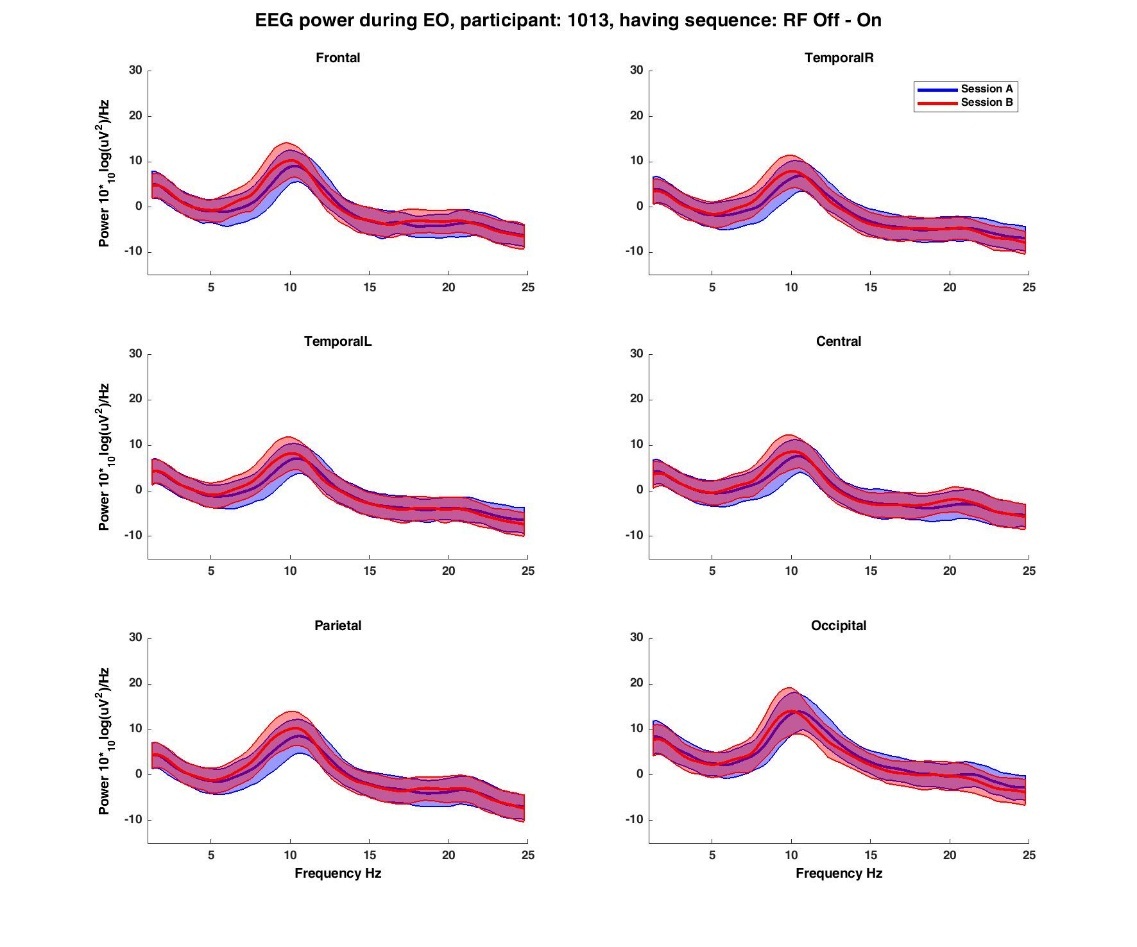


Figure S55. PSDs of participant 1013 for six brain regions in EO condition.


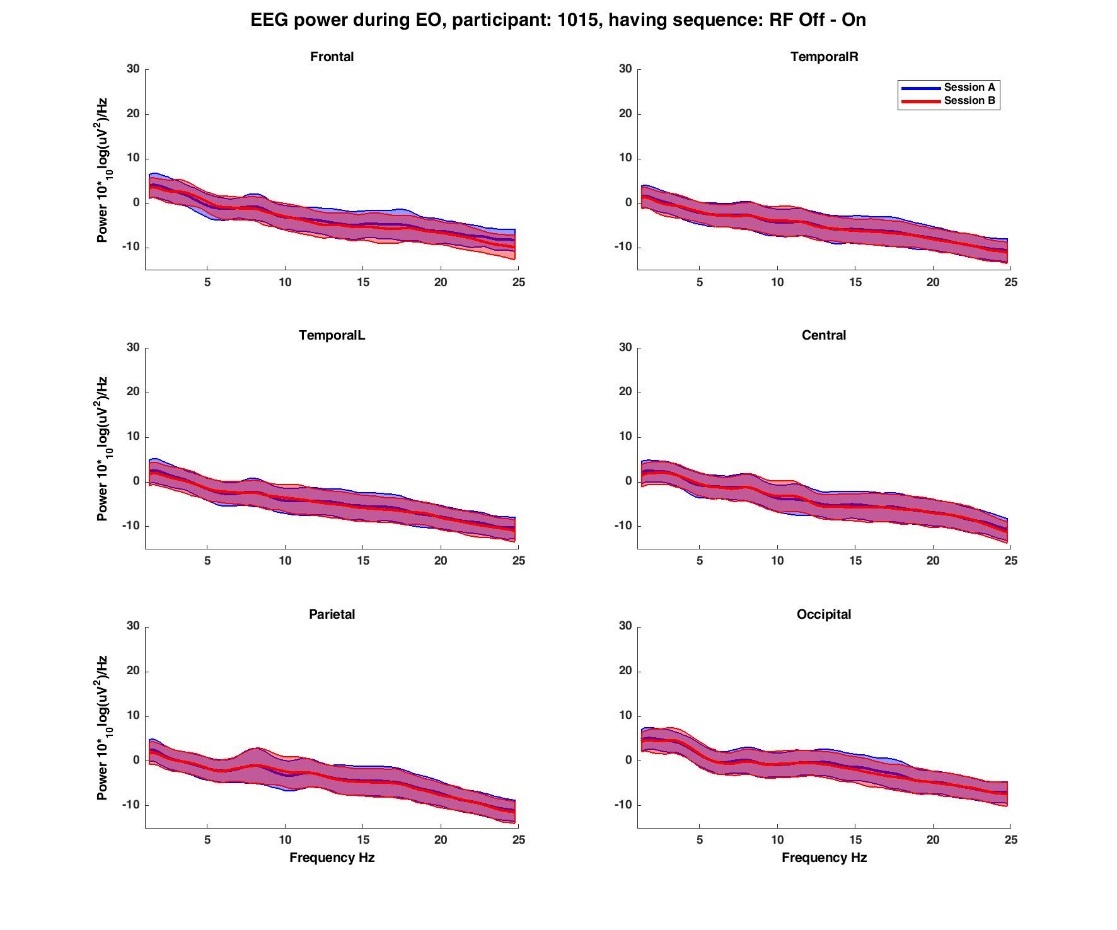


Figure S56. PSDs of participant 1015 for six brain regions in EO condition.


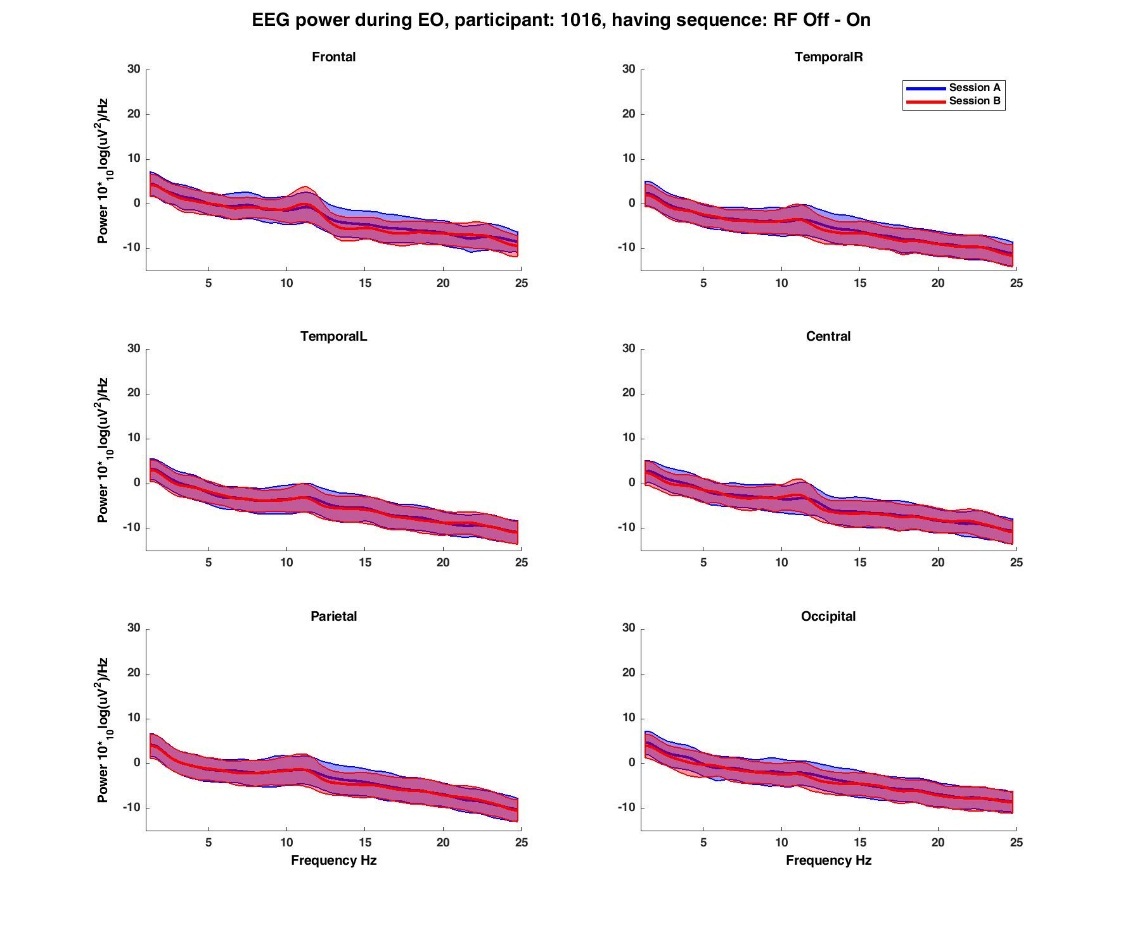


Figure S57. PSDs of participant 1016 for six brain regions in EO condition.


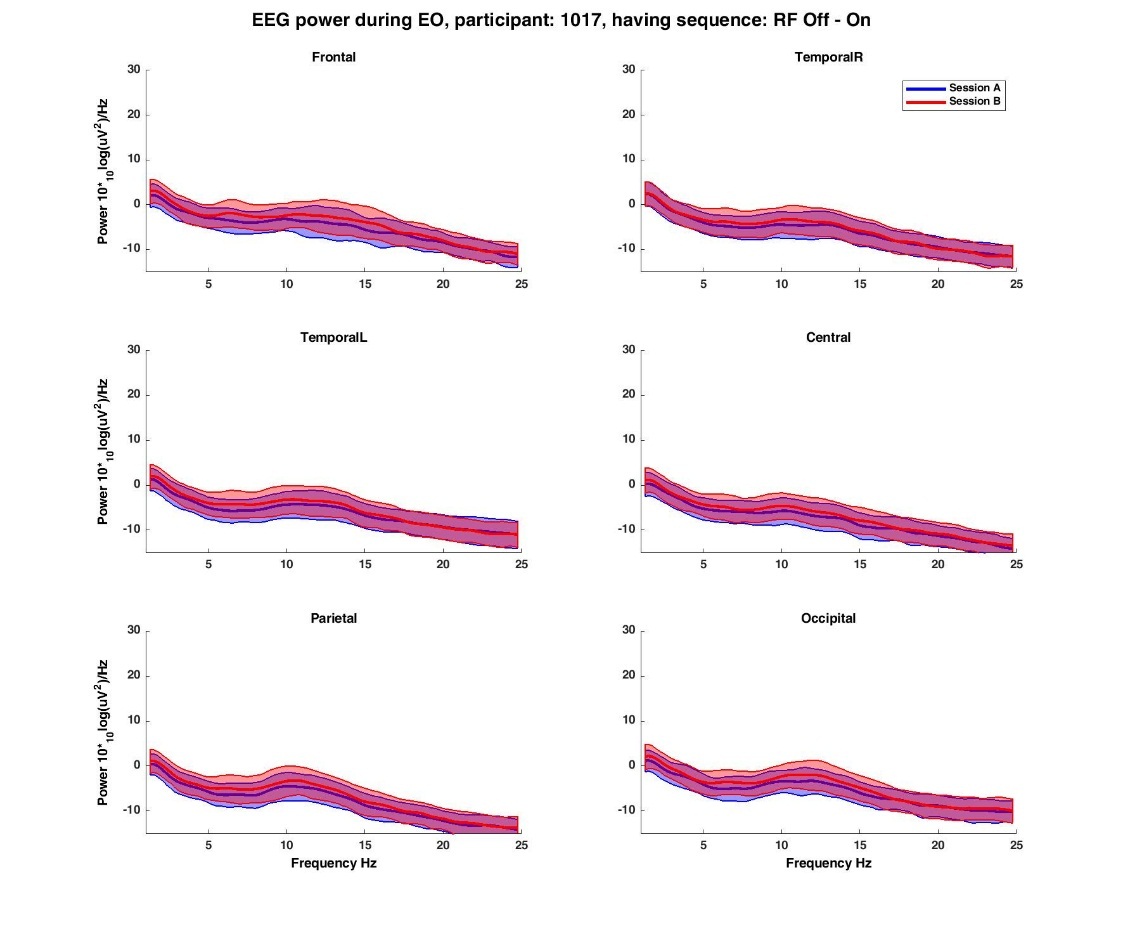


Figure S58. PSDs of participant 1017 for six brain regions in EO condition.


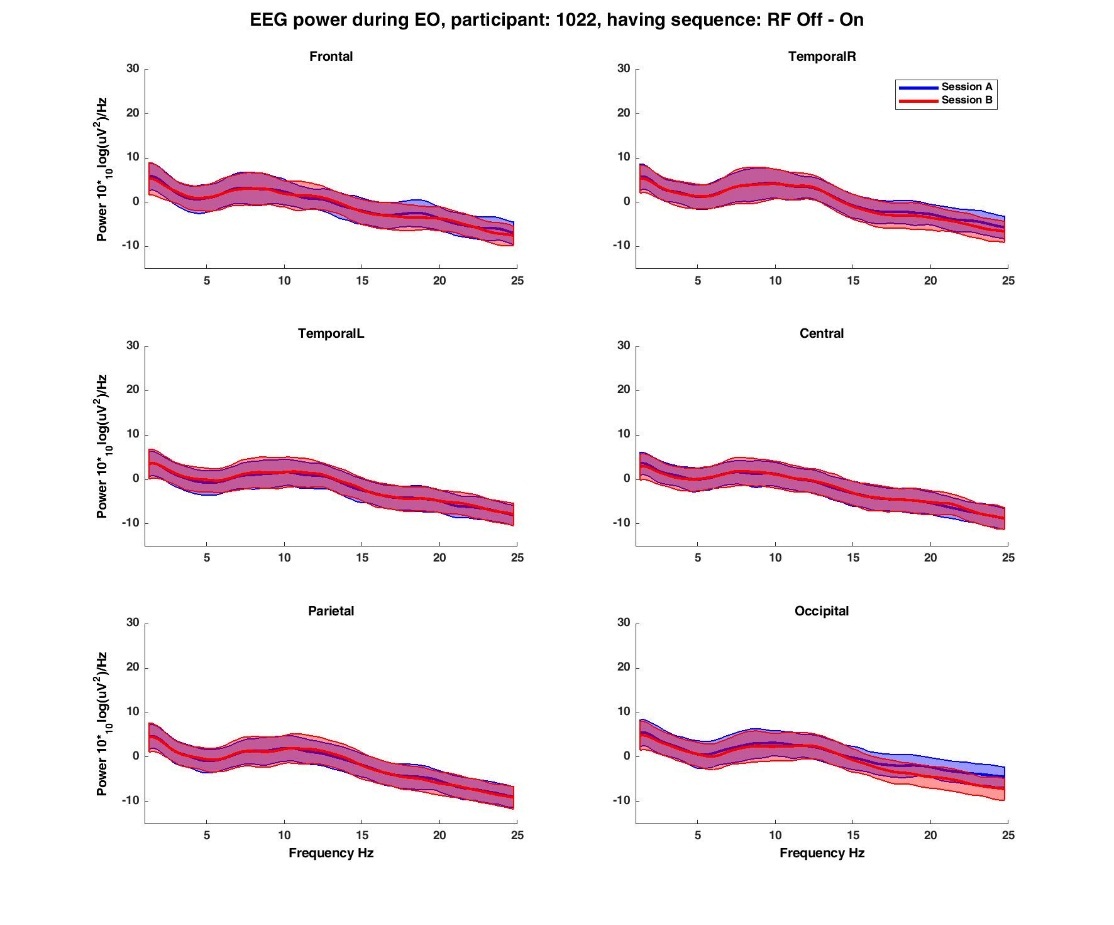


Figure S59. PSDs of participant 1022 for six brain regions in EO condition.


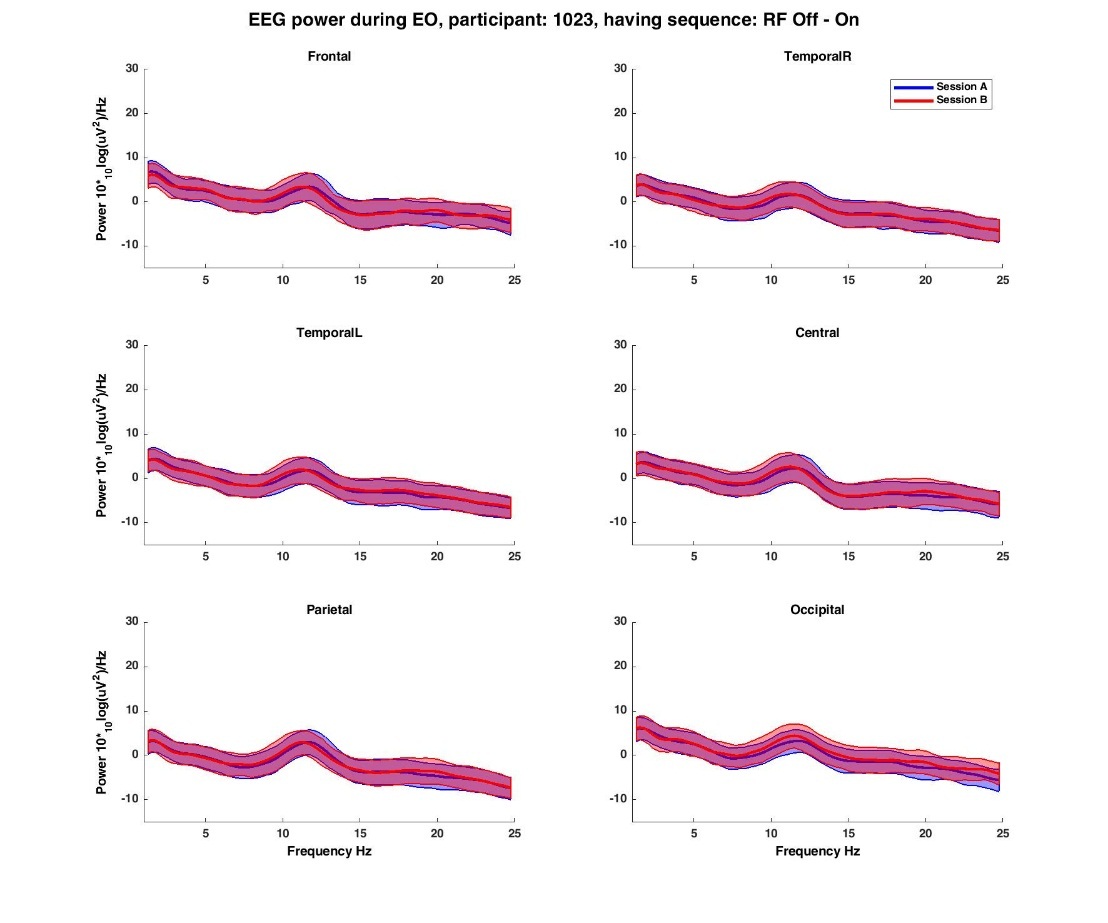


Figure S60. PSDs of participant 1023 for six brain regions in EO condition.


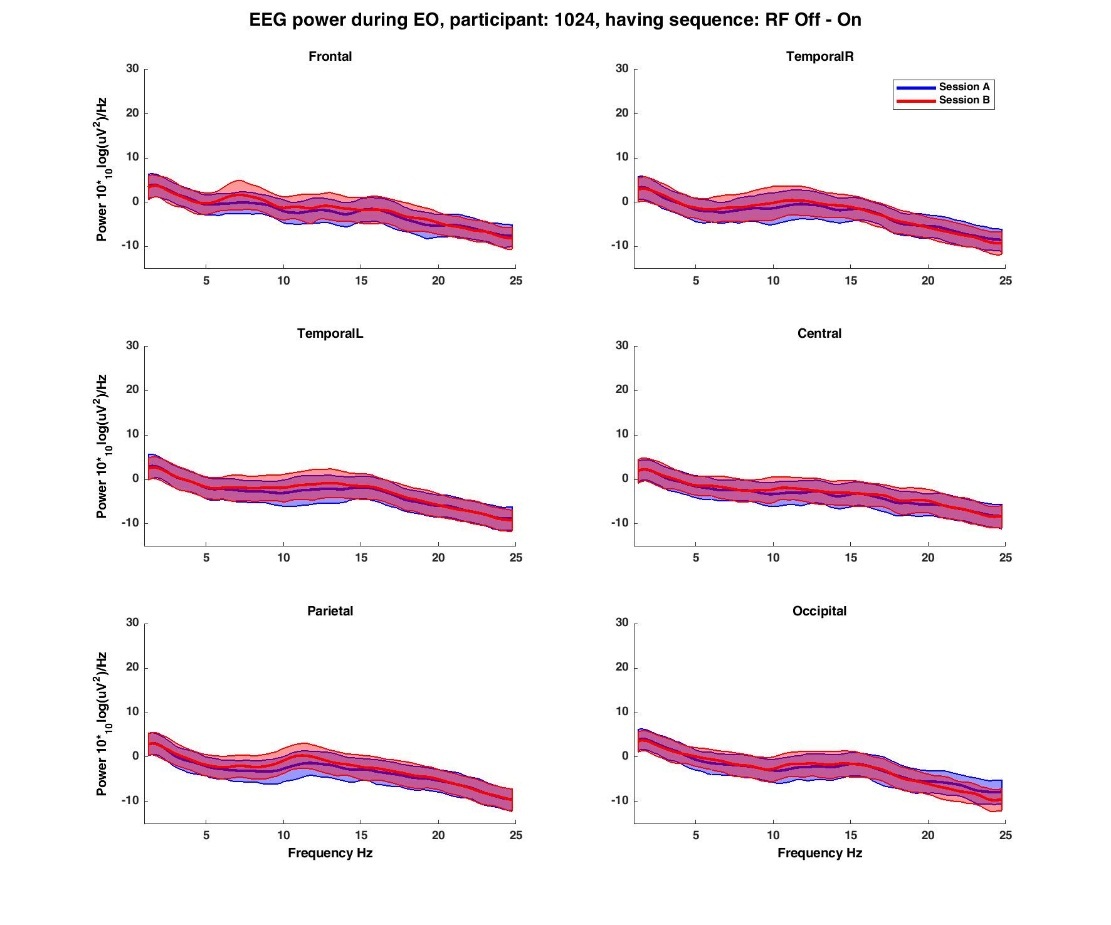


Figure S61. PSDs of participant 1024 for six brain regions in EO condition.


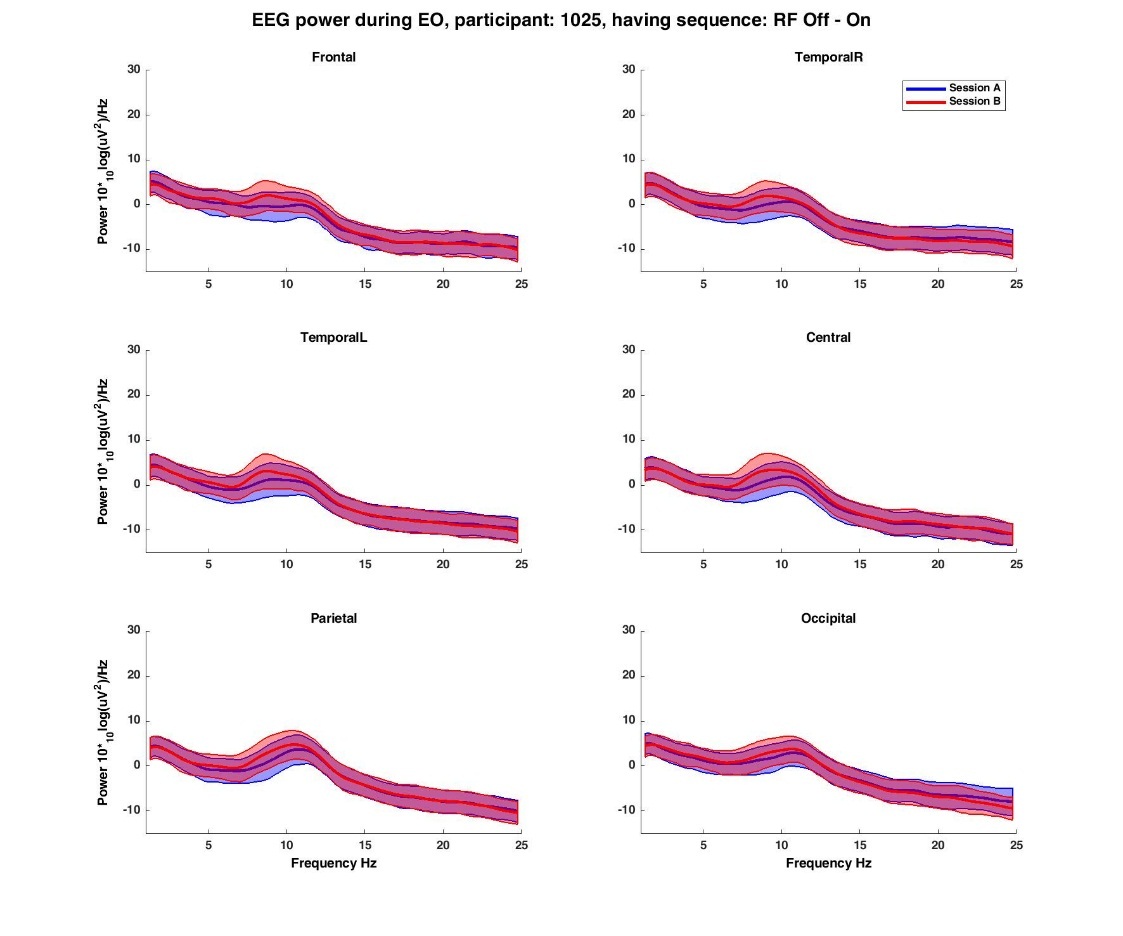


Figure S61. PSDs of participant 1025 for six brain regions in EO condition.


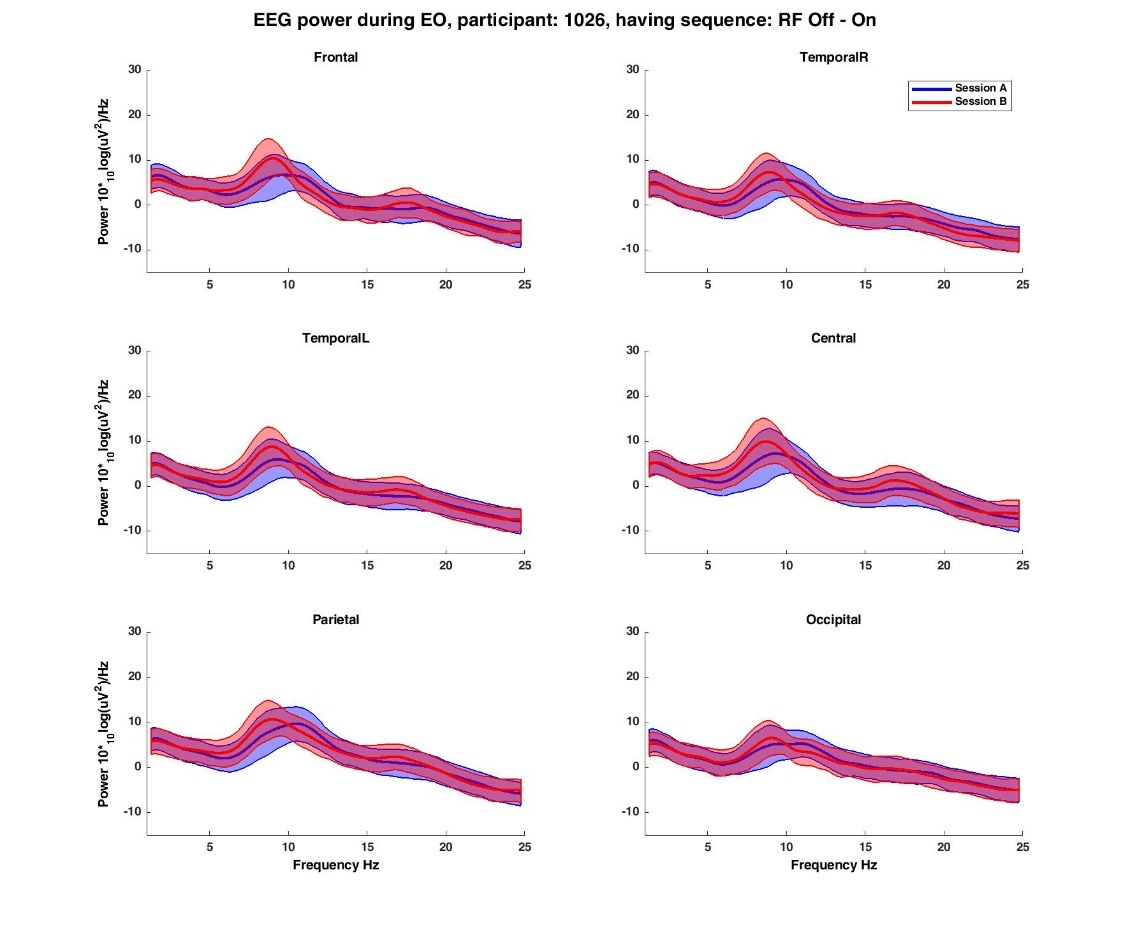


Figure S63. PSDs of participant 1026 for six brain regions in EO condition.


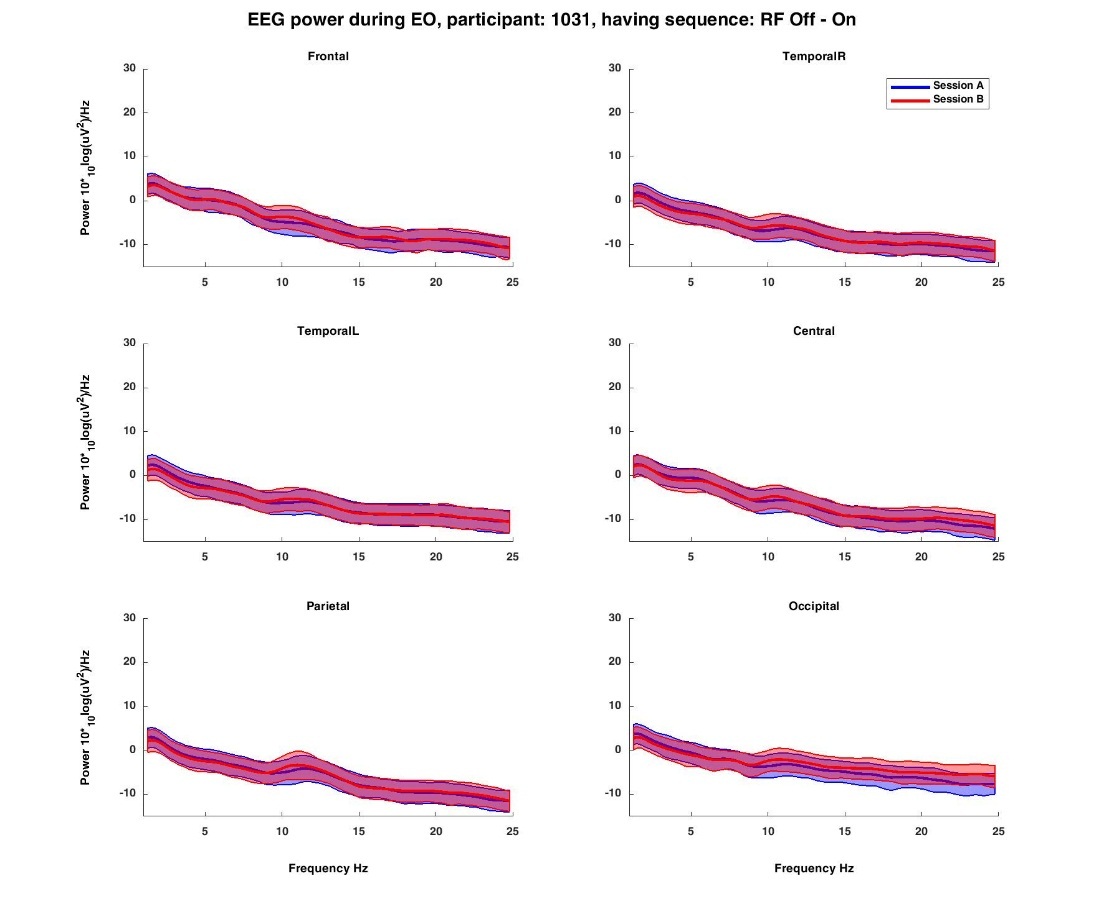


Figure S64. PSDs of participant 1031 for six brain regions in EO condition.

**Appendix C: Validation of Bayesian priors**

In this section, we show the Bayes inference using different priors as mentioned in the method section.

| Normal (0,1) & Uniform (0,5) | | | | | |
| --- | --- | --- | --- | --- | --- |
|  | Group * location | Location | Group | Mean  + sigma | Only  sigma |
| Group * location | 1 | 4.84e-05 | 8.23e+05 | 137 | 2.71e-10 |
| Location |  | 1 | 1.70e+10 | 2.83e+03 | 5.60e-06 |
| Group |  |  | 1 | 1.67e-07 | 3.29e-16 |
| Mean  + sigma |  |  |  | 1 | 1.98e-09 |
| Only  sigma |  |  |  |  | 1 |

Table S1: LRs for comparing five models with prior parameters for the PSD contrast: Normal (0,1) and prior overall sigma parameter: Uniform (0,5).

| Normal (0,1) & Gamma (2,2) | | | | | |
| --- | --- | --- | --- | --- | --- |
|  | Group * location | Location | Group | Mean  + sigma | Only  sigma |
| Group * location | 1 | 4.55e-05 | 8.32e+05 | 0.130 | 2.32e-10 |
| Location |  | 1 | 1.83e+10 | 2.85e+03 | 5.09e-06 |
| Group |  |  | 1 | 1.56e-07 | 2.78e-16 |
| Mean  + sigma |  |  |  | 1 | 1.78e-09 |
| Only  sigma |  |  |  |  | 1 |

Table S2: LRs for comparing five models with prior parameters for the PSD contrast: Normal (0,1) and prior overall sigma parameter: Gamma (2,2).

| Normal (0,1) & Half-normal (0,2) | | | | | |
| --- | --- | --- | --- | --- | --- |
|  | Group * location | Location | Group | Mean  + sigma | Only  sigma |
| Group * location | 1 | 4.77e-05 | 8.26e+05 | 0.135 | 2.61e-10 |
| Location |  | 1 | 1.73e+10 | 2.84e+03 | 5.47e-06 |
| Group |  |  | 1 | 1.64e-07 | 3.15e-16 |
| Mean  + sigma |  |  | 6.11e+06 | 1 | 1.93e-09 |
| Only  sigma |  |  | 3.17e+15 | 5.19e+08 | 1 |

Table S3: LRs for comparing five models with prior parameters for the PSD contrast: Normal (0,1) and prior overall sigma parameter: Half-normal (0,2).

| Uniform (-8,8) & Uniform (0,5) | | | | | |
| --- | --- | --- | --- | --- | --- |
|  | Group * location | Location | Group | Mean  + sigma | Only  sigma |
| Group * location | 1 | 1.57 | 5.07e+13 | 4.11e+07 | 0.466 |
| Location |  | 1 | 3.24e+13 | 2.63e+07 | 0.298 |
| Group |  |  | 1 | 8.10e-07 | 9.19e-15 |
| Mean  + sigma |  |  |  | 1 | 1.13e-08 |
| Only  sigma |  |  |  |  | 1 |

Table S4: LRs for comparing five models with prior parameters for the PSD contrast: Uniform (-8,8) and prior overall sigma parameter: Uniform (0,5).

| Uniform (-8,8) & Gamma (2,2) | | | | | |
| --- | --- | --- | --- | --- | --- |
|  | Group * location | Location | Group | Mean  + sigma | Only  sigma |
| Group * location | 1 | 1.48 | 5.14e+13 | 3.91e+07 | 0.399 |
| Location |  | 1 | 3.48e+13 | 2.65e+07 | 0.271 |
| Group |  |  | 1 | 7.60e-07 | 7.77e-15 |
| Mean  + sigma |  |  |  | 1 | 1.02e-08 |
| Only  sigma |  |  |  |  | 1 |

Table S5: LRs for comparing five models with prior parameters for the PSD contrast: Uniform (-8,8) and prior overall sigma parameter: Gamma (2,2).

| Uniform (-8,8) & Half-normal (0,2) | | | | | |
| --- | --- | --- | --- | --- | --- |
|  | Group * location | Location | Group | Mean  + sigma | Only  sigma |
| Group * location | 1 | 1.54 | 5.10e+13 | 4.06e+07 | 0.449 |
| Location |  | 1 | 3.30e+13 | 2.63e+07 | 0.291 |
| Group |  |  | 1 | 7.96e-07 | 8.80e-15 |
| Mean  + sigma |  |  |  | 1 | 1.10e-08 |
| Only  sigma |  |  |  | 9.05e+07 | 1 |

Table S6: LRs for comparing five models with prior parameters for the PSD contrast: Uniform (-8,8) and prior overall sigma parameter: Half-normal (0,2).

| Cauchy (0,1) & Uniform (0,5) | | | | | |
| --- | --- | --- | --- | --- | --- |
|  | Group * location | Location | Group | Mean  + sigma | Only  sigma |
| Group * location | 1 | 2.79e-04 | 1.12e+07 | 2.72 | 7.33e-09 |
| Location |  | 1 | 4.00e+10 | 9.75e+03 | 2.63e-05 |
| Group |  |  | 1 | 2.44e-07 | 6.58e-16 |
| Mean  + sigma |  |  |  | 1 | 2.70e-09 |
| Only  sigma |  |  |  |  | 1 |

Table S7: LRs for comparing five models with prior parameters for the PSD contrast: Cauchy (0,1) and prior overall sigma parameter: Uniform (0,5).

| Cauchy (0,1) & Gamma (2,2) | | | | | |
| --- | --- | --- | --- | --- | --- |
|  | Group * location | Location | Group | Mean  + sigma | Only  sigma |
| Group * location | 1 | 2.62e-04 | 1.13e+07 | 2.58 | 6.27e-09 |
| Location |  | 1 | 4.30e+10 | 9.82e+03 | 2.39e-05 |
| Group |  |  | 1 | 2.29e-07 | 5.56e-16 |
| Mean  + sigma |  |  |  | 1 | 2.43e-09 |
| Only  sigma |  |  |  |  | 1 |

Table S8: LRs for comparing five models with prior parameters for the PSD contrast: Cauchy (0,1) and prior overall sigma parameter: Gamma (2,2).

| Cauchy (0,1) & Half-normal (0,2) | | | | | |
| --- | --- | --- | --- | --- | --- |
|  | Group * location | Location | Group | Mean  + sigma | Only  sigma |
| Group * location | 1 | 2.74e-04 | 1.12e+07 | 2.68 | 7.05e-09 |
| Location |  | 1 | 4.08e+10 | 9.77e+03 | 2.57e-05 |
| Group |  |  | 1 | 2.40e-07 | 6.30e-16 |
| Mean  + sigma |  |  |  | 1 | 2.63e-09 |
| Only  sigma |  |  |  |  | 1 |

Table S9: LRs for comparing five models with prior parameters for the PSD contrast: Cauchy (0,1) and prior overall sigma parameter: Half-normal (0,2).

**Appendix D: Average short break length**

The EO/EC task consisted of 5 runs of 3 minutes each (90 seconds of eyes open, 90 seconds of eyes closed), with short breaks in between. Breaks were self-paced in order to make the experiment more natural for participants to follow through. While most participants were relatively fast (breaks between 5-20 seconds), other participants were a bit slower. We here present an overview of the break lengths for each participant.


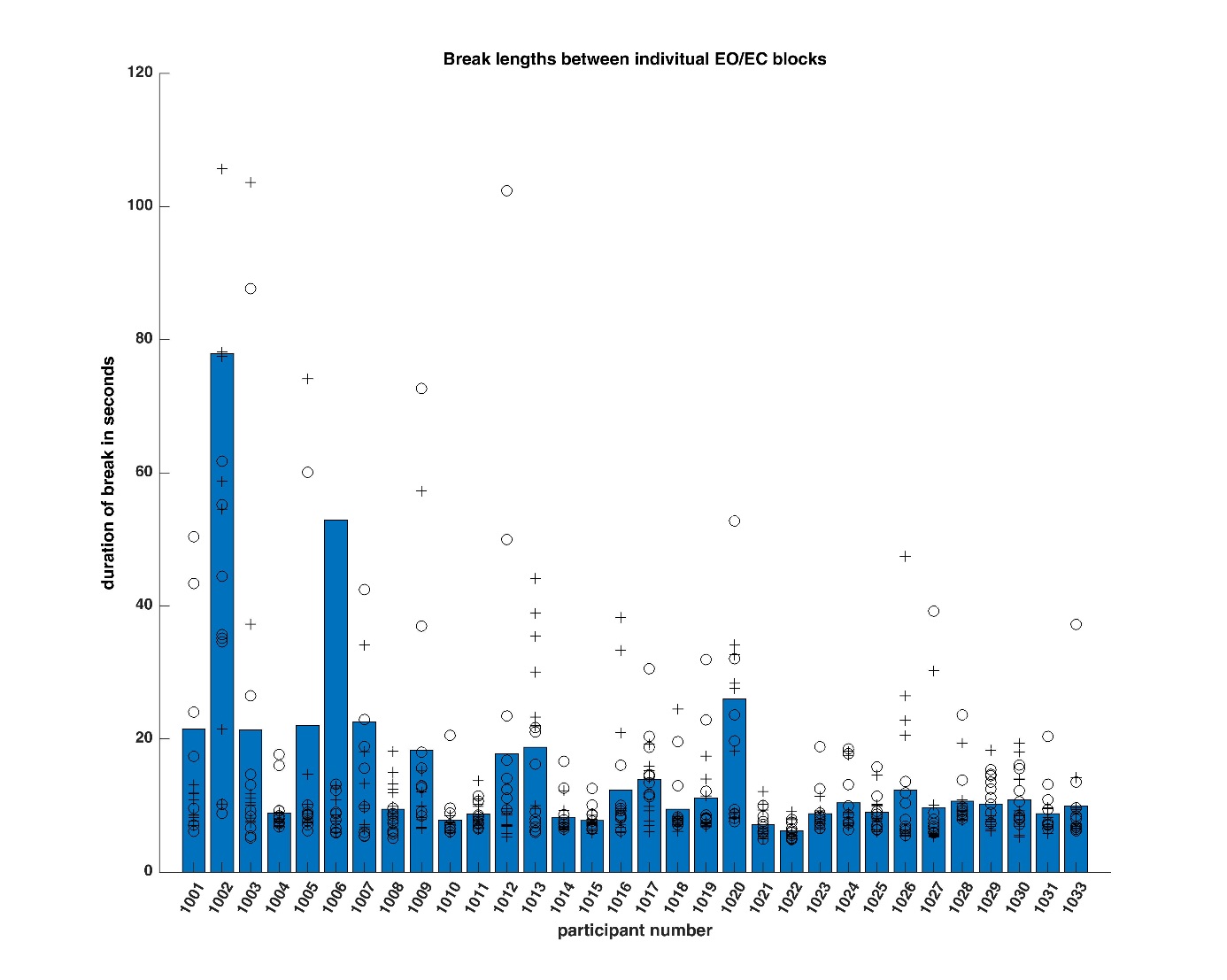


Figure S65: Break lengths for each participant, and for each session. The blue bar represent the average across 18 breaks; the individual data points for breaks during session A are represented with black square markers; the points for breaks during session B are represented with black plus markers.

**Appendix E: Between-subject variability**

The between-subject variability displayed by our participants is higher than the between-session variability used to assess the effects of the EMF. The following figure displays the same power PSD estimates as in Figure 5 of the main paper, but now overlaid with each individual’s PSD during session A and session B.

**
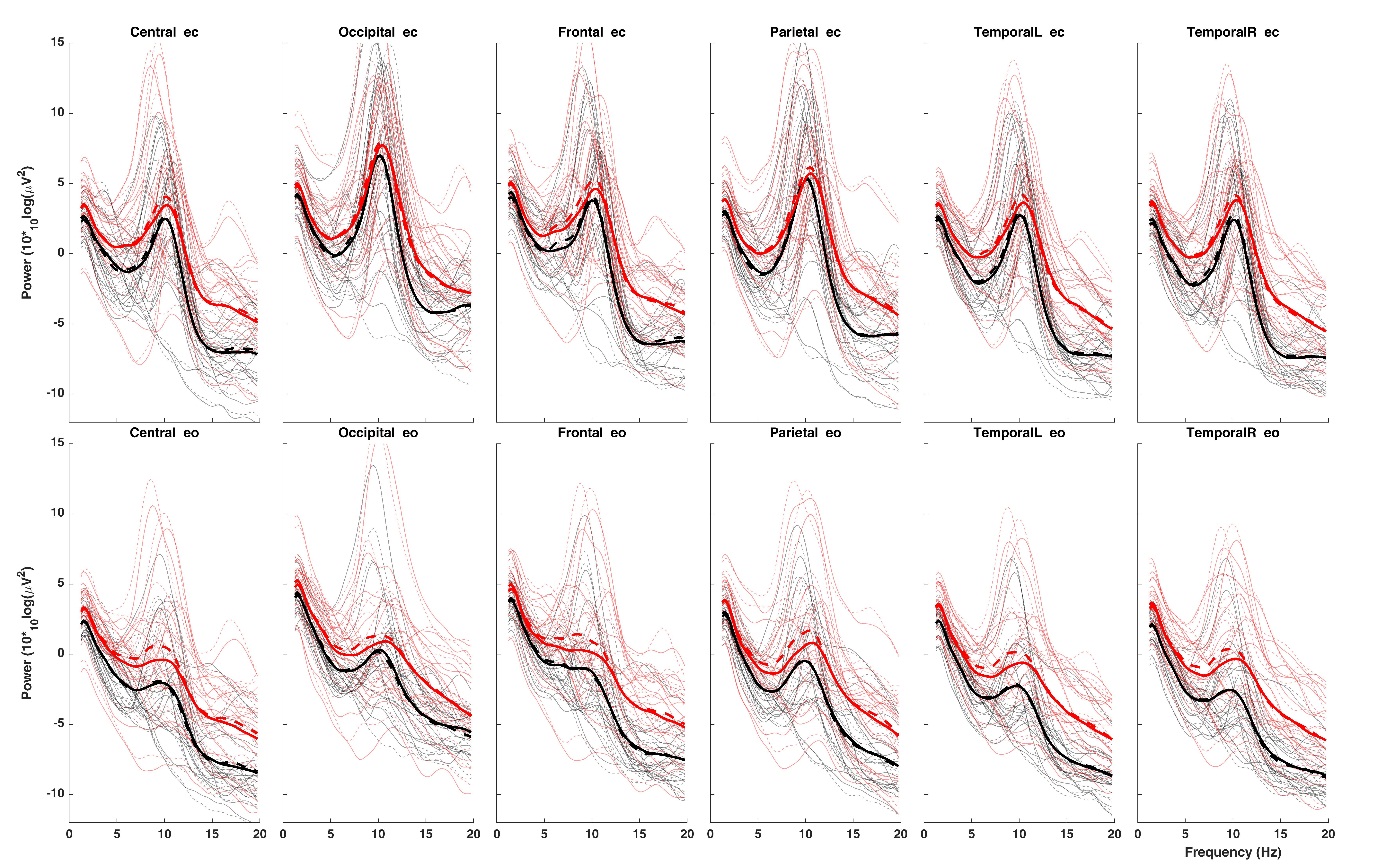
** Figure S66. PSD plots in six brain regions for EC and EO conditions, now overlaid with individual’s plots. Black lines indicate average PSD over 16 participants in group 1 (receiving RF in session A and sham in session B); red lines average PSD over 16 in participants group 2 (receiving RF in session B and sham in session A). The thick lines are the averages (same as in Figure 5 of the manuscript); the thinner lines are each individual’s PSD; the continuous lines are the PSDs from session A; the dashed lines are the PSD’s from session B.
